# Free Papers

**DOI:** 10.1155/1992/21813

**Published:** 1992


### F001

### CHOLEDOCODUOENOSTOMY OR HEPATICOJEJUNOSTOMY IN CHOLEDOCOLITHIASIS: A RANDOMIZED CONTROLLED TRIAL.

G ZEITOUN and A. FINGERHUT, F. LACAINE, D. BRISSET, P.L. FAGNIEZ, J.M. HAY

When biliary enteric anastomosis is indicated for choledocholithiasis, the surgeon has the choice between choledocoduodenostomy (CD), which carries a risk of cholangitis by reflux of duodenal contents, and hepaticojejunostomy (HJ), which is more time consuming and precludes further endoscopic exploration. As of yet, one technique has not been shown to be superior to the other in controlled trials. Between January 1978 and November 1990, 130 patients (31 males and 99 females), 61 ± 15 (m ± sd) years old were enrolled in this randomized study. CD was performed in 64 patients, while HJ was performed in 66 (side-to-side in 25 cases and end-to-side in 41). The major end point was the occurrence of cholangitis within the 3 years following the operation as defined as biological cholestasis associated or not with fever or pain. Other criteria were postoperative mortality, morbidity, and reoperations on the main bile duct. Postoperative mortality was 3.8% (5 patients), without any statistical difference between the 2 groups (4 in the CD group vs 1 in the HJ group ). Postoperative morbidity was not different: 1 biliary fistula in the CD group (1.6%) vs 5 in the HJ group (7.5%). Late results in 120 patients (mean follow-up: 55 ± 11 months) showed that: a) 107 patients (89.2%) had no symptoms related to biliary-enteric anastomosis; b) cholangitis episodes occurred in 13 patients (10.8%) (6 in the CD group and 7 in the HJ group, NS); c) cholangitis occurred during the 1st year in 8 patients (61%) and during the 2nd year in 5 patients (39%); d) no marginal ulcer was found in the HJ group. This randomized controlled trial confirms the good results of CD and HJ in choledocholithiasis, with similar mortality and morbidity rates in both groups, and 90% asymptomatic patients in the long term follow-up. Nonetheless, these results favor the use of CD in choledocolithiasis, because CD is less time-consuming than HJ, and does not preclude further endoscopic exploration.

### F002

### STRESS RESPONSE AND LUNG FUNCTION AFTER CLASSICAL AND LAPAROSCOPIC CHOLECYSTECTOMY

J.RINGERS, B.M.P. RADEMAKER, J.A. ODOOM, L.TH. DE WIT
Dept. Anaest. and Surg., Academic Medical Center, Amsterdam, The Netherlands

In this study the effects of classical cholecystectomy (CC) and LPC on lung function and stress response were compared. Secondly the effect of general anesthesia with or without thoracic epidural anesthesia (TA) were compared. Thirty patients were randomly allocated into three groups; 1-CC, 2-LPC, 3-LPC+TA. Peak expiratory flow (PEF) forced expiratory flow FVC and forced expiratory flow in one second (FeV1) were measured pre–, 2, 4, 8, and 24 hours postoperatively. Arterial blood samples were taken pre and postoperatively to assess plasma levels of cortisol and glucose. Data are expressed as mean ± SD. Results were analyzed with repeated measures ANOVA and Wilcoxin-signed rank test. P values <0.05 were considered significant.

Results: The groups were similar with respect to height, weight, duration of the operation and age of the patients. Two hours post-operatively FVC decreased significantly in all groups compared to preoperative values. Group-1: 3.8±0.94L to 1.07±0.27L (P<0.01). Group-2: 3.63+1.46L to 2.13±0.4L (P<0.05). Group-3: 3.76±0.92L to 2.84±0.90L(P<0.05). In group-1 the reduction was larger than in Group-2 and 3 (P<0.05). Differences between Group-2 and 3 were not significant. These changes remained unaltered for 24 hours. Similar changes were observed with FeV1 and peakflow measurements. FVC to FeV1 remained unchanged. After surgery levels of glucose and cortisol increased in all groups, but levels remained unchanged in group 3 after 120 min. Glucose levels after induction of anesthesia and the epidural catheter were higher in group 3 (P<0.05).

Conclusion: During LPC the endocrine metabolic response is not abolished. Thoracic epidural anesthesia alters this response but does not significantly influence pulmonary function. The reduction in pulmonary function was less with LPC as compared with CC.LPC should be the treatment of choice in pulmonary compromized patients since the reduction in pulmonary function is less in LPC, compared to CC.

### F003

### FINAL REPORT OF RANDOMIZED TRIAL COMPARING DISTAL SPLENO-RENAL SHUNT AND SCLEROTHERAPY IN THE PREVENTION OF VARICEAL REBLEEDING.

G.P. SPINA, R. SANTAMBROGIO, E. OPOCHER, B. RASHIDI, F. COSENTINO, E. MORANDI, R. MOTTA
University of Milan, San Paolo, Institute of Biomedical Sciences, Milan, Italy

In 1984 we initiated a prospective controlled trial comparing endoscopic sclerotherapy (ES) with the distal spleno-renal shunt (DSRS) in the elective treatment of variceal hemorrhage in cirrhotic patients. The study comprised 80 patients (Child A–B) assigned to one of the 2 groups according to random number table: 40 to DSRS and 40 to ES. During the postoperative period, no DSRS patient died, while 1 ES patient died of uncontrolled hemorrhage Rebleeding: 2 DSRS patients (1 from varices, 1 from duodenal ulcer) and 8 ES patients (4 from varices, 4 from esophageal ulceration). Long-term follow-up was complete in 100% pf patients. However, in the ES group, 1 patient was successfully submitted to liver transplantation and another to shunt surgery. 5 years survival rate, was 83% for DSRS and 36% for ES groups (p=N.S.). The global percentage of rebleeding was 5% in DSRS patients (2 from duodenal ulcer) 38% for ES patients (3 from varices, 3 from hypertensive gastropathy and 1 from an unknown source p=0.0028). 3 DSRS and 2 ES patients suffered mild chronic encephalopathy (p=N.S.). This trial seems to indicate that DSRS, in patients with good liver function, is more effective that ES in preventing gastroesophageal rebleeding, without serious sequela and with a tendency to improve survival.

### F004

### DNA-ANALYSIS, ONCOGENE EXPRESSION AND CATHEPSIN D FOR PROGNOSTIC EVALUATION IN SURGICAL PANCREATIC CANCER PATIENTS

J.W. HEISE, H. BOJAR, F. BORCHARD, H. BECKER, S. THOMSEN, H.-D RÖHER
Department of General and Trauma Surgery, Institute of Oncological Biochemistry and Pathology, Heinrich-Heine-University, Düsseldorf, Germany

Disappointing survival rates, even after extensive resectional treatment of pancreatic cancer patients call for more differentiated and sufficient prognostic tools than TNM classification or histopathological grading are at present. Aim of this study was to evaluate a set of new immunohistochemical and molecularbiological parameters in patients operated for pancreatic malignancies in order to develop an individual oncologic risk profile.

In 42 patients laparotomized for ductal (n=33) or other periampullary (n=9) carcinomas the following parameters were determined in fresh tumor tissue: Status of ploidy, percentage of proliferating cells by monoclonal antibody Ki-67, EGF-receptor and cerbB-2 oncogene expression, and content of the protease cathepsin-D. Results were correlated with the kind of tumor, TNM-status, grading, and survival.

The actual median survival was only 98±33 days in the hypodiploid group, compared to 215 ± 84 and 229 ± 51 of the diploid and triploid tumors, respectively. Percentage of Ki-67 positive cells was 19.2 ± 3.4% in ductal adenocarcinoma as compared to 8.2 ± 1.8% in periampullary cancer. Five of 10 patients with distant metastases showed a strong EGF-receptor expression but only 2/30 in case of local disease. Cathepsin-D content was 99.6 ± 15.7 in Tx Nx M1 but only 58.2 ± 6.6 in Tx Nx M0 cases.

The parameters evaluated proved to be discriminitive for survival and biological behavior of the tumor. Thus, they might be useful to define subgroups of patients benefitting of adjuvant or additive palliative treatment modalities.

### F005

### RATIONALE FOR PYLORUS-PRESERVING PANCREATO-DUODENECTOMY *vs* WHIPPLE OPERATION IN THE TREATMENT OF MALIGNANT PERIAMPULLARY TUMORS

H. BECKER, J. W. HEISE, F. BORCHARD, H.-D. RÖHER
Department of General and Trauma Surgery,
Institute of Pathology-Heinrich-Heine University, Düsseldorf, Germany

Whipple's operation in many institutions still is the golden standard for resective surgery in periampullary tumors. Recently the pylorus-preserving pancreatoduodenectomy (PPPD) became increasingly popular. Goal of the present study was to provide rationales for choosing PPPD rather than the Whipple procedure in resection of malignancies of the periampullary region.

From 4/'86 to 11/'91 a total of 55 patients with malignant periampullary lesions underwent pancreatic head resection, either Whipple (n=37) or PPPD (n=18). The ratio of ductal adenocarcinoma to other periampullary tumors was 31/6 and 9/8, respectively.

Mortality was 2/37 (5.4%) after Whipple procedure and 0/18 following PPPD. Morbidity in the latter group consisted mainly in delayed gastric emptying in 7/18 (39%) cases, but needed reintervention in any instance. Histopathological examination of all Whipple specimens did show node involvement above the pyloric level along the lesser and greater curvature in only 1/37 (2.7%).

We conclude PPPD to be the surgical technique of choice in the vast majority of resectable malignancies of the pancreatic head region. Up to now zero mortality·and lack of histopathological evidence, that removal of the distal stomach improves local tumor clearance probably will restrict the classic Whipple procedure to few cases, where the tumor is otherwise technically not resectable.

### F006

### DIGESTIVE AND NUTRITIONAL CONSEQUENCES FOLLOWING PANCREATIC RESECTION: THE ROLE OF PYLORIC PRESERVATION

R. BELLANTONE, G.B. DOGLIETTO, D. FRONTERA, A. FERRANTE, G. VIOLA, F. CRUCITTI
Department of Surgery, Catholic University School of Medicine, Rome, Italy.

The digestive function and nutritional status of 13 patients (8 cephalic pancreatectomies- CP, 5 total pancreatectomies- TP) submitted to pylorus-sparing pancreatic resection for periampullary cancer have been studied; the results have been compared with those obtained in a "traditional" (associated gastrectomy) resection group (8 CP, 5 TP).

Each patient, free of neoplastic recurrence, underwent, 1 year after surgery, a nutritional evaluation (weight change, serum total proteins and albumin, lymphocyte count, cholesterolemia, etc.) and specific tests for digestive functioning (glucose absorption test, fatty meal test pancreolauryl test, oxalate test, fecal chimotrypsine, plasma minerals magnesium, phosphate, iron, copper, zinc).

We observed a 69% rate of digestive and absorptive failure in pylorus-spare patients, but only in subclinical terms. 61.5% of gastroresected patient were seen to have malabsorption, and 3 patients were really malnourished. Digestive impairment was more common after TP(100%), and 2 patients out of 5 were heavily malnourished.

Therefore, we think gastro-pyloric preservation may be effective, at least in TP, in reducing post-pancreatectomy digestive consequences.

Moreover, the procedure does not modify, also in our experience (3 patients), the oncological radicality.

### F007

### GASTRIC EMPTYING (GE) AND MYOELECTRIC ACTIVITY IN THE EARLY POSTOPERATIVE PERIOD AFTER PYLORUS PRESERVING PANCREATODUODENECTOMY (PPPD)

W.A.J.J.M. HAAGH, L.M.A. AKKERMANS, H. OBERTOP
Dept. of Surgery, University Hospital Utrecht, The Netherlands

Introduction. Delayed gastric emptying DGE) has been reported as a frequent complication of PPPD. Gastric dysrhythmias have been postulated as a mechanism for this phenomenon. Also pancreatic cancer patients may have DGE prior to surgery. Pre- and early postoperative GE, in combination with gastric myoelectric activitiy have never been adequately studied and are therefore the subject of this presentation.

Patients and methods. GE of a liquid meal (300ml, 375 kcal) was studied in 8 pancreatic and periampullary cancer (PP) patients (4 M, 4 F; mean age 53 ± 21 yrs) using Applied Potential Tomography (APT. Myoelectric activity was measured by surface electrogastrography (EGG) during 1 hr fasting and 1.5 hrs postprandially, and analyzed by computerized power spectrum analysis. 5 of these patients were also studed within the first 2 weeks following PPPD. The same measurements were done in 20 controls (12 M, 8 F; mean age 63 ± 8 yrs).

Results. Gastric emptying was delayed in both pre- and postoperative PP patients compared to controls, the t1/2 being resp. 154.3±137, 139.4±122 and 83.1±32 min (p = N.S.). Gastric dysrhythmias were not observed in the preoperative and postoperative period. The postprandial/fasting power ratio of the postoperative PP patients was significantly decreased compared to normal (1.30±0.92 resp. 6.28±4.82; p=.005), indicating a decrease in gastric motor activity in these patients. Postprandial peak power and power ratio in the postoperative period decreased compared to the preoperative period (p=.06). A significant correlation between pre- and postprandial mean gastric frequency and t1/2 could be demonstrated.

Conclusion. Although many uncontrolable factors may govern GE, myoelectrical abnormalities of the stomach may play a role in the early postoperative gastric dysfunction following PPPD. Slowing of GE prior to surgery cannot be explained by disturbances in gastric myoelectric activity, but may involve tumor-related effects. Gastric dysrhytmias, when present, probably play a role in the development of DGE.

### F008

### OCTREOTIDE IN THE PREVENTION OF PANCREATIC FISTULA AFTER RESECTIVE PANCREATIC SURGERY: A PROSPECTIVE CONTROLLED RANDOMIZED STUDY

M. MONTORSI, M. ZAGO, M. PIVI*, R. PEREGO*, G. PEZZUOLI and the PFOS Study Group
Istituto di Chirurgia Generale – University of Milano, Milano, Italy.
* Direzione Medica Italfarmaco – Milano, Italy.

Prevention of pancreatic fistula remains an important challenge in pancreatic surgery. In order to evaluate the efficacy of Octreotide in the prevention of pancreatic fistula following pancreatic resection, a multicentric controlled randomized double blind study (PFOS) was started in July 1990; patients (pts.) were enrolled in 35 national surgical departments with recognized experience in pancreatic surgery. The design of the study provides peri– and postoperative administration of Octreotide vs. placebo in two groups of 110pts. undergone elective pancreatic resection. Mortality and specific and general morbidity are recorded. At present, 222 pts. were included. 53 (23.9%) of these were not resected and considered drop-out. A preliminary analysis was thus possible on 169 resected pts. (93 males and 76 females, with a mean age of 58.2 years). Among these, 83 (49.1%) had exocrine pancreatic cancer (66 of the head, 14 of the body, 3 of the tail), 10 (5.9%) endocrine pancreatic tumors, 30 (17.7%) periampullary tumors, 23 (13.6%) other abdominal neoplasms requiring pancreatic resection, 23 (13.6%) chronic pancreatitis. The following operations were performed: 119 (70.4%) pancreaticoduodenectomy (68 Whipple, 51 Longmire), 35 (20.7%) left pancreatectomy, 11 (6.4%) subtotal pancreatectomy, 4 (2.3%) enucleations of endocrine tumors. Overall mortality rate was 9.2%, whereas pancreatic fistula appeared in 17 pts. (10%). Other complications specifically related to pancreatic surgery occurred in 35 pts. (20.7%). Definitive results and analysis are expected within February 1992. Notwithstanding improvement in patients' treatment, morbidity and mortality after pancreatic resections still remain a major problem for surgeons. This study will clarify the role of Octreotide in reducing incidence of this complication.

### F009

### PYLORUS PRESERVING PANCREATODUODENECTOMY: TEN YEARS EXPERIENCE WITH 129 CASES

F. MOSCA, P.C. GIULIANOTTI, F. D'ELIA, A. PIETRABISSA, F. SARTONI, F. VATTERONI, T. BALESTRACCI, A. COSTA, A. GUADAGNI, U. BOGGI, A. CAMPATELLI, M. FERRARI, M. OLEGGINI
Istituto di Chirurgia Generale e Sperimentale, Università di Pisa, Italy

In 1978 Traverso and Longmire revived the idea of preserving the pylorus during pancreatoduodenectomy. Since that time a lot of cases have been reported. Our experience with 129 procedures is here reported. Pylorus preserving pancreato duodenectomy (PPPD) does not adversely affect morbidity or survival as compared to Whipple resection even dealing with pancreatic cancer moreover improving the patient post operative quality of life.

129 PPPD (81m–48f), including 9 total pancreatectomies were performed between Jan. 1982 and Jun. 1991. 18 pts had chronic pancreatitis, 68 pancreatic cancer, 26 papillary cancer, 11 common bile duct cancer, 5 duodenal cancer and 1 retroperitoneal lymphoma. The mean age was 63.69+/–11.08 in the neoplastic group and 46.72+/– 10.6 in the chronic pancreatitis group. Operative morbidity rate was 8,85% (4% in the last 3 yrs.), half of the deaths occurring not for abdominal causes. The mortality rate was 3.79% and 6 pts. required reoperation. Postoperative nasogastric suction was maintained for an average of 12.3 days (5–27), in the 22% of pts. a delayed gastric emptying (more than two weeks) was observed (no additional surgery required). Food reintroduction was satisfactory accomplished by the majority of pts. The weight gain average, six months after surgery (excluding pts. with early neoplastic recurrence), was 3.7 kg (some pts. gained 7–10 kg). Specific late complications were observed in 5 pts. : 2 perforations from peptic ulcer requiring surgery (1 during chemotherapy for lymphoma and the other aspirin induced 5 months after surgery), 1 pt. bled from duodenum (42 months after surgery), 2 pts. bled from antral gastritis, all of them were successfully treated with medical therapy.

22 pts. operated on for pancreatic cancer are still alive: 1 is free of recurrence after 9 yrs.; 1 is alive more than 7 yrs. after resection; 2 more than 4 yrs., 2 more than 3 yrs. and 4 more than 1 yrs. (1 with liver metastases). The remaining 12 survivors are between 6 and 12 months from surgery. 43 pts. died by cancer recurrence: the median survival in this group was 10.23 months. Among the 37 pts. resected for periampullary cancer 23 are still alive; the five years actuarial survival has been 67.64%.All pts. operated on for pancreatitis are alive except one, who died for laryngeal cancer; late complications in this group were: 1 stricture of the hepaticojejunostomy, successfully treated with percutaneous dilatation and 1 small bowel subocclusion due to adhesions spontaneously relieved. Pain relief was satisfactory obtained in every pt. Survival analysis, retrospectively comparing 2 homogeneous groups of pts. who underwent resection for pancreatic cancer, failed to demonstrate any significant difference between those in whom a Whipple procedure was performed as compared to the ones resected according to Longmire's technique.

### F010

### PERI-OPERATIVE RENAL TUBULAR DYSFUNCTION IN PATIENTS WITH OBSTRUCTIVE JAUNDICE: THE VALUE OF URINARY ENZYMOLOGICAL STUDIES

D N ANDERSON, I ROTHNIE, J BROOM, R KEENAN, P H WHITING
Departments of Surgery and Clinical Biochemistry,
University of Aberdeen, Aberdeen Royal Infirmary, Aberdeen, Scotland

Urinary enzyme excretion studies can highlight the inadequacy of conventional parameters of renal function, in determining the incidence of peri-operative renal tubular dysfunction in patients with obstructive jaundice.

Using established automated assay techniques the urinary activities of the proximal renal tubular brush border enzymes, gamma-glutamyl transpeptidase (CST) and alanine aminopeptidase (AAP), along with that of the intracellular lysosomel hydrolase N-acetyl-B-D-glucosaminidase (NAG) were measured in the urine of 14 jaundiced patients (group 1: immediately prior to, and 24 hours after biliary surgery. Additional urinary enzyme excretion patterns were studied in 45 non-jaundice patients undergoing minor surgery (group 2), and surgery (group 3).

In group 2, the pre- and post-operative urinary activities of NAG, GGT and AAP did not differ significantly, at 24 ± 16 vs 30 ± 28 U/mmol creatinine; 3.8 ± 1.3 vs 4.1 ± 3.8 U/mmol creatinine and 1.1 ± 0.7 vs 1.3 ± 1.2 U/mmol creatin respectively. However those with obstructive jaundice (group 1) demonstrated, not only a significantly (p<0.01) elevated pre-operative urinary excretion of NAG (236 ± 153 U/mmol creatinine), GGT (6.6 ± 2.5 U/mmol creatinine) and AAP ( 5.8 ± 1.1 U/mmol), but also further significant elevation of NAG (328 ± 182) p<0.01, GGT (11.5 ± 5.0) p<0.05 and AAP (10.1 ± 6.1) p<0.01, activity following surgical intervention. There was no correlation between the duration of the obstructive jaundice nor the degree of hyperbilirubinaemia and the levels of enzymuria detected. Urinary NAG levels were also significantly elevated following aortic reconstructive surgery, 45 ± 38 vs 128 ± 88 U/mmol creatinine p<0.05, though this was less marked than in the jaundiced group. The surgical and anaesthetic insult in these two groups was comparable. The serum creatinine concentration remained static in both the jaundiced (92 ± 32 vs 89 ± 20 umol/1) and the aortic reconstructive (32 ± 49 vs 90 ± 47 umol/1) groups, in the peri-operative period. Urinary enzymology, applied to patients with obstructive jaundice, reveals a significant proximal renal tubular dysfunction compounded by surgical intervention. Serum creatinine concentration, the conventional measurement of renal function, lacks this sensitivity, especially in the early post-operative period, and its diagnostic value is limited.

### F011

### BACTERIAL TRANSLOCATION IN EXPERIMENTAL BILIARY OBSTRUCTION

J.W. DING, R. ANDERSSON, V. SOLTESZ*, S. BENGMARK
Departments of Surgery and Microbiology*, Lund, Sweden

Obstructive jaundice is frequently associated with septic complications and enteric bacteria have been isolated from both the infectious focus and bile in jaundiced patients. The mechanisms by which enteric bacteria translocate from the gut are not clarified, though an impaired reticuloendothelial function in obstructive jaundice has been reported.

Sprague-Dawley rats were subjected to sham operation (n=10) or common bile duct ligation and transection (CBDL; n=35). After 2 weeks, jaundiced animals received either oral administration of physiological saline (n=15), muramyl tripeptide-phosphatidylethanolamine (MTP-PE) liposomes (n=10) or placebo (empty) liposomes, while sham operated rats received physiological saline, 48 h prior to evaluation of enteric bacterial translocation. Both portal and systemic blood, bile and cekal content were collected and cultured aerobically and anaerobically, as well were tissue samples from the liver, spleen and mesenteric lymph nodes (MLN).

Positive MLN cultures in jaundice + saline (7/15; 47%) and jaundice + placebo animals (4/10; 40%) significantly differed (p<0.05) from sham operated animals (1/10; 10%). No positive MLN cultures were seen in jaundiced animals treated with MTP-PE (p<0.05 as compared to other jaundiced animals). One positive liver culture was noted both among sham operated, jaundice + saline and jaundice + placebo animals. Cekal counts (CFU/gm) of E.coli, Lactobacillus acidophilus and aerobic and microaerobic bacteria did not statistically differ, though the number of E. coli tended to be higher in jaundiced animals. Histological examination of the terminal ileum demonstrated subepithelial oedema in all jaundiced animals.

In conclusion, bacterial translocation was demonstrated in rats with 2 weeks of biliary obstruction, which together with the previously reported impaired RES function might explain the increased incidence of septic complications. Oral administration of liposomal MTP-PE prevented bacterial translocation.

### F012

### THE IMPORTANCE OF ANTIENDOTOXIN ANTIBODIES IN PATIENTS WITH OBSTRUCTIVE JAUNDICE

D McCRORY, M I HALLIDAY, G R BARCLAY*, M D McCAIGUE, P J ERWIN, S STEPHENS# B J ROWLANDS
Department of Sugery, The Queen's University of Belfast
*Blood Donor Centre, Edinburgh. # Celltech Ltd. Slough.

Introduction Patients with biliary obstruction have a high mortality and morbidity following surgical intervention. Endotoxin and tumour necrosis factor (TNF) have been implicated in the pathogenesis of these complications but have only been found sporadically in obstructive jaundice. Antibodies are produced in response to endotoxin and persist long after release of an endotoxin pulse. Antiendotoxin antobodies may be a better measurement of chronic endotoxin exposure.

Methodology Preoperative plasma samples were obtained from 19 jaundiced and 11 control patients. TNF was quantified using an ELISA. Endotoxin was measured by a quantitative limulus lysate assay. IgG and IgM anti-core glycolipid (CGL) antibodies were measured using an ELISA.

Results Mean±standard error: Jaundice n=19 Control n=11

| | TNF pg/ml | Endotxin pg/ml | IgM antiCGL median units | IgG antiCGL median units |
|---|---|---|---|---|
| Jaundice | 1.4±1.1 | 16.2±8.1 | 87.9±14.2 | 274.6±55.7* |
| control | 0±0 | 6.7±1.7 | 74.2±12.7 | 113.6±15.2 |

* Significant difference vs controls p<0.05 Mann Whitney

Conclusions The inability to detect significantly increased concentrations of endotoxin and TNF implies that single daily measurements are of little clinical benefit. In contrast the significant rise in IgG antiCGL antibodies indicates chronic exposure to endotoxin. These results emphasise the need for specific antiendotoxin therapies in jaundiced patients.

### F013

### MONOCYTE AND PLASMA LEVELS OF TRANSFORMING GROWTH FACTOR BETA (TGFβ) IN OBSTRUCTIVE JAUNDICE PATIENTS

MCA PUNTIS AND WG JIANG
Department of Surgery, University of Wales, College of Medicine, Cardiff, UK

Transforming growth factor beta(TGFβ) down-regulates some aspects of immunity (T cell, NK cells & humoral immunity). We have investigated TGFβ levels in plasma and monocytes from jaundiced patients.

Plasma and monocyte were separated from jaundiced (n=44, mean bilirubin 179.2 mM/L) and control (n=30, mean bilirubin 9.9 mM/L) patients blood. Monocyte were then stimulated by LPS. The TGFβ levels were measured by the standard CCL64 cell bioassay. Total bile acids in the plasma was measured using an Enzabile kit. Results are shown as mean ±SEM and statistics are by Student T test. (RU=international Reference Unit).

| | Plasma | TGFβ(RU/ml) | Monocyte | TGFβ(RU/ml) |
|---|---|---|---|---|
| | Benign | Malignant | Benign | Malignant |
| Control | 27.1±6.3 | 27.9±8.1 | 33.9±3.9 | 32.7±7.3 |
| Jaundice | 90.6±25.5* | 193.9±33.1*# | 31.5±4.6 | 32.3±2.8 |

*p<0.05 vs control. # vs benign

Plasma TGFβ levels are increased in jaundiced patients and are highest in malignancy. The highest levels (>200 RU/ml) were seen in patients with poorest prognosis. There is no statistical correlation between bilirubin and TGFβ(r=0.17) or between bile salts and TGFβ(r=-0.02).

Conclusion: Patients with obstructive jaundice have significantly increased plasma TFGβ level which is probably not related to monocyte production. This increase shows a relationship to the patients prognosis.

### F014

### SMALL BOWEL MORPHOLOGY IN CONVENTIONAL AND GERM FREE RATS IN OBSTRUCTIVE JAUNDICE

M.S. QURAISHY, D. CHESCOE, R.H. HINTON, M.E. BAILEY
Royal Surrey County Hospital, Guildford and University of Surrey, Guildford, United Kingdom

Increased morbidity in patients undergoing surgery for obstructive jaundice is attribuited to systemic endotoxaemia which may be due to increased absorption secondary to changes in the intestinal epithelium. Small bowel mucosal morphology has been studied in conventional and germ free rats with obstructive jaundice. Methods used included light microscopy, enzyme histochemistry and electron microscopy. The chromogenic limulus amoebycyte lysate assay was used to assess blood endotoxin levels. Significant changes were found in models where obstructive jaundice had been induced. In bile duct ligated animals morphological changes were observed with PAS and alcian blue stains which revealed an increased intensity of staining of the goblet cells. Enzyme histochemisty showed that the brush border activity of alkaline phosphatase was decreased with some regions showing virtually no activity. The activity of the cell membrane marker enzyme 5 nucleotidase was also decreased. Electron microscopy revealed damage to the microvilli, thickening of the rootlets, hypertrophy of the Golgi apparatus, scattered mitochondrial damage and changes in the nuclei and cell membrane of the epithelial cells. Mitochondrial changes were not paralleled by activities of succinic dehydrogenase and cytochrome oxidase which were normal. No morphological differences were found between conventional and germ free animals. The data presented indicates that changes in jejunal morphology are not secondary to endotoxins but are most likely due to the absence of bile.

### F015

### POSTOPERATIVE BILE LEAKAGE: THE ENDOSCOPIC MANAGEMENT

PHP DAVIDS, EAJ RAUWS, K HUIBREGTSE, TM VAN GULIK, MN VAN DER HEYDE, GNJ TYTGAT
HPB-UNIT, Academical Medical Center, Meibergdreef 9, 1105 AZ Amsterdam, The Netherlands

Postoperative bile leakage is a serious complication, in which surgical treatment has its limitations. This study evaluates the endoscopic management.

Patients: Between 1982 and 1990, 55 patients (28 female, 27 male; mean age 55) were referred with postoperative bile leakage. Initial surgery included cholecystectomy in most cases. T-tubes were employed in 17 patients. The patients presented with a biliary-cutaneous fistula (n=27), peritonitis (n=10), recurrent intraabdominal abscess formation (n=5), progressive jaundice (n=7) cholangitis (n=5) or pancreatitis (n=1). The mean interval between initial surgery and presentation was 37 days, (range 5-292). Therapy consisted of standard sphincterotomy, if needed subsequent stone extraction, with or without endoprosthesis placement. The aim of all treatment modalities was to facilitate bile flow into the duodenum.

Results: The biliary tract and the site of the leakage were visualized during ERCP in 98%. The site of leakage was the cystic duct stump (n=37), the CHD (n=6), the CBD (n=6), a hepatic radical (n=4) and a surgical anastomosis (n=1). Distal obstruction due to retained gallstones was present in 15 patients and due to concomitant strictures in 18. Overall, 48 of 55 patients were treated endoscopically: sphincterotomy (n=11), sphincterotomy and stone extraction (n=10), stone extraction and stent placement (n=5), stent placement (n=19) and stent placement followed by nasobiliary drainage. An excellent outcome (clinical and radiological improvement and closure of the bile fistula) was achieved in 43 patients (90%). Despite adequate therapy, 5 patients (10%) had ongoing sepsis resulting in a fatal outcome.

Conclusion: Postoperative bile leakage can be diagnosed effectively by ERCP, and treated safely by subsequent endoscopic management.

### F016

### HIDA-SCINTIGRAPHY AFTER LAPAROSCOPIC CHOLECYSTECTOMY

D.J. GOUMA, P.M.N.Y.H. GO, M.J.P.G. VAN KROONENBURG, H.L.M. PASMANS.
Dept. Surgery and Dept. Nuclear Medicine, University Hospital Maastricht, The Netherlands.

Obstruction of the bile ducts or bile leakage are serious complications after laparoscopic cholecystectomy. HIDA-scintigraphy is a sensitive method to evaluate the bile flow.

The aim of the study is to assess the value of HIDA-scintigraphy to detect biliary complications after laparoscopic cholecystectomy.

In 51 patients a scintigraphy of the bile ducts was performed with Tc99m-dimethylphenyl-carbamoxyl-methyl-IDA (n=8) or Tc99m-trimethylbromo-IDA (n=43) in a dose of 3.5 mCi intravenously, 24 hours after the operation. Anterior abdominal images were made at intervals during 1 hour or longer when necessary. The scintigraphic findings were compared to the clinical course.

No biliary obstructions were noticed. In 5 patients bile leakage was seen on the scintigraphic images. Three patients had no clinical symptoms of bile leakage. In 1 patient bile leakage from the cystic stump was found at laparotomy. In another patient a bile collection was found at re-laparoscopy. Better images were made using Tc99m-trimethylbromo-IDA than Tc99m-dimethylphenyl-carbamoxyl-methyl-IDA. All patients recovered uneventfully.

In conclusion: HIDA-scintigraphy with Tc99m-trimethyl-bromo-IDA is a highly sensitive method for early diagnosis of bile leakage after laparoscopic cholecystectomy, although routine postoperative scintigraphy is not warranted.

### F017

### TREATMENT OF BENIGN BILIARY STRICTURES: SURGERY OR ENDOSCOPY?

AKF TANKA, PHP DAVIDS, TM VAN GULIK, RAUWS EAJ, LT DE WIT, PLM VERBEEK, DJ VAN LEEUWEN, K HUIBREGTSE, GNJ TYTGAT, MN VAN DER HEYDE
HPB-Unit, Academic Medical Center, Amsterdam, The Netherlands

Benign biliary strictures (BBS) remain a therapeutic challenge. This study compares the treatment results of surgery and endoscopy in one institution.

Patients: Between 1981 and 1990, 35 patients were treated surgically (ST) and 66 by endoscopic biliary stenting (ET). In almost all patients the initial trauma occurred during biliary tract surgery. Patient characteristics, previous repairs and level of obstruction were comparable in both groups. ST consisted of constructing a biliary-digestive anstomosis in healthy tissue. ET consisted of placement of endoprostheses, with elective exchange trimonthly for a one year period.

Initial Results: In 35 ST patients a proximal hepaticojejeunostomy (HJ) was performed. Postoperative complications included: septicaemia (n=5), bile leakage (n=2) and major bleeding necessitating relaparotomy (n=2). ET related early complications comprised: minor papillary bleeding (n=1), cholangitis (n=2) and pancreatitis (n=2). During treatment all complications were stent related: cholangitis (n=14), recurrent cholestasis (n=2) and stent migration (n=2). In 46 patients the endoprostheses were eventually removed.

Late Results: After a mean period of follow-up of 50 months (range 10-85), 5 of 35 ST patiens (14%) restrictured. Recurrent stricturing occurred in 8 of 46 ET patients (17%) after a mean period of follow-up of 42 months (range 4-99). Subsequently, 6 patients underwent a HJ and 2 were restented.

Conclusion: Surgical or endoscopic treatment of BBS yields comparable results. Indications for ST are complete transsections, previous repairs and failures of ET. Candidates for ET are those unfit for surgery or presenting with concomitant biliary fistula. In all other patients we advocate ET as the initial treatment.

### F018

### WHY AND WHEN BILE DUCT INJURY OCCURS

TARGARONA EM, GARCIA OLIVARES E, MARCO C
Dept of Surgery, Hosp. Clinic and Hosp. de Mutua de Terrassa, Univ. of Barcelona, Barcelona, Spain

Bile duct injury (BDI) is the most dangerous complication after cholecystectomy. The incidence of BDI usually low (0.2-1%), but it is associated with high morbidity and mortality. Factors that has been considered to be related with BDI are the surgeon skill, variations of the bile duct anatomy, inflammatory local conditions, and intraoperative cholangiography.

Aim: To investigate the clinical features and the predisposing factor for BDI in a consecutive series of 3,051 cholecystectomies.

Patients and Methods: The clinical records of 3,051 patients who underwent choleycystectomy between June 1977 and January 1990 in a surgical unit of a 600 bed teaching hospital were reviewed. BDI was classified as Group I: BDI diagnosed intraoperatively, Group II: BDI diagnosed in the inmediate postoperative time, and Group III: BDI with late presentation. Age, sex, intraoperative diagnosis, surgeon skill (staff or resident), technical difficulty, intraoperative cholangiography, anatomy of the bile duct and the results after surgical repair were evaluated.

Results: During the period of study we observed 26 BDI. 19 BDI occurred after biliary surgery (19/3051, [0.6%]), and 2 after gastric surgery (2/470, [0.4%]). 18 BDI were diagnosed intraoperatively (Group I), 4 in the inmediate postoperative time (Group II), and 4 cases presented months or years (3m-18y) after cholecystectomy (Group III). In 62% of cases, the intraoperative cholangiography demonstrated the BDI. BDI were significantly more frequent in cases operated by staff surgeons than by residents ($p<0.001$), and in cases with a more difficult surgical field ($p>0.005$). 2 patients died after surgical repair of a BDI (2/26, 7.7%). The morbidity rate was 54%. 28% patients developed late complications (cholangitis, setenosis or choledocholithiasis).

Conclusions: 1. BDI occurred more frequently in cases technically difficult and operated by skilled surgeons. 2. Intraoperative cholangiography has been useful for preoperative detection of BDI. 3. The incidence of BDI is low, but it is associated with high morbidity and mortality. These results must be considered when comparing open cholecystectomy with new alternative treatments of cholelithiasis as laparoscopic cholecystectomy.

### F019

### OPERATIVE RISK OF REHEPATECTOMIES FOR LIVER TUMORS

G. BORGONOVO, C. VONS, V. KARAA, D. GRANGE, D. FRANCO, C. SMADJA

Hôpital Antoine Béclère, 92141 Clamart Cédex, Hôpital Louise Michel, 91014 Evry Cédex, France

Rehepatectomies for recurrent primary or secondary liver carcinomas have become more frequent. The purpose of this work was to assess the operative risk of rehepatectomies. Between 1984 and 1991, 295 liver resections have been performed. Twenty-severn resections (9.2%) were rehepatectomies: second resection in 23 patients and third resection in 4 patients. Mean age (16 males and 7 females) was 53 years (range: 18 to 73 years). Ten patients had recurrent metastases from colorectal primary, 7 patients (6 with cirrhosis) had recurrent hepatocellular carcinoma and 6 patients had another kind of tumor. The first liver resection in these patients had been major resection in 9 cases, segmentectomy (1 or 2 segments) in 10 cases and atypical resection in 4. Rehepatectomy was major in 7 patients, segmental in 7, and atypical in 13. Operative mortality was 4.4% (1 patient). Mean operative blood transfusion was $4.5 \pm 5.2$ units of packed red cells, twice the mean blood transfusion in first-hand hepatectomies in our series. Four patients (14.8%) had operative complications: thrombosis of portal vein requiring a portocaval shunt, injury to hepatic duct resulting in T-tube drainage, and coagulation disorders with diffuse bleeding in 2 patients. The latter complication occurred in 2 of the four patients with a third liver resection. Bleeding stopped after packing in one patient with a normal liver. Bleeding resulted in death from exsanguination in the second patient who had liver cirrhosis. Severe operative complications were more frequent in patients with a third hepatectomy (50%) than in patients with a second hepatectomy (8.7%).

These results suggest that the operative risk of rehepatectomy is far more important than that of a first liver resection. Indication of a third hepatectomy should be cautious, particularly in patients with cirrhosis.

### F020

### EFFECT OF PORTAL AND PERIPHERAL SERUM ON CULTURED HEPATOCYTES AND COLONCARCINOMA CELLS AFTER PARTIAL HEPATECTOMY IN RATS

K.P. DE JONG, H.E. LONT, A.M. BIJMA, R. VAN STRIK*, R.L. MARQUET, O.T. TERPSTRA
Dept of Surgery and *Biostatistics, University Hospital, Dr Molewaterplein 40, 3015 GD Rotterdam, The Netherlands

For patients with liver metastases of a colorectal carcinoma (CC) a partial hepatectomy (PH) is the only hope for cure. The majority of patients die however because of recurrences. After PH an overwhelminbg multiplicaiton of cells occurs in the remnant liver in order to restore liver mass and function (regeneration). There is circumstantial evidence that tumor cells are stimulated also during the peak of liver regeneration. The aim of the study is to detect whether portal or peripheral serum contains factors responsible for this phenomenon. In Wag-Rij rats a 70% PH, sham (SH) or no operation (CO) is performed. After various time intervals (24 hours and 14 days) portal and peripheral (right atrial) serum is withdrawn for addition to cell cultures of hepatocytes (H) and CC cells. Portal serum is added to mixed cultures of CC and H. DNA replication is measured by $^3$H-thymidine incorporation, using a scintillation counter producing desintegrations per minute (dpm). Cell cultures of CC cells show a 10 fold increase in dpm if cultured with PH serum taken from rats 24 hours after operation compared to SH or CO serum ($p=0.005$). PH, SH or CO portal serum taken 14 days after the procedure reveals no significant differences. Peripheral serum shows a higher number of dpm comparing portal serum taken 14 days after PH, SH or CO. Using mixed cultures of H and CC cells (ratio 1:1 and 1:10) a significant increase in dpm is found compared to cells cultured separately ($p<0.01$). This potentiating effect is not found if a ratio of 10:1 (CC:H) is used.

Conclusions: Portal serum taken 24 hours after PH contains factors stimulating the growth of CC cells. A potentiating effect can be found if H and CC cells are cultured in conjunction. A minimal amount of H seems to be essential for the interactive stimulation.

**F021**

### SURGICAL APPROACH TO SEGMENT I FOR MALIGNANT TUMORS

D. ELIAS, PH. LASSER, E. DESRUENNES, H. MANKARIOS
Gustave-Roussy Institute Cancer Center, 94805 Villejuif, France

Seven complete and thirteen partial resections of segment I (caudate lobe) were performed for malignant tumors. In all cases except one, removal of segment I was associated with other types of hepatic resection either for technical or carcinological reasons. Six were iterative hepatic resections for recurrent liver metastases. In two cases the future remaining left lobe was pre-operatively hypertrophied by right portal venous embolization. Hepatectomies were performed with intermittent portal triad clamping (mean total duration: 63 minutes, range 20-120) and after preparation for total vascular exclusion. Associated partial resection of the inferior vena cava was necessary in three cases. Mean duration of operation was 285 minutes (range 60-540) and mean blood loss was 1749 milliters (range 200-5200). There was no postoperative mortality and morbidity was low (30%). Surprisingly we retrospectively discovered that free margins were small, less than 5 millimeters in 83% of the cases. In spite of limited free margins and six iterative hepatectomies, eight patients are free of disease with a mean follow-up of 19.2 months. Techonical problems were different for each case and case by case adaptation was necessary. Left, right, and central approaches were used accordingly. If resection of segment I associated with a right or left hepatectomy can be currently considered as a standard major hepatic resection, isolated complete resection of segment I remains a real technical challenge.

**F022**

### HEPATIC ISCHEMIC TOLERANCE: COMPARATIVE ANALYSIS OF WARM AND COLD ISCHEMIA

P. LAMESCH, C.RABE, B. RINGE, G. GUBERNATIS, J. HAUSS,
R. Pichlmayr Klinik für Abdominal und Transplantationschirurgie
Medizinische Hochschule Hannover, 3000 Hannover 61 - FRG

Hepatic ischemic damage was studied in experimental and clinical settings comparing warm ischemia (pringle manoeuvre) and after hypothermic in situ protection.

MATERIAL AND METHODS: A. Experimental study: in 10 pigs (20-25 kg BW) vascular exclusion of the liver and installation of a porto-caval-jugular bypass. E-group 1 (n=5): 2 h warm ischemia; E-group 2 (n=5) hypothermic in situ protection (HTK solution 5°C) and subsequent 2 h ischemia. B. Clinical study: 16 patients (C-group 1) underwent conventional hepatic resections (Pringle maneuvre): ischemic time: 25 min [0-47]. 8 patients (C-group 2) liver resection was performed after vascular exclusion and hypothermic in situ protection (HTK sol.). Ischemic time: 137 min [29-180]. Conventional labarotory parameters were compared in the postoperative course on day 1, 3 and 7. [results given as median; *p<0.05 (U-test)]

| RESULTS: | GOT U/L | | | GLDHU/L | | | Bili umol/l | | | PTT% | | |
|---|---|---|---|---|---|---|---|---|---|---|---|---|
| postop. day | 1 | 3 | 7 | 1 | 3 | 7 | 1 | 3 | 7 | 1 | 3 | 7 |
| E-Group 1 | 449* | 36 | 24 | 15* | 3* | 2* | 13 | 5 | 3 | 45 | 100 | 100* |
| E-group 2 | 127 | 12 | 25 | 5 | 1 | 1 | 8 | 5 | 3 | 82 | 100 | 90 |
| C-group 1 | 163 | 37 | 22 | 101 | 28 | 14 | 58 | 46 | 28 | 56 | 67 | 78 |
| C-group 2 | 399 | 56 | 18 | 181 | 151* | 21* | 65 | 31 | 15* | 63* | 63 | 87 |

CONCLUSIONS: The experimental study showed the significant protective effect of hypothermic in situ protection of the liver compared to warm ischemia. From the clinical results it can be concluded that inspite a 5 fold prolonged ischemic time, ischemic damage (transaminases) was moderately more pronounced, functional parameters (bilirubin, PTT) were less depressed.

**F023**

### WATER-SOLUBLE ETHYLHYDROXYETHYL CELLULOSE PREVENTS BACTERIAL TRANSLOCATION INDUCED BY MAJOR LIVER RESECTION IN THE RAT

X.D. WANG, R. ANDERSSON, V. SOLTESZ*, S. BENGMARK
Departments of Surgery and Medical Microbiology*, Lund, Sweden

Enteric bacteria might act as pathogens, translocating across the intestinal barrier to extraintestinal sites after major liver resection. Methods to decrease the incidence of translocation are thus sought for.

Water-soluble ethylhydroxyethyl cellulose (EHEC) was administered orally or intravenously prior to 70% or 90% hepatectomy in the rat. The influence of enteric bacterial translocation to mesenteric lymph nodes (MLN) and blood, enteric bacterial population and bacterial adherence on the intestinal surface was determined. Phagocytic capacity by visceral and circulating macrophages were determined by the uptake of $^{125}$I-labeled, heat-killed *E. coli*.

Oral or intravenous administration of EHEC reduced the incidence of bacterial translocation to MLN and blood following major liver resection. Oral EHEC appeared more effective than i.v. administration in protecting against bacterial translocation to MLN in animals with 90% hepatectomy. EHEC (oral and i.v.) significantly diminished intestinal macrophage uptake capacity of $^{125}$I-labeled, heat-killed *E. coli* as compared to animals without EHEC administration. Overgrowth or colonization of enteric bacteria following major liver resection could be prevented by both oral and i.v. EHEC. Adherence of $^{14}$C-labeled, alive *E. coli* on the intestinal mucosa decreased following EHEC treatment in animals subjected to major liver resection.

In conclusion, EHEC seems to be a potent agent preventing translocation of enteric bacteria from the gut following major liver resection, probably by balancing enteric microflora, inhibiting bacterial attachment onto the intestinal surface and blocking phagocytosis by intestinal macrophages.

**F024**

### PANCREATIC INSULINOMAS: OUR PERSONAL EXPERIENCE

G.R. FRONDA, S. ENRICO, M.P. CAPOZZI, M.TOPPINO, U. CATTANEO
Department of Surgery. University of Torino (Italy)
(Head: Prof. F. Morino)

Pancreatic insulinomas represent the main cause of primitive hyperinsulinism. Since 1959 we have operated 36 patients for suspected insulin-secreting neo-plasms of the pancreas; 29 cases turned out to be insulinomas (2 multiple, 3 malignant) 5 diffuse insular hyerplasia; 2 hypoglycemic syndromes with no specific anatomo-phatological features. The preoperatory localization was obtained in 18 patients (50%); intraoperatory diagnosis was established in 79.9% of the cases, 96.5% in the 29 cases of true insulinoma. In 28 cases of true insulinomas, 18 corporo-caudal resections and 10 enucleations were carried out; the 3 malignant insulinomas were treated with extensive distal pancreatectomy; in 2 cases, hepatic metastasis were already present (palliative enucleation in one and resection in the other). For the neoplasms not localized, the intervention consisted on a programmed, monitored distal resection: we observed 1 case of occult insulinoma and 5 insular hyperplasia, while in 2 patients no insulinoma was found. The operative mortality was zero: postoperative complications, specific and aspecific, occurred in 10 pts. Long-term results (mean follow-up 9 yrs), in the 26 cases of benign insulinomas, included 22 complete resolutions and 4 improved clinical conditions. In the 3 cases of malignant lesions we observed residual hypoglycemia only in 2 patients that presented hepatic metastasis. The 7 cases of diffused hyperplasia had a decisive and definitive improvement, temporarily present in those two patients with a negative hystological exam. In these neoplasma, the relative diagnostic facility contrasts with the difficulty encountered in localizing the neoplasms. In the cases that concerned simple adenomas, the therapeutic possibilities depend on tumor site, reserving the resections for multiple localizations or uneasy enucleations. Resections is a must in the malignant forms. Unlocalized lesions, on the other hand, require a resection with simultaneous monitoring of the glycemic blood levels combined to a hystologic control.

**F025**

### VITAMIN A ABSORPTION, A NEW TEST FOR QUANTITATIVE EVALUATION OF EXOCRINE FUNCTION IN CHRONIC PANCREATITIS (CP)

I.P. HAJJ, M.C.A. PUNTIS, D. JAMES*
Departments of Surgery and *Biochemistry, University of Wales College of Medicine, Heath Park, CARDIFF CF4 4XN, South Wales, U.K.

There is a need for non invasive repeatable techniques to measure exocrine function in chronic pancreatitis.

Vitamin A esters are hydrolysed in the gut by a pancreatic lipase before absorption into mucosal cells. This phemomenon forms the basis of a new and simple test of pancreatic exocrine function.

A physiological but safe oral dose of 10,000 IU Vitamin A is given after a 12 hour fast. Pre-dose and them hourly blood samples are taken for retinyl, esters (RE), retinol binding protein (RBP), retinol and pre-albumin (PA). In 30 CP patients (diagnosed clinically and by ERCP), the mean RE level at 3 hours was 63 nmol/l compared with 296 nmol/l in a control population.

We think that this significant difference in absorption is due to inadequate enzyme secretion from the pancreas to hydrolyze the administered Vitamin A ester. We know that most of the patients had normal RBP levels (38 mg/lit) and normal liver function tests as well, consistent with normal protein synthesis.

The degree of Vitamin A absorption failure correlates with the severity of other features of CP. There are in addition, differences in the pattern of absorption between CP patients with alcoholic and non-alcoholic aetiology.

**F026**

### OPERATION FOR CHRONIC PANCREATITIS

M. ROKKJAER, J. JEPSEN, A. KRUSE, J. RAVNSBAEK
Surgical Department L, Aarhus University Hospital, Denmark

71 patients with chronic pancreatitis were operated 1982-1989 because of severe chronic pain: 54 men, aged 15-72 years, median 43, and 17 women, 30-76 years, median 37.The pain had lasted for 1/2-10 years, median 2 years, no difference between the three types of operations.

42 patients were operated with a Partington-Rochelle drainage procedure, 18 a.m. Whipple, and 11 patients with resection of body and tail. Type of operation was decided by ERCP: drainage was performed in diffuse disease with dilatation of the whole pancreatic duct to at least 6 mm; in severe changes without dilated duct resection was performed. Supplementary biliary surgery was done in 6 drainage operations and 2 resections.

No perioperative mortality. Median hospitalization time 14 days for all three types of operations. Two patients had to be re-operated because of complications 17 patients died before follow-up, in four cases death was related to pancreatitis.

77 patients (80%) answered a questionnaire after an observation time of $1^1/_2$ -$8^1/_2$ years, median $4^1/_2$ years : Visick 1: 47%, Visick 2: 11%, Visick 3: 21% Visick 4: 21%. 5 patients had diabetes preoperatively and another 20 at the time of follow-up. 35 patients had diarrhoea preoperatively, 8 of these had normal stools after drainage. 40% were unemployed and a further 30% after operation, worst in the Whipple group. In carefully selected patients with painful chronic pancreatitis operation will yield satisfactory results in 70-80%.

**F027**

### PANCREATIC MUCINOUS CYSTADENOMA AND CYSTADENOCARCNOMA. A MULTICENTER STUDY OF 51 CASES

G. ZEITOUN, J.M. HAY
French Association for Surgical research.
8, avenue des Peupliers, 92270 Bois-Colombes, France

Pancreatic mucinous cystadenoma (MCA) is a potentially malignant tumor which can change into pancreatic cystadeno-carcinoma (CAC). This retrospective multicenter study conducted by the French Association for Surgical Research from 1.1978 to 12.1987 was designed to compare the prognosis of these two pathologies.

MCA: all patients were females (mean age 44 yrs, ranging from 23 to 72). The tumor was localized in the head: 3, in the body: 5, and in the tail: 21 cases. Mean diameter of the tumor was 8 cm. Surgical procedures were as follows: 1 biopsy, 1 cystojejunostomy, 27 total resection of the tumor: 1 Whipple procedure, 24 distal pancreatectomies, 2 tumorectomies. No postoperative death occurred. All patients are alive, free of any recurrence at last follow-up.

CAC: There were 11 males and 11 females (mean age 63 yrs ranging from 49 to 82). The tumor was localized in the whole pancreas: 3, in the head: 12, in the body: 6, and in the tail: 1 case. Surgical procedures were as follows: 8 curative resecions, 4 palliative resections, 10 internal biliary and digestive bypasses. Two patients died postoperatively. Twelve patients died during follow-up with a mean survival of 10 months Eight patients are alive, but 4 with recurrence.

These results suggest that MCA of the pancreas should be systematically resected. This is justified by: 1/the potential risk of MCA to change into CAC; 2/the good results of surgical treatment of the MCA; 3/the poor prognosis of CAC even if radical surgery is performed.

**F028**

### INTRAOPERATIVE RAPID RADIOIMMUNOASSAY FOR INSULIN (IRRI) IN LOCALIZING INSULINOMA

K.H. ZHANG, Z.Y. GU
Dept. of Surgery, Chinese PLA General Hospital, Beijing, China

Abstract: In patients with organic hyper-insulinism, intraoperative rapid radioimmunoassay for insulin (IRRI) in veins draining the pancreas might help localize the tumor. Blood samples for IRRI are taken at various sites in the splenic and portal veins developed by us in a simple way. Of 13 patients, the highest insulin concentrations correlated with the site of the tumor in 12. The high insulin level is detected at the hepatic hilus in remaining one with diffuse insulopathy. Also, Small insulinomas less than 1 cm in diameter could be found. In one patient with a tumor of 0.4 cm in diameter and another one with two microinsulinoms are rationally resected by distal pancreatectomy, which could not be detected by any imaging procedure and palpation at operation. Therefore, it could be avoid blind distal resection of the pancreas, and possibly invasive preoperative diagnostic procedures.

**F029**

### ADJUVANT INTRAOPERATIVE RADIATION THERAPY VERSUS RESECTION ALONE IN THE TREATMENT OF PANCREATIC CANCER

A. ZERBI, D. PAROLINI, M. CARLUCCI, V FOSSATI*, V. DI CARLO
Pat. Chirurgica, Radioth.*-IRCCS S. Raffaele-Univ. Milan-Italy

We report our experience on the association of surgical resection with intraoperative radiation therapy (IORT) in the treatment of pancreatic cancer.

Between 1985 and 1991, 61 resections for pancreatic cancer, out of 192 observed cases, were performed at our institution. In 25 patients IORT was added to resection (group 1), whereas other 36 patients underwent resection alone (group 2) because of unavailability of linear accelerator or patient's refusal. Radiation doses from 15 to 20 Gy, with electron beam energies between 6 and 12 Mev, were delivered. Extension of the disease was analyzed: the mean diameter of the tumor was 3.1 cm in group 1, 3.5 cm in group 2; the percentage of nodal involvement resulted 62% in group 1, 43% in group 2. Operative mortality and overall post-operative complications were respectively 4% and 32% in group 1, 2.7% and 25% in group 2; no directly IORT-related complications were observed. One-year, two-year and three-year survival rate according to life-table method were respectively 62%, 27% and 22% in group 1; 40%, 18% and 11% in group 2. Median disease-free survival resulted 9 months in group 1, 7 months in group 2. Among patients with evidence of recurrent disease, a local recurrence was detected in 3 out 10 patients in group 1 (30%), and in 10 out 15 patients in group 2 (66%).

Our results suggest that a better local control and an improved survival could be obtained in patients with pancreatic cancer by adjuvant IORT.

**F030**

### INFLUENCE OF CLINICO-PATHOLOGICAL VARIABLES ON SURVIVAL AFTER PANCREATICODUODENECTOMY.

P JAGANNATH, L.J. DESOUZA, G. SINGH, V.M. MOHANDAS, V. SHANTISWAROOP
Tata Memorial Hospital, Parel, Bombay, India-400 012

Pancreaticoduodenectomy is a standard surgical procedure for adenocarcinomas involving the ampulla and head of pancreas. The significance of clinicopathological factors is controversial. Fifty seven patients underwent pancreaticoduodenectomy in a specialised cancer centre during 1985-90. A retrospective analysis of clinicopathological variables was undertaken. Majority (n=47) were ampullary carcinomas and head pancreas lesions formed only 17% which reflects the low resectability rate. 75% had moderately differentiated adenocarcinoma. The size of the tumour was measured on fresh resected specimens. The pathological variables were subjected to univariate analysis (SPSS). Actuarial survival was calculated using Kaplan-Mier life table analysis. 31.7% had positive peripancreatic nodes. There was no significant difference in survival of node +ve and node -ve patients. However size of the primary tumour had significance influence on overall survival. Median survival (MS) of tumours less than 1cm in size was 4 years. 1-2cms lesions had 2 yr 7m (MS) and tumours more than 2 cms had dismal survival of 7 months. Node positivity did not correlate with depth of duodenal infiltration in an ampullary lesion.

Mortality has decreased to 4.7% with a pancreatic leak rate of 2.4%. We conclude that though pancreaticoduodenectomy is a safe procedure in a specialised centre, the indication should perhaps be limited to tumours less than 2 cms.

**F031**

### VALIDITY OF COMBINED RESECTION OF THE PANCREAS AND PORTAL VEIN FOR CARCINOMA OF THE PANCREAS

S. TAKAHASHI, Y. OGATA, D. MAEDA, S. MURAI, K. YAMATAKA, T. TSUZUKI
Department of Surgery, Keio University School of Medicine, Tokyo, Japan

It seems to be prevalent opinion that patients with pancreatic cancer have a poor prognosis as a result of low resectability rate and extremely low long term survival rate. We have improved the result by performing combined resection of the pancreas and portal vein.

Between March 1976 and September 1991, 236 patients with pancreatic cancer were admitted to our clinic and 138 underwent resection. Seventy-seven 56% of the 138 patients received such procedure. As a result of this policy, resectability increased to 58%. Pancreatoduodenectomy and total pancreatectomy were carried out in 45 and 32 patients, respectively. According to pTNM classification, 11 patients were in stage I, 12 in stage II, 40 in stage III, and 14 in stage IV. Curative resection was performed in 39 (51%) of the 77 patients.

Thirteen patients (17%) died postoperatively and the remaining 64 were discharged from the hospital. The mean survival periods were 24 months in the curative resection group and seven months in the non-curative resection group, a statistically significant difference. Four patients undergoing curative resection lived more than five years and the longest survivor is alive well eight years and three months.

Combined resection of the pancreas and portal vein is a worthwhile procedure to improve the treatment for pancreatic cancer.

**F032**

### EXCHANGE EFFECT OF PARTIAL GASTRECTOMY ON PANCREATIC CARCINOGENESIS

P WATANAPA, B FLAKS, H OZTAS, PH DEPREZ, J CALAM,RCN WILLIAMSON
Department of Surgery and Medicine, Hammersmith Hosp., RPMS, Du

Cane Road, London W12 ONN, United Kingdom

The controversial issue of enhanced pancreatic cancer risk following partial gastrectomy has been explored in male Wistar rats (n=40) weighing 250-300g Animals were randomised to receive either 60% distal gastrectomy with Roux-en-Y reconstruction or gastrotomy and resuture (control). Immediately after operation each group was further divided into subgroups, receiving i.p. injections of saline or azaserine (30mg/kg/wk for 3 wks). At 15 mo blood was obtained at 0,5,15 and 30 minutes after a fatty meal for cholecystokinin (CCK) assay; rats were then killed. Pancreatic wet weight was measured, and histological sections were examined for atypical acinar cell foci (AACF), the putative precursor lesion of carcinoma. There were no significant differences in body weight or pancreatic weight between controls and rats with gastrectomy. Only azaserine-treated rats had acidophilic AACF. Partial gastrectomy substantially increased the number of acidophilic AACF per pancreas (median 26.05 vs 2.09,$P<0.005$), with a 9-fold increase their volume ($p<0.005$). Basal and postprandial plasma CCK concentrations were higher after gastrectomy than in controls ($P<0.05$). Partial gastrectomy has an enhancing effect on azaserine-induced pancreatic carcinogenesis, probably by means of increased CCK release.

### F033

### NEOADJUVANT CHEMORADIATION FOR LOCALLY ADVANCED PANCREATIC AND AMPULLARY CARCINOMA

RS YEUNG, JL WEESE, AR PAUL, JP HOFFMAN, LJ SOLIN, PF ENGSTROM and MJ KOWALYSHYN
Fox Chase Cancer Center and Presbyterian Cancer Center, Philadelphia, Pa. USA

A prospective study involving preoperative chemoradiation (CRT) for locally advanced pancreatic and periampullary carcinoma was evaluated. Thirty patients (14 females, 16 males;ages 37-79) with biopsy proven adenocarcinoma of greater than 4 cms involving the pancreas (25 head, 2 body/tail) and duodenum (3) received radiation (5040 cGy, 180 cGy/fx, 5 days/week) concurrent with 5-FU, 1 gm/m$^2$/day continuous infusion (days 2-5, 28-32) and mitomycin C 10mg/m$^2$ bolus (day 2). One patient died of biliary sepsis prior to completion of CRT. On restaging, 4 had metastases, 2 had portal vein occlusion, 1 had poor performance status and 1 refused operation. The remainder underwent laparotomy 3-6 weeks after CRT. Fourteen pancreatectomies (10 total, 2 Whipple, 2 distal) were performed with two in-hospital deaths. Resectability rate 44% (12/27) for pancreatic and 67% (2/3) for duodenal carcinoma. All specimens revealed clear margins and extensive necrosis with hyalinization. Lymph node metastasis were found in 14%. The two duodenal carcinomas had no residual tumor. The actuarial 5 year survival was 43% and 0% for the resected and non-resected groups respectively (p=.04). Considering pancreatic carcinoma alone, the actuarial three year survival was 35% for the resected group and 0% for non-resected group (p=.07). Pattern of failure included 1/14 locoregional, 2/14 hepatic and 1/14 pulmonary. Neoadjuvant therapy can be given safely and allows for complete resection in 67% of those who come to laparotomy.

### F034

### DIAGNOSIS OF PANCREATIC CANCER BY DETECTION OF CANCER-ASSOCIATED ANTIGENS IN SERUM WITH MONOCLONAL ANTIBODIES

M Yuan
China Great Wall Hospital, Beijing China.

A panel of monoclonal antibodies were derived from BALB/c mouse immunized with mucin of pancreatic cancer cell line SW1990.four of them designed as PS-1, PS-2, PS-7 and PS-10, all secreated IgM. When tested in paraffin sections of malignant and normal human tissues by SABC immunohistochemical methods, 90% of pancreatic cancer tissues reacted with PS-1, PS-2, and PS-10, while 70% reacted with PS-7. Most of gastric cancer, colorectal cancer and Vater's ampulla cancer reacted with these four antibodies, but less in other cancers. Normal pancreatic tissue reacted with PS-1, PS-2 and PS-10 differently, but not with PS-7. The related antigens of four antibodies are highly glycosylated molecules (200KD) with mucin-like biochemical characteristics. They are all gastrointestinal cancer-associated antigens.

The detection of cancer-associated antigens in sera is based on the inhibition of binding of antibodies to target antigens. The positive rate was determined by the ELISA when index of binding inhibition greater than the mean ±2SD of normal individuals. In pancreatic cancer patients, 58.8% , 62.5%, 75.8% and 87.5% were positive with PS-1, PS-2, PS-7 and PS-10. While in normal individuals, 4%, 4%, 3%, and 6% were positive. When these four monoclonal antibodies were used in combination (cock-tail), the positive rate was increased to 96.2% in pancreatic cancer and only 7% in normal individuals and 15.2% in non-malignant diseases. These study show the potential usefulness of monoclonal antibody in the diagnosis of human pancreatic cancer.

### F035

### FACTORS INVOLVED WITH LOCAL SPREAD, REGIONAL LYMPH NODE METASTASIS AND SURVIVAL IN RESECTED PANCREATIC CANCER

S.T. BROWER MD, R. NEWMAN MD, D. PERTSEMLIDIS MD, I. KREEL MD, A.H. AUFSES, JR MD
Department of Surgery, Mount Sinai School of Medicine, 1 Gustave L. Levy Place, New York, New York 10029.

We have examined the histopathological factors affecting the degree of local spread, regional lymph node (RLN) metastases, and overall survival (O.S.) in a group of 50 cases of resected carcinoma of the exocrine pancreas. Although the mean O.S. for the group was 14.3 months, resected patients without RLN involvement had a mean survival of 24 months. In contrast the mean O.S. rate was 8 months for patients with RLN involved. Size, tumor location, and histological grade were compared to RLN involvement and O.S. The mean size of primary tumor did not differ significantly between patients with or without RLNs (5.1 vs 4.6cms). However, 7 of 8 T1 tumors were <4cm and 35% of tumors <4cm were T1 lesions. In contrast, only 1 of 17 (6%) of tumors >4cm was T1. Histological grade was correlated with nodal status and O.S. There was a significant difference between histological grade and the presence of metastatic lymph nodes (G1, 37% positive, G2-4 55% positive). Patients with well differentiated tumors had a mean survival of 21 months compared to a mean survival of 10 months for less differentiated tumors (p<0.05). This difference was even more significant when stratified for nodal status. The patients with well differentiated tumors and no RLN involvement had a mean survival of 36.1 months compared to 8.6 months for well differentiated tumors with RLN involvement. In summary, we have shown that size, histological grade, and local spread predict for nodal status. However, specific patient subsets (G1, node negative) exhibit an excellent survival when curative pancreas resection is successful.

### F036

### EXFOLIATIVE CYTOLOGY FOR DIAGNOSIS OF BILIARY STRICTURES

T. KURZAWINSKI, B. DAVIDSON, R. DICK, A. DEERY, J.S. DOOLEY AND K.E.F. HOBBS
Hepatobiliary & Liver Transplantation Unit, Cytology Dept, Royal Free Hospital and School of Medicine, London, UK.

Imaging of biliary strictures may suggest malignancy but bile (BIC) and brush (BRC) cytology can provide tissue diagnosis.

We studied 92 consecutive patients (median age 66 years, range 39-91) with biliary strictures on ERCP (71) or PTC (21). Brushings (38) were taken using a modified Geenan cytology brush (6 Fr, Wilson Cook) passed alongside a guide wire which remained through the stricture. Bile (92) was aspirated after internal/external catheter or endoprosthesis insertion. Specimens were examined by one experienced cytologist (AD) and reported as positive or negative for malignant cells. Malignant (66) or benign disease (14) was confirmed by histology or laparotomy in 52 patients and by follow up in 28. 12 patients with uncertain diagnosis were excluded. The overall sensitivity of BRC was significantly greater that BIC (59% vs 30%; p<0.01) as was diagnostic accuracy (69% vs 43%; p<0.01). Specificity was 100%. Sensitivity for each tumor was:

| | Pancreatic carcinoma | Cholangio carcinoma | Ampullary carcinoma | Other tumours |
|---|---|---|---|---|
| | (28) | (20) | (7) | (11) |
| BIC | 30% | 30% | 29% | 18% |
| BRC | 55% | 57% | 100% | |

There was no procedure related complication and the average sampling time was less than 5 min.

We conclude that BRC is more sensitive than BIC in detecting malignancy and with the technique described is safe and rapid.

### F037

### PERCUTANEOUS USE OF SELF EXPANDABLE METALLIC STENTS IN ADVANCED MALIGNANT HILAR LESIONS

J.S. LAMERIS, J. STOKER, H.W. TILANUS, M. VAN BLANKENSTEIN, O.T. TERPSTRA
Department of Radiology, Gastroenterology and Surgery, University Hospital Rotterdam, Dr. Molewaterplein 40, 3015 GD Rotterdam, The Netherlands.

Purpose: The value of self expandable stents (wallstent; Medinvent, Lausanne) in the palliative treatment of patients with malignant hilar obstruction was studied.

Methods: Fifty-one patients were treated by a total of 72 stents. Six patents had a type I lesion, 9 a type II lesion and 36 a type III or IV lesion. Stent diameter was 1 cm; length 3.5-10.5 cm.

Results: In all but 7 patients (type III or IV) bilirubin levels turned to normal values. The median survival of 32 patients who died was 4.3 months (0.7-18m). Nineteen patients are alive after a median period after stent placement of 10 months (1-28M). Recurrence or worsening of the jaundice was seen in 17 patients, after a median period of 8 months. Tumor overgrowth at the proximal end of the stent(s) was the cause in 7, at the distal end in 4, tumor ingrowth, haemobilia and angling of the stent each in one patient. The cause was not established in two patients. Re-intervention was performed in 15 patients (29%). Thirteen patients benefited from the re-interventions (median follow up 3 months).

Conclusion: The use of the Wallstent in malignant hilar biliary obstruction compares favourable to plastic stents, as re-obstruction is mostly not stent related, but caused by tumor progression.

### F038

### IMMUNOHISTOCHEMICAL EXPRESSION FOR EGF RECEPTOR AND c-erbB-2 ONCOGENE PRODUCT IN BILE DUCT CANCER

R. SASAKI, S. KANNO, T. SUTO, Y. TAMASAWA, H. TOYOSHIMA, H. OHMORI and K. SAITO

In order to reveal the cell-biological character of bile duct cancer, expression of epidermal growth factor receptor (EGFR) and c-erbB-2 oncogene product were studied immunohistochemically. Further, the relationships between those expressions and histopathological parameters or prognosis were investigated.

MATERIALS AND METHODS: Twenty-six cases of bile duct cancer were used. The avidin-biotin peroxidase complex (ABC) method using monoclonal antibodies for EGFR and c-erbB-2 product was performed.

RESULTS:

1) The expression for EGFR was observed in 7 of 26 cases (26.9%) and c-erbB-2 product was in 13 (50.0%).

2) EGFR expression was higher in the cases of papillary adenocarcinomas (41.7%) and well differentiated adenocarcinomas (40.0%), compared with in moderately (0%) or poorly (0%) differentiated adenocarcinomas. C-erbB-2 product was highly stained in the cases of long tumor length (44.4% less than 3cm, 62.5% more than 3 cm), and in advanced stage (stage 1 0%, 2 44.4%, 3 44.4%, 4 71.4%)

3) In EGFR positive cases (n=7) five year survival rate was 0%, on the other hand, in EGFR negative cases it was 63.6%.

CONSLUSIONS: These results suggest that the immunohistochemical expression for EGFR may be a prognostic factor in bile duct cancer.

### F039

### LONG-TERM SURVIVAL OF PATIENTS WITH CARCINOMA OF THE MAIN HEPATIC DUCT JUNCTION

Y. SUGIURA, S. NAKAMURA, S. IIDA, Y. HOSODA, S. IKEUCHI, T. TSUZUKI
Keio Bile Duct Cancer Study Group, 35 Shinanomachi, Shinjuku, Tokyo Japan

Long-term survival of patients with carcinoma of the main hepatic duct junction is now the issue. We herein report on 10 patients who lived more than five years after surgery. Surgery was carried out by surgeons in six institutions, who were trained at Keio University Hospital, under the same policy that extensive resection of the bile duct combined with liver resection be the procedure of choice. In a total of 140 patients, 79 underwent resection, a resectability rate of 56.4%, 7 patients died postoperatively, an operative mortality rate of 8.8%. Ten of the 79 patients lived more than five years after surgery. Procedures of liver resection in the 10 patients were right trisegmentectomy in four, right lobectomy in threeand left lobectomy in three.. Combined resection of the portal vein was carried out in three. The resected margins of the bile ducts were clear in all patients. Histologic diagnosis was well differentiated adenocarcinoma in five and moderately differentiated adenocarcinoma in four. Lymphatic metastases were observed in two. It should be stressed that extensive resection of the bile ducts combined with liver resection is feasible and valid.

### F040

### LIVER INVOLVEMENT IN GALLBLADDER CARCINOMA: A STUDY OF ITS MODES

Y. SHIRAI, K. YOSHIDA, K. TSUKADA, T. MUTO
Department of Surgery, Niigata University School of Medicine, Niigata, Japan.

The aim of this study is to clarify the modes of liver involvement in gallbladder carcinoma (GBC).

Patients and Methods: Eighty-five cases of GBC underwent a radical surgery. Twenty cases (24%) showed liver involvement around the gallbladder bed. The resected liver was examined histologically by a multiple section technique in the 20 cases.

Results: (1) The spread was classified into 3 types as follows: 1) direct extension type (4 cases), 2) lymphatic spread type (13 cases), and 3) hematogenous spread type (3 cases). The direct extension type was defined as an expansive tumor growth without any satellite lesion. The lymphatic spread type was defined as the tumor with marked lymphatic involvement in the portal apace around the tumor. Hematogenous spread type was defined as the lesion with satellite deposits of hematogenous origin. (2) Microscopic non-contiguous satellite lesions, which were not grossly identified, were found in all cases of hematogenous spread any lymphatic spread. Hematogenous spread type showed microscopic satellite lesions within 1 mm from gross tumor. Lymphatic spread type showed microscopic satellite lesions within 1 mm from gross tumor. Lymphatic spread type showed microscopic satellite lesions at a distance ranging from 0.8 to 11.5 mm. The distance (=Y mm) from the gross border of liver invasion to the farthest satellite lesion correlates to the gross depth (=X mm) of the liver invasion. The formula of the correlation was: $Y=0.34X+1.2$ ($p<0.01$).

Conclusions: (1) Lymphatic spread along the portal space was a predominant mode of liver involvement. (2) Surgical margin of liver resection should be decided in consideration of the possibility of non-contiguous satellite lesions.

### F041

### RESULTS OF A RANDOMIZED STUDY OF BOLUS VS. CONTINUOUS I.V. APPLICATION OF APROTININ FOR REDUCTION OF BLOOD PRODUCT REQUIREMENTS IN ORTHOTOPIC LIVER TRANSPLANTATION (OLT)

W.O. BECHSTEIN, G. HIMMELREICH, H. RIESS, M. MUSER, R. ROSSAINT, P. NEUHAUS
Depts. of Surgery, Internal Medicine, and Anesthesiology, University Clinic Rudolf Virchow, Berlin, Germany

A reduction of blood product requirements in OLT with systemic application of aprotinin has been reported. However, randomized studies are still lacking. Effects of different aprotinin regimes on hemostatic changes and blood product requirements in OLT were investigated in an open, prospective, randomized study. OLT was carried out according to standard techniques. Adult patients were randomized to receive either bolus treatment of aprotinin (3 x 0.5 mio. KIU (kallikrein inhibitor units) (n=13)(group=1) or continuous aprotinin infusion of 0.1–0.4 mio. KIU/h (n=10)(group 2). Signs of hyperfibrinolysis before and after graft reperfusion were significantly lower in group 2 as measured by whole blood clot lysis time in thrombelastography (TEG). Tissue plasminogen activator (t-PA) activity increased significantly less in group 2 than in group 1 during the anhepatic phase. Intraoperative blood product requirements were higher in group 1 (median RBC u. 8 [4–1] than in group 2 (median RBC u. 7 [4–13]) (n.s.) Transfusion requirements during the first 3 postoperative days were significantly lower in group 2 than in group 1 (median RBC u. 1.5 vs. 3.5 [p<0.05]). Analysis of the early perfusate of the liver graft detected signs of decreased t-PA release of the graft in group 2, pointing to a protective effect of high aprotinin concentrations to hepatic endothelial cells. The results demonstrate the advantage of continuous aprotinin infusion over bolus application in OLT.

### F042

### BILIARY TRACT COMPLICATIONS IN LIVER TRANSPLANATION

MORENO G.E; GARCIA G.I.; ALVARADO A.A.; LOINAZ S.C.; CGOMEZ S.R.; JIMENEZ R.C.; GONZALEZ-PINTO A.I.; BERCEDO M.J.; IBANEZ A.J.; MORENO S.C.
Digestive Surgery Department.
Hospital 12 de Octubre. Madrid. Spain

INTRODUCTION: Biliary tract reconstruction is considered the "Achille's heel" of liver transplantation. Many forms of reconstruction had been proposed to decrease morbidity (between 8 to 86% in literature reports).

MATERIAL AND METHODS: Between April 1986 to September 1991, 233 orthotopic liver transplants (OLT) have been performed at "12 de Octubre" Hospital (three intraoperative deaths), 188 in adults and 12 in children. Single layer suture of 5/0 poliglactic acid (Vycril) was the technical preference, 8 to 12 F size of T-tube was the more common. Biliary complications were diagnosed by biochemistry, radiology and scintigraphy.

RESULTS:

| Biliary Anastomosis | CD-CD T | CD-CD | H-J | TOTAL |
|---|---|---|---|---|
| Number of OLT | 125 | 52 | 50 | *230 |
| Total complications | 13(10.4%) | 6(11.5%) | 6(12%) | 25(18.8%) |

CD-CD T: Choledocho-choledochostomy with T-tube
CD-CD: Choledocho-choledochostomy without T-tube or stent
H-J: Hepatico-jejunostomy
* Three choledoco-choledocystojejunostomy without complications.

Complicatons include: biliary leakage (7); leakage after removal T-tube (2); Fistula (3); Obstruction (1); Stenosis (4); Stenosis + stones (2); Anastomotic dehiscence (2); Biloma and collection (4).

CD-CD is our first choice method of reconstruction T-tube has the advantage of direct radiological access. Not using it simplifies the technique, but may delay the diagnosis of complications.

### F043

### PROTEINASE INHIBITOR (APROTININ) FOR THE TREATMENT OF REPRFUSION INJURY AFTER LIVER TRANSPLANTATION

### AN EXPERIMENTAL STUDY IN PIGS

K.J. OLDHAFER, J. HAUSS, H.U. SPIEGEL, R. PICHLMAYR
Hannover Medical School, Department of Surgery, F.R.G.

Recent work had shown that cold preservation produced a sinusoidal lining cell injury in liver allografts and that the attachment mechanisms of sinusoidal lining cells to the extracellular matrix might be involved in this pathogenesis. The purpose of this study was to determine whether aprotinin given during the reperfusion may reduce this injury in a pig liver transplantation model.

METHODS: Pigs weighting 18-26 kg were used for orthotopic liver transplantation (OLT). Aprotinin was administered IV in 7 recipients: 20.000 KIU/kg body weight as a bolus and 7.500 KIU/kg body weight per hour as continuous infusion for 6 hours after reperfusion. The cold ischemic time was 5.9±0.5 h in the untreated (n=8) and 5.7±0.75 h in the aprotinin-treated group (n=7). Blood samples were drawn in the donor, in the recipient after starting the operation, 1 and 6 hours after reperfusion and on postoperative days 1,3 and 5. Liver injury was assessed from release of transaminases.

RESULTS: In the untreated group 5 out of 8 pigs and in the aprotinin-treated group all pigs survived. In both groups GOT levels increased postoperatively and reached maximal values on the first postoperative day. However, in the aprotinin-treated group the increase of GOT was significantly lower (430±172 U/l) than in untreated group (887±375 U/l)

CONCLUSION: The results indicate that inhibition of proteases by aprotinin after revascularization is able to reduce the reperfusion injury. Since the hepatic extracellular matrix is mainly composed of proteoglycans and glycoproteins which can be altered by proteinases, protection of sinusoidal cell detachment may represent one important pathway through which aprotinin exerts its protective action.

### F044

### BILIARY COMPLICATIONS AFTER LIVER TRANSPLANTATION

B. RINGE, K. KOHLHAW, A. SCHMIDT, M. GALANSKI, R. PICHLMAYR
Medizinische Hochschule Hannover, Hannover, F.R.G.

Biliary complications following liver transplantation have always contributed significantly to postoperative morbidity. Despite better management by interventional radiology and reduced mortality prevention of these complications by a safe surgical techniques seems to be one of the most important prerequisites.

Therefore, we analyzed retrospectively the incidence of biliary complications in relation to the type of biliary reconstruction. Out of 635 operations performed within 18 years only 538 primary transplants were evaluated, including 445 adults and 93 children. Major types of biliary anastomosis were choledochocholedochostomy in a side-to-side =CdCdss (n=300) or end-to-end = CdCdee (n=60) fashion, and choledochojejunostomy = Cdj (n=165). 13 patients had no biliary reconstruction.

The overall biliary complication rate necessitating surgical reinterventions was 16.6% (87/525). The relationship between type of anastomosis and kind of complications is shown in the following table:

| Complications | total no. | (%) | anastomosis | T-tube | others |
|---|---|---|---|---|---|
| CdCdss | 26/300 | (8.7) | 15 (15.0) | 9 (3.0) | 2 (0.7) |
| CdCdee | 18/60 | (30.0) | 8 (13.3) | 9 (15.0) | 1 (1.7 |
| Cdj | 43/165 | (26.1) | 23 (13.9) | 8 (4.8) | 12 (7.3) |

Apart from T-tube problems or especially bleeding from the Roux-en-Y loop in patients with CdJ more than half of all complications were related to the anastomosis: CdCdss had, by far, the lowest rate of only 5%, in pediatric recipients there was no complication at all. These data from a larger series confirm our previous experience that CdCdss is the safest type of biliary reconstruction after liver transplantation, and should be considered as the anastomosis of first choice.

### F045

### RESULTS OF LIVER TRANSPLANTATION IN FULMINANT HEPATIC FAILURE AND LATE ONSET HEPATIC FAILURE IN CHILDREN

C.K. YEUNG, R. MONDRAGON, G.M. VERGANI, N. HEATON, A.P. MOWAT, B. PORTMANN, R. WILLIAMS, K.C. TAN.
Department of Surgery, Paediatrics, and the Institute of Liver Studies. King's College Hospital, London, U.K.

The mortality rate of fulminant hepatic failure (FHF) and late onset hepatic failure (LOHF) in childhood has remained between 70–95% despite recent improvements in medical therapy. Liver transplantation has become an important therapeutic option in adults with this entity, but has been performed very infrequently in children.

Between March 1988 and October 1991, 13 children aged from 1 month to 14 years with FHF or LOHF received a total of 14 liver transplants. This is the largest series reported in the literature. The aetiology was viral hepatitis in 5, undetermined in 4, drug hepatotoxicity (carbamazepine) in 2, autoimmune hepatitis and congenital haemochromatosis in 1. Reduced-sized livers were used in 11 of the 14 transplants. The postoperative morbidity included infective complications and abdominal bleeding. Two patients died from Graft Versus Host Disease, one from brain aspergillosis and another from graft infarction after portal vein thrombosis. Nine patients (70%) survive with a median follow-up of 18 months.

Liver transplantation should be the therapeutic option in children with FHF and LOHF where the chance of recovery with intensive medical therapy is poor.

### F046

### ORTHOTOPIC VS HETEROTOPIC LIVER TRANSPLANTATION IN THE PIG: EFFECTS OF LONG-TERM GRAFT PRESERVATION AND PROSTAGLANDIN $E_1$ ON INTRAOPERATIVE HEMODYNAMIC CHANGES

JD. BLANKENSTEIJN, P.M. SCHLEJEN, T.H.N. GROENLAND, O.T. TERPSTRA
Departments of Surgery and Anesthesiology, University Hospital Dijkzigt and the Laboratory for Surgical Research, Erasmus University, Dr. Molewaterplein 40, 3015 GD Rotterdam, The Netherlands

We compared the intraoperative hemodynamic changes during orthotopic liver transplantation (OLT) with those during heterotopic liver transplantation (HLT), after different duration of cold storage of the graft. The effect of prostaglandin $E_1$ (PGE)$_1$ on these parameters was also studied. Sixty-nine female Yorkshire pigs underwent wither OLT (N=32) or HLT (N=37) with a graft stored for 2 hr (N=31), 24 hr (N=16), 48 hr (N=7), or 72 hr (N=15) in the University of Wisconsin solution. PGE1 was added to the preservation solution and both donor and recipient animals in 16 transplantations of the various groups. Amongst others, cardiac output (CO), mean arterial pressure (MAP), and systemic and pulmonary vascular resistance (SVR, PVR) were measured at different time points during the operative procedure. For the three main variables - i.e., the type of transplantation, the use of $PGE_1$, and the preservation time, multiple regression analysis was performed. During HLT, portal vein clamping lowered MAP and CO, while during the anhepatic phase in OLT, SVR increased and CO dropped. After reperfusion of the graft an increase in PVR and a decrease in SVR was found in both OLT and APLT. At different stages of the surgical procedure, longer graft storage time diminished CO and MAP ($P<0.001$), especially in OLT. $PGE_1$ appeared to reduce the cardiovascular reserves needed to compensate the changes after recirculation of the graft. We demonstrated that extension of the graft preservation period resulted in poor cardiac performance, more so in OLT than HLT. The native liver in HLT might be able to metabolize the myocardial depressant factors, released into the circulation upon reperfusion. Prostaglandin $E_1$ did not protect against the reperfusion syndrome.

### F047

### SURGERY FOR BILIARY ATRESIA - THE PLACE OF LIVER TRANSPLANTATION

PJ FRIEND, G NOBLE-JAMIESON, S REVELL, RY CALNE, NV JAMIESON, PS JOHNSTON, ND BARNES.
The Departments of Surgery and Paediatrics, Addenbrooke's Hospital, Hills Road, Cambridge CB2 2QQ, UK

Although the benefits of porto-enterostomy in the primary treatment of biliary atresia are widely accepted, many patients obtain limited benefit and develop life threatening complications of chronic liver failure. The Cambridge-King's College Hospital liver transplant programme has carried out paediatric transplants since January 1984. We have reviewed the results of this procedure in patients with biliary atresia. The 73 such patients transplanted up to September 1991 have been analysed in 2 periods - 1984-88 (34 patients) and 1989-91 (39 patients).

The indications for transplantation were the development of life-threatening complications, particularly gastro-intestinal bleeding, ascites, recurrent sepsis, encephalopathy and cessation of growth.

In the earlier group 34 patients received 44 grafts (retransplant rate 29%). Graft survival at 1 and 2 years was 46% and 39%. Patient survival was 56% and 50%. In the later period 39 patients received 48 grafts (retransplant rate 21%). Graft survival at both 1 and 2 years was 66% and patients survival 84%. No graft or patient was lost after 2 years in either group.

The mean age of patients was 3.4 years (1984-8) and 4.6 years (1989-91). However, 10 (29%) of the early group were aged less than 2 years compared to 17 (44%) of the later group. More patients in the later series were transplanted with "reduced size" grafts (33% vs 7%) these being used in emergency cases, as a consequence of which very few patients now die whilst awaiting transplantation.

The results of paediatric liver transplantation have improved rapidly in recent years. We feel that this procedure should now be considered at an early stage in those cases where conventional surgery has proved unsuccessful.

### F048

### OPTIMAL TREATMENT OF BUDD-CHIARI SYNDROME (BCS) - CONVENTIONAL SURGERY OR LIVER TRANSPLANTATION?

B. RINGE, K. OLDHAFER, H. LANG, J. LAAS, H. LINK, R. PICHLMAYR
Medizinische Hochschule Hannover, Hannover, F.R.G.

BCS delineates a heterogenous group of various etiologic and morphologic disorders leading to hepatic venous outflow obstruction. Accordingly, the optimal treatment of this disease remains a controversial issue.

In order to elucidate the role of different operations we analyzed our own experience within the past ten years. This consecutive series includes 41 patients (30 females, 11 males) with ages ranging from 12-49 years. The etiology of BCS was known in approximately 50%; one patient had a veno-occlusive disease following bone marrow transplantation. 10 patients underwent conventional surgery as primary therapy: peritoneovenous shunt (2), caval thrombectomy and Maass stent (1), portocaval side-side shunt (2), mesocaval shunt (2), cavomesoatrial shunt (1), and dorsocranial liver resection and hepatoatrial anastomosis (2). Total hepatectomy and liver transplantation (LTx) was performed in 31 cases as first option; 4 additional patients were transplanted after failed previous surgery. So far, 15/41 patients have died. Only one of the primary non-transplanted patients has survived; 3/4 patients out of this group could be rescued by secondary LTx. 22/31 primary liver recipients are alive with a maximum follow-up of nine years (5-year actuarial survival 68%).

These results demonstrate clearly that LTx is one of the therapeutic options to be considered for patients with BCS. Certainly, our own experience is biased by the unfavourable outcome after conventional surgery thus supporting the more aggressive attitude of LTx. Even more, proper patient selection remains the crucial question which can only be answered by better understanding and definition of the pathogenetic, morphologic and clinical spectrum of this particular disease.

### F049

### AN IN VITRO METHOD FOR COMPARING THE EFFICACY OF TWO PRESERVATION SOLUTIONS IN ONE CANINE LIVER USING THE 5'-NUCLEOTIDASE ASSAY

T.M. VAN GULIK, C.R. NIO, W.M. FREDERIKS, J. RINGERS, L. Th. DE WIT, P.C.M. VERBEEK, P.J KLOPPER, M.N. VAN DER HEYDE.
Dept. of Surgery, Academic Medical Center, 9 Meibergdreef, 1105 AZ Amsterdam, The Netherlands.

The value of 5'-nucleotidase (5'-NT) as a marker of liver graft viability was studied in relation with liver preservation experiments. In 6 mongrel dogs, the main right and left branch of the portal vein were cannulated and flushed separately in situ with cold UW-solution (UW) and EuroCollins (EC), respectively. After hepatectomy, the right and left liver lobes were split and stored at 4°C in either solution. 5'-NT in liver tissue was determined histochemically (lead salt method) in cryostat sections. After 48h storage in EC, the 5'-NT score had decreased to 31%+16% (n=6), whereas in UW, 5'-NT score was 76%+10% (n=6). Significantly (p<0.05) higher 5'-NT scores were found also after 24h and 72h preservation time in UW. This result is in keeping with the higher preservation tolerance of liver grafts preserved in UW. The 5'-NT assay was applied in relation with graft function in orthotopic liver transplantation experiments in dogs. All dogs with liver grafts preserved in UW-solution during 24h (n=4) and 48h (n=3) survived (>5 days). Pre-transplant 5'-NT scores ranged from 61% to 100%. The 72h preserved livers (n=5) did not show life supporting function. Pre-transplant 5'-NT scores (33%+12%, n=5) were significantly (p<0.05) decreased. In conclusion, the 5'-NT assay in conjunction with the double flush method through the portal vein, provides a simple and rapid in vitro method to test solutions for liver preservation.

### F050

### LIVER GRAFT ASSESSMENT IN ORGAN DONORS BY THE LIDOCAINE/MEGX TEST IS UNRELIABLE.

R. REDING, A. FEYAERTS, P. WALLEMACQ, L. LAMBOTTE, J.B. OTTE.
Dept of Surgery, St-Luc University Clinics, Brussels, Belgium

Lidocaine/monoethylglycinexylidide (MEGX) test was performed in 38 liver donors prior to organ harvesting. The serum MEGX 15 min after an IV bolus of 1mg/kg of lidocaine in the donor (MEGX t15) was retrospectively correlated with outcome of the transplant and early liver graft function. Three donors were excluded from the study because of pre-test contamination with lidocaine. Among the remaining 35 liver transplants (OLT), 4 patients were retransplanted within 10 days post-OLT (primary nonfunction (PNF): n=4; early severe graft dysfunction: n=2), and three recipients died, with median (range) donor MEGX t15 (ng/ml) at 100 (86-119) and 169 (146-182), respectively, when compared to 87 (18-245) in the remaining 28 OLT patients alive with functioning grafts. Considering the literature (1), a MEGX t15 value < or >80ng/ml was considered arbitrarily as the cut-off point which may predict poor or good liver graft quality, respectively. Donor and preservation data were not statistically different in the 2 corresponding groups in terms of mean donor age (21.9 vs 21.0), days in intensive care unit (2.8 vs 2.6), ischemic time in hours (13.6 vs 12.7) and T-cell crossmatch. One month graft survival rate was significantly higher in the <80ng/ml group (12/12: 100%) when compared to the >80ng/ml group (7/23: 70%; p=.036), Moreover, early (days 1-5) post-OLT cytolytic and functional parameters were similar in both < or >80ng/ml MEGX t15 groups: peak SGOT (UI/1)(mean+SD: 2356+2622 vs 2173+3036, NS), peak SGPT (UI/1)(1684+1476 vs 1952+1939, NS), mean SGPT (1051+791 vs 1354+1533, NS), minimum PT (%) (43+17 vs 43+13, NS), and peak total bilirubin (mg/dl) (9.8+4.9 vs 10.6+5.0, NS). In conclusion, (1) the MEGX test failed to predict graft outcome, as the 2 PNF cases had "excellent" MEGX t15; (2) low donor MEGX t15 correlated neither with poor graft outcome nor with early liver dysfunction. Low MEGX t15 value should thus not preclude the use of a liver donor, particularly in the current context of organ shortage.

(1) Adam R, et al. Transplant Proc 23: 2470, 1991

### F051

### MODIFICATION OF RETICULOENDOTHELIAL FUNCTION BY MURAMYL DIPEPTIDE ENCAPSULATED LIPOSOMES IN JAUNDICED RATS TREATED WITH BILIARY DECOMPRESSION

R. ADERSSON, J.W. DING, B. HULTBERG*, S. BENGMARK
Departments of Surgery and Clinical Chemistry*, Lund, Sweden

Septic complications and renal dysfynction are major causes of morbidity and mortality following biliary surgery in the jaundiced patient. In previous studies we demonstrated that reticuleondothelial system (RES) function was severely depressed in rats with biliary obstruction and RES function tended to normalize very slowly following biliary decompression. Ways to increase the recovery of RES function following biliary obstruction are thus sought for. The present study investigates the influence of liposome encapsulated muramyl dipeptide (MDP) administered in jaundiced and biliary decompressed animals.

Rats with 2 weeks biliary obstruction, with or without 1 week of concomitant biliary decompression relieving the jaundice, were treated with either normal saline, free MDP, placebo liposomes or liposome encapsulated MDP. RES function was evaluated by the blood clearance (described as the corrected phagocytic index) and organ localization (liver, spleen, lungs, kidneys) of i.v. injected $^{125}$I-labeled, heat-killed *E. coli*.

The corrected phagocytic index following 1 week of biliary decompression returned to normal levels in animals treated with MDP-liposomes, while RES function was impaired in all other jaundiced and biliary decompressed groups. In the biliary decompressed, MDP-liposome treated group, renal localization of radiolabeled *E.coli* was significantly lower as compared to all other jaundiced biliary decompressed groups.

We conclude that treatment with MDP-liposomes improves the otherwise impaired RES function in rats with biliary obstruction and especially in the early phase following biliary decompression of jaundice.

### F052

### CAN WE DO AWAY WITH PTCD?

R.D. BAPAT, N.N. REGE AND S.A. DAHANUKAR
Department of Gastro-enterology Surgery and Department of Pharmacology, Seth G.S. Medical College & K.E.M. Hospital, Parel, Bombay 400 012, India.

Percutaneous transhepatic cholangiography and drainage (PTCD) is performed in surgical jaundice to decompress the biliary tree and improve hepatic functions. However, the risk of sepsis is high in these patients due to immunosuppression (1) and surgical outcome remains poor. This raises a question - can we do away with PTCD? To answer this query a study was carried out in 2 phases. During the 1st phase mortality was compared between 2 groups of patients: (A) those undergoing surgery without PTCD (n=11) and (B) those undergoing surgery following PTCD (n=13). The mortality was 57.14% in Gp A as compared to 61.54% in Gp B. Serial estimations of bilirubin levels carried out during the course of drainage (3 wks) revealed a gradual and significant decrease, from 12.52 ± 8.3 mg% to 6.9 ± 3.4 mg%. Antipyrine half-life did not change significantly (19.14 ± 3.7 hrs) compared to basal values (21.96 ± 3.8 hrs). The phagocytic and intracellular capacities of PMN remained suppressed (Basal: 22.13 ± 3.68% phago. and 19.1 ± 4.49% ICK; post drainage: 20 ± 8.48% phago. and 11.15 ± 3.05% ICK). Thus PTCD did not improve metabolic capacity of the liver and mortality was higher due to sepsis. Considering this high incidence of sepsis and based on our earlier studies which have demonstrated immunotherapeutic potential of Tinospora cordifolia (TC) (2,3), 2 additional groups were studied: (C) patients receiving TC during PTCD (n=16) and (D) patients receiving TC without PTCD (n=14). A significant improvement in PMN functions occured by 3 weeks in both groups (31.25 ± 4.65% phago and 24.8 ± 5.6% ICK). The mortality in Gp C & D was 25% and 14.2% respectively during preoperative period. There was no mortality after surgery. It appears from this study that host defences as reflected by PMN functions play an important role in influencing prognosis. Further decompression of the billiary tree by PTCD seems unwarranted.

(1) Rege et al, Ind J Med Res 1989: 90,478

(2) Dahanukar et al, HPB Surg 1990: 2 (Suppl), 38

(3) Bapat et al, HPB Surg 1990: 2 (Suppl), 210.

### F053

### ENDOPROSTHESIS RELATED BILE DUCT PATHOLOGY AN EXPERIMENTAL STUDY IN THE DOG

T. KARSTEN, P. DAVIDS, T.v. GULK, A. BOSMA, P.J. KLOPPER and M.N. v.d. HEYDE
Depts. of Surgery, Gastroenterology and Pathology AMC, Amsterdam, The Netherlands

The histopathological changes of extrahepatic bile ducts after a period of Endoprosthetic Biliary Drainage (ED) and its consequences for subsequent Biliary Tract Surgery were assessed in 21 mongrel dogs. 5 dogs (group A) underwent ED during 4 weeks and were sacrificed 2 months after removal (RS) of the stent. In six dogs (group B) the 4 weeks of ED was followed by hepaticojejunostomy (HJ) and observed for 2 months. In 5 dogs (group C) the common bile duct (CBD) was ligated (4 days) and treated subsequently with ED (4 wks) and HJ (2mths). In group D (n=5) the effect of CBD ligation (4 days) and direct HJ (2 mths) was studied. Stenting was accomplished by duodenotomy and CBD biopsies were obtained during HJ and upon sacrifice. Bile samples were cultured (BC) during each step of the procedure.

| N | 4 days | | 4 wks | | pos. bile culture after ED | 2 mths | | sacrifice ↓ p.o. complications infect | non-inf | pos.bile culture at sacirifice |
|---|---|---|---|---|---|---|---|---|---|---|
| A(5) | | – | ED ↔ | +++ | 100% | RS | + | 1x | no | 80% |
| B(6) | | – | ED ↔ | +++ | 100% | HJ ↔ | ++ | 3x | 1x | 100% |
| C(5) | cbd ↔ lig | + | ED ↔ | +++ | 100% | HJ ↔ | ++ | 2x | 1x | 100% |
| D(5) | | – | cbd ↔ lig | + | 30% | HJ ↔ | + | no | 1x | 100% |

▲ CBD inflammation (-,+,++or+++)

Conclusion: Endoprosthetic biliary drainage of both a normal and obstructed CBD resulted in a distended fibrosing bile duct, showing severe chronic inflammation with epithelial hyperplasia of the mucosa. At 2 months after removal of the stent the inflammation is still moderately present. A higher incidence of postoperative (infectious) complications was noted after biliary tract surgery with previous endoscopic stenting.

### F054

### A NEW THERAPEUTIC APPROCACHE TO TREATMENT OF HIGH RISK PATIENTS IN BILIARY SURGERY

ZH. SJ. ZHUMADILOV, D. R, MUSINOV, E. A, TAYGULOV
Semipalatinsk's Medical Institute, Kazakhstan

A new method of delivery of antibiotic to the biliary system by autological erythrocyte ghosts has been worked out experimentally and applied clinically in the patients w3ith acute complicated cholecystitis and high risk of urgent operation. We infused intravenously ghosts containing single dose of antibiotic only two times a day. Clinical trial consisted of two groups of patients. Group 1 - 67 patients who receivedd traditional conservative therapy and aminoglicosides by intravenously or intramuscularly route. Group 2 - 90 patients who received similar antibiotics by above stated special technique. No significant differences were noted among both groups as to sex, age and diagnosis. The results of treatment are listed in table.

| Patients | No. (%) of septic com plications of disease | No.(%) of urgent operations | Disappearence of clinical, laboratory symptoms of disease |
|---|---|---|---|
| Group 1 | 30 (44.8) | 20 (29.9) | 9.0 + 0.4 days |
| Group 2 | 0 | 0 | 1.9 + 0.01 days |

In patients of group 2, the amelioration immunological and clinical states was more marked. They were underwent an elective operation after detailed examination and preoperative management of multiple system disorders. The special pharmacokinetic investigations have shown a high concentration of antibiotic in the bile of patients (3 - 4.7 times) having new method versus patients who received intravenously, intramuscularly and even endolymphatic infusion of antibiotic. So, this new method is effective, available for clinical practice. It is also economical and be used in biliary surgery.

### F055

### CHANGE OF HEPATIC MITOCHONODRIAL REDOX STATE BY BILIARY DRAINAGE IN OBSTRUCTIVE JAUNDICE

T. TSUBONO, Y. SHIRAI, K. TSUKADA, K. YOSHIDA, T. MUTO, T. SHIMIZU
Department of Surgery, Niigata University School of Medicine, Niigata, Japan.
+Department of Surgery, Shinrakuen Hospital, Niigata, Japan.

Aim: The redox tolerance test (RTT) quantifies the changes of hepatic mitochondrial redox state by measuring the arterial ketone body ratio (KBR) in response to oral glucose load. The aim of this study is to clarify the liver function as measured by the RTT in obstructive jaundice (OJ) and to examine the changes in RTT during percutaneous transhepatic biliary drainage (PTBD).

Patients and Methods: 22 patients with OJ underwent RTT before and two weeks after PTBD. Patients with overt diabetes mellitus were excluded. RTT was performed as described by Mori et al (Ann Surg 1990; 211:438-446). A redox tolerance index (RTI=100XΔKBR/Δglucose), which means the response of arterial KBR to glucose loading, was used as an indicator for RTT. Results: The patients were classified into three groups according to RTI (Group A: RTI (before) >0.5; Group B: RTI(before)<0.5 and RTI (after)>0.5; Group C:RTI (before)<0.5 and RTI [after] 0.5). RTI were maintained above 0.5 in all patients in Group A (n=13) and no change was found after PTBD (before: 0.97±0.35 (mean±SD); after: 1.07±0.38). RTI in Group B (n=5) were improved variously after PTBD. There were no hospital deaths both in Group A and B. In Group C (n=4), RTI of 2 patients were fallen after PTBD and 3 patients died in the hospital by cholangitis. Conclusion: (1) RTT can be a useful method for the evaluation of outcome in OJ. (2) From the standpoint of hepatic mitochondrial redox state, PTBD has a limited role in improving liver function.

### F056

### EMBICA OFFICINALS: A NOVEL PHARMACOLOGICAL THERAPY FOR ACUTE PANCREATITIS

S.A. DAHANUKAR, S.P. THORAT AND R.D. BAPAT
Department of Pharmacology and Department of Gastroenterology Surgery, Seth G.S. Medical College and K.E.M. Hospital, Parel, Bombay 400 012, India

Mortality due to acute pancreatitis, is very high when it is complicated with secondary pulmonary damage. Management of pancreatitis is mainly supportive, and no pharmacological agent has yet been found useful. Based on its cytoprotective effect(1), Emblica officinalis (EO) was selected to investigate its ability to reduce pancreatitis and associated lung damage in dogs. This was compared with pentoxyfylline. 34 dogs of either sex were divided into 5 groups (n=6-8 each): Gp I-control, Gp II-acute pancreatitis, Gp III-sham operated, Gp IV-pretreatment for 15 days with EO 28mg/kg/day orally and Gp V-pretreatment with pentoxyfylline 50 mg/kg i.v. bolus followed by 0.1 mg/kg/min for 1/2 hr. Acute pancreatitis was induced by injection of a mixture of blood-bile-trypsin. Serum amylase, ABG, peripheral WBC counts, neutrophil function tests, and histopathology (HP) and electron microscopy (EM) of lungs and pancreas were studied basally and 2 hrs after inducing pancreatitis. In Gp II, pancreatitis was proven by a consistent rise in serum amylase (P<0.05) as well as by HP & EM. HP of lungs showed alveolar septal disruption and edema. EM showed the formation of large vacuoles with electron dense vacuoles. PMN functions were found to be stimulated. In dogs pretreated with EO, serum amylase did not rise significantly. Mild edema without necrosis or haemorrhage was seen on HP on pancreas. EM confirmed the normalcy of pancreas. Similarly lung damage was also prevented. However PMNS were still stimulated at 2 hrs suggesting that PMNs may not play a role in lung damage. Pentoxyfylline suppressed the PMNs as expected (P<0.05) but did not prevent lung damage, further proving this point. This study documents for the first time the efficacy of EO against pancreatitis and its pulmonary complication in dogs.

(1) Gogate VM, Ayurvedic Materia Medica, 1st Ed. Continental Prakashan, 1982, 247-248.

### F057

### THE IMPACT OF A NEW CLASSIFICATION ON THE TREATMENT OF PANCREATIC PSEUDOCYSTS

A.D'GIDIO, M SCHEIN, C.G. BREMNER
Department of Surgery, University of Witwatersrand, Johannesburg, South Africa

Our working classification consists of three types of cysts based on the two pathogenetic processes: autodigestion (Post-necrotic cysts) and ductal obstruction (Retention cysts).

94 patients with pseudocysts, divided in three groups: post-necrotic type I: in acute pancreatitis (50 patients). Post-necrotic type II: in chronic pancreatitis (32 cases). Retention or type III: in chronic pancreatitis (12 patients). E.R.C.P. performed in all patients with chronic pancreatitis in 11 patients with acute pancreatitis.

Average follow-up: 10.2 months (range 1-82).

Type I: percutaneous drainage (PD) was successful and did not carry complications nor did lead to cyst recurrence. Internal drainage PD should be the treatment of choice.

Type II: PD was associated with prolonged drainage and risk of complications, when cyst-duct communication was present. Internal drainage was effective and carried low morbidity and mortality, therefore it should be used when ductal communication is present.

Type III: PD not used. Internal drainage not adequate, leading to cyst recurrence; the duct needs to be addressed by drainage or resection.

Reference:

A.D'Egidio, M. Schein Br J Surg 1991; 78:585-9

### F058

### THE PYLORIC PRESERVING PANCREATODUODENECTOMY IN CHRONIC PANCREATITIS

M. GAGNER, D. McANNENY, R.L. ROSSI, J.W. BRAASCH
Dept. of Surgery, Lahey Clinic, Burlington, Massachusetts.

Standard pancreatoduodenectomy (PD) has been used in the management of selected patients with chronic pancreatitis (CP). The pyloric preserving modification has been introduced to maintain gastric capacity and gastric emptying, decrease post-gastrectomy symptoms and perhaps decrease malabsorption. Forty-five patients with the median age of 47 years, who underwent a pylorus preserving pancreatoduodenectomy (PPPD) for CP since 1979 and have been followed for a median of 60 months (18 to 122) were reviewed. All had abdominal pain, 90% were taking narcotics, a history of ethanol abuse was present in over half of the cases, and 35% of patients had a mass in the head of the pancreas. There was one operative death (2%) and ten major complications (22%). The mean pre-operative pain score (0-5) of 4.5 improved to 1.3 (P<0.0001), the mean pre-operative weight of 134 pounds improved to 137. The 40% incidence of clinical pre-operative exocrine insufficiency was not changed by PPPD. Ten patients (22%) had pre-operative endocrine insufficiency; post-operatively, four patients (9%) had worsening of their endocrine insufficiency. Early gastric complications (stasis) developed in 4% of patients, while late gastric complications (gastric hemorrhages and/or ulcerations) were present in 13% of patients. Ninety percent of the patients felt their pain had improved with more than three-quarters of the patients not taking narcotics or using a much lower dose than before operation. PPPD in selected patients with CP improves the pain in the majority of patients, can be safely performed, and is a good alternative to standard PD.

### F059

### EVIDENCE FOR AUTOIMMUNITY IN CHRONIC PANCREATITIS

RP JALLEH, JA GILBERTSON, CS FOSTER & RCN WILLIAMSON
Departments of Surgery and Histopathology, RPMS, Hammersmith Hospital, London, United Kingdom.

The precise aetiopathogenesis of chronic pancreatitis is unclear. High numbers of T-lymphocytes in livers and pancreatic specimens suggest possible involvement of cell-mediated immune mechanisms. Aberrant expression of major histocompatibility complexes (MHC) is a prerequisite for organ-specific autoimmunity, and normal pancreatic epithelial cells do not express such complexes. Expression of MHC Class I (β2-microglobulin) and Class II (HLA-DR) determinants were investigated in 93 patients (64 males, 29 females, mean age=41 years) operated on for chronic pancreatitis. Aetiological agents were alcohol (63 patients), recurrent acute pancreatitis (12), congenital lesions (2) and unknown (16). Immunohistochemical staining of tissue sections involved standard immunoperoxidase techniques using specific antisera. No MHC expression was seen in 10 histologically normal pancreases (controls). β2-microglobulin expression in pancreatic epithelial cells was observed in 76 specimens (82%) while HLA-DR was present in 61(66%). Staining of both MHC determinants was confined to ductular and ductal epithelial cells was observed in 76 specimens (82%) while HLA-DR was present in 61(66%). Staining of both MHC determinants was confined to ductular and ductal epithelium with no staining of acinar cells: β2-microglobulin (ductules 63%, ducts 51%;NS) and HLA-DR (ductules 59%, ducts 53%;NS). Positive staining was not related to aetiological agent or age. This aberrant MHC expression, together with a T-cell infiltration, suggests a cell-mediated component to chronic pancreatitis.

### F060

### PROGNOSTIC VALUE OF CT SCAN IN SEVERE ACUTE PANCREATITIS: A PROSPECTIVE, MULTICENTRIC STUDY.

N. ROTMAN, D. PEZET, D. MATHIEU, D. CHERQUI, C. CHASTANG, B. CHEVRET, P.L. FAGNIEZ

Hôpital Henri Mondor, Créteil, et Hôpital Saint-Louis, Paris, France.

From October 1986 to January 1991, 234 patients with severe acute pancreatitis (SAP) have been entered in a prospective, multicentric study to evaluate the prognostic value of early CT scan. The criterion for inclusion was a first attack of pancreatitis requiring an admission in an ICU. An initial CT scan was performed within 48 hours of admission in 228 patients. Each CT scan was performed throughout the entire abdomen before and after intravenous injection of contrast medium. A form was filled for each CT scan to collect the following data: size of the pancreas, enhancement after contrast medium injection, visualization of the portal and splenic veins on angioscans, number and location of peripancreatic collections. Survival curves and curves of occurrence of a pancreatic abscess were constructed by the method of Kaplan-Meier and compare with the Log Rank test. The non visualization of the portal and splenic veins on angioscans was related to increase mortality and abscess formation (p<0.0001). Collections located in the lower parts of the right and left mesocolons, in the right posterior pararenal space were predictive of abscess formation and increased mortality rate (p<0.01). The non visualization of a part of the pancreatic parenchyma on angioscans did not influence the prognosis. Concerning the Ranson CT scan classification, grade E was significantly related to increased mortality and abscess formation when compared with the other grades (p<0.02).

### F061

### MANAGING THE COMMON DUCT STONE IN THE LAPAROSCOPIC CHOLECYSTECTOMY ERA

D.R. FLETCHER, R.M. JONES
University of Melbourne, Department of Surgery,
Austin Hospital, Melbourne, AUSTRALIA

At open cholecystectomy, operative cholangiography was performed to confirm suspected or identify unsuspected C.B.D. stones allowing concomitant treatment. Since laparoscopic cholecystectomy (L.C.), instead of operative cholangiography, reliance has been placed on E.R.C.P. to diagnose and treat by sphincterotomy (E.S) C.B.D. stones. This has led to an increased incidence of ERCP and sphincterotomy with possible complications. To determine if these stones could be managed surgically in the laparoscopic era, L.C. with routine op. cholangiography was attempted in 200 surgically unselected patients. In those with suspected C.B.D. stones (L.F.T., U/S, pancreatitis, jaundice), in 75%, the stones had passed by the time of surgery. In 7 of 10 patients with C.B.D. stones, the duct was cleared with a combination of cholangioscopic or image intensifier monitored trans cystic duct sphincter dilation and flushing or basket extraction. There were no complications in this small number. Three patients required post op. ERCP sphincterotomy (DRF).

We conclude laparoscopic exploration of C.B.D. at L.C. is possible provided routine op. cholangiography is used. ERCP use will be reduced if not used pre operatively to screen for and treat C.B.D. stones but reserved for post op. retained stones the incidence of which may be increased

### F062

### RECENT RESULTS OF ELECTIVE OPEN CHOLECYSTECTOMY IN A NORTH AMERICAN AND A EUROPEAN CENTER. COMPARISON OF COMPLICATIONS AND RISK FACTORS.

P-A. CLAVIEN, J.R. SANABRIA, G. MENTHA, F. BORST, L. BUHLER, B. ROCHE, R. CYWES, R. TSIBISHIRANI, A. ROHNER, S.M. STRASBERG
Hepatobiliary-Pancreatic Section of the Division of General Surgery, Mount Sinai Hospital, University of Toronto, Toronto, Canada.
Clinic of Digestive Surgery, Department of Surgery, University Hospital, Geneva, Switzerland.

Results of elective open cholecystectomy (OC) in 1252 patients treated in a North American and a European center just prior to introduction of laparoscopic cholecystectomy were examined using a recent standardized classification of complications. There were no deaths, but 12% and 14% of the patients developed complications in each center, respectively. About 6% of the patients developed grade I (minor) complications. Grade II complications (without residual disability) were noted in 6% and 8%, and grade III (with residual disability) in 0% and 0.3%, respectively. Using uni-and-multivariate analysis, individual risk factors for developing the different categories of complications were different between both centers, whereas two preoperative score systems, ASA and a simplified APACHE II, were found to predictive for all types of complications in both centers. Elective OC is a safe procedure and new therapeutic alternatives should aim at similar results. Generalization of risk factors identified in a particular center may be misleading since numerous local conditions may significantly affect risk factors for surgery. The data also demonstrate the advantages of a uniform way of reporting surgical complications, which should allow the performance of valuable comparisons.

### F063

### CHOLEDOCHODUODENAL FISTULA ASSOCIATED WITH CHOLELITHIASIS

P. DI GIORGIO, G. RIVELLINI, L. ESERCIZIO, B. DE LUCA
Endoscopy Unit, Vecchio Pellegrini Hospital-USL 44-Naples, Italy

Choledochoduodenal fistula (CF) situated on or around the longitudinal fold is most probably caused by the spontaneous passage of gallstones. It was very difficult to diagnose until the introduction of duodenal fiberoscopy. At our Endoscopy Unit, of the 796 patients with choledocholithiasis who were subjected to endoscopic cholangiography, spontaneous CF was diagnosed in 58 (7.3%). The group consisted of 39 female and 19 male patients with an age range from 47 to 84 years. CF was classified in two types: type I was present on the longitudinal fold (75%) type II was present at the duodenal mucosa adjacent the longitudinal fold (25%). Common bile duct calculi were present in 49 patients (84%). The incidence of jaundice at the time of endoscopic examination in patients with fistula was 6.9% compared with 73.6% in patients without fistula. The spontaneous disappearance of jaundice in choledocholithiasis is probably due to formation of CF. Sphincterotomy was successful in all patients with fistula and common bile duct calculi (84%).

### F064

### ROLE OF EXTRACORPOREAL SHOCK WAVE LITHOTRIPSY IN THE MANAGEMENT OF COMMON BILE DUCT STONES

T.K. MALIK, S. MADAN, S. RAWAT, R. MALIK.
Dept. of Surgery, M.A.M College, New Delhi.

We evaluated the role of extracorporeal shock wave lithotripsy (ESWL) in the management of common bile duct (CBD) stones. A total of 40 patients were taken up for the study. All the patients had endoscopic papillotomy done and biliary drainage was established in all by nasobiliary catheter. Number of shocks given ranged from 700 to 11000. No more than 4000 shocks were administered in each treatment session with a minimum time interval of 48hrs. between consecutive sessions. Most stones were crushed around 17.2 K.V. Stone fragmentation was achieved in 80% of cases, and stone clearance was obtained in 70%. Adjunctive non-surgical procedures were necessary for stone removal in 30% patients and 15% patients required surgery to clear the ducts. Mean hospital stay was 8 days. There was no mortality and complication rate was relatively low. Nine patients had skin bruising and ecchymosis, five patients had exacerbation of cholangitis. Three patients had biliary pain. It was therefore concluded that ESWL is a helpful and safe adjunct in treating difficult-to-reach or difficult-to-remove bile duct stones in the elderly or high risk patients.

### F065

### MANAGEMENT OF BILE DUCT STONES IN THE ERA OF LAPAROSCOPIC CHOLECYSTECTOMY

A. ANTHONY, T. WILSON, L. KOW, P. JEANS, J. TOOULI
Gastrointestinal Surgical Unit, Flinders Medical Centre, Adelaide, South Australia, Australia.

The development of laparoscopic cholecystectomy (LC) has led to new dilemmas in the management of common bile duct (CBD) stones. This study examined the approach to CBD stones in 207 consecutive patients submitted to LC.

CBD stones were suspected pre-operatively in 50 patients on criteria of abnormal liver function tests, dilated CBD on ultrasound or a prior history of biliary pancreatitis. 14/50 had pre-operative ERCP with CBD stones found in 9 and extracted by endoscopic sphincterotomy (ES) in 8/9. In 4/9, at subsequent LC and operative cholangiogram (OC), residual stones were present. 2 required open operation and exploration of the EBD(ECBD), 1 had a follow-up ERCP and stone extraction and 1 was followed clinically as the stone was felt small enough to pass through the previous ES. 36/50 went to primary LC. In 35/36 OC was successful and in 24/35 the duct was clear. In 10 of the remaining 11, duct clearance was attempted laparoscopically by relaxing the sphincter pharmacologically and flushing the CBD via the OC catheter. This approach was successful in 4/10. Of the remaining 7, 3 went to open ECBD and 4 had follow-up ERCP within 42 days of LC. In 2/4 the CBD stone had passed by the time of ERCP and the remaining 2 required ES and stone extraction. CBD stones were detected on OC in 6/159 patients without pre-operative suspicion of CBD stones. In 3/6 the stones were cleared primarily at LC. 2/6 required subsequent ERCP. In 1 of these the duct was clear and the other required ES and stone extraction. 1 patient has been left to clinical follow-up.

In conclusion, it is possible to deal with CBD stones by laparoscopic techniques in over 1/3 of patients at the time of initial LC. In the remainder, CBD stones can usually be cleared by subsequent ERCP and ES, but about 1/2 will actually have passed their CBD stone by the time of follow-up ERCP. In this setting pre-operative ERCP should probably be reserved for patients with clinical jaundice or cholangitis at presentation.

### F066

### EFFECT OF SURGICAL MARGIN ON THE RECURRENCE AFTER RESECTION FOR HEPATOCELLULAR CARCINOMA ASSOCIATED WITH CIRRHOSIS

S.T. KIM, K.U. LEE, K.S. SUH, T.S. KIM
Department of Surgery, Seoul National University, College of Medicine, Seoul, Korea

We studied to elucidate whether surgical margin is related to recurrence after curative resection of hepatocellular carcinoma in patient with cirrhosis.

Forty patients who underwent curative hepatic resection for hepatocellular carcinoma with cirrhosis between Jan 1988 and Dec 1989 and met the following criteria (histologically proven cirrhosis, solitary nodule less than 10 cm, resection less than formal segmentectomy, no evidence of direct invasion or metastasis) were included in this study. Resection margin was expressed as RMO and RM1 when resection margin is less than 1 cm and equal to or greater than 1 cm, respectively.

There was no statistical difference in the recurrence free rate curve between RMO(n=25) and RM1(n=15). In patients with tumor size equal to or greater than 4 cm, there was no statistical difference in the recurrence free rate curve between RMO(n=12) and RM1(n=5). But in patients with tumor size less than 4cm, there was significant difference between RMO(n=13) and RM1(n=10).

In conclusion, when tumor size is less than 4cm in diameter, recurrence rate can be reduced with more than 1cm of resection margin, but when tumor size is 4cm or greater, the recurrence rate can not be reduced by more than 1 cm of resection margin. So when tumor size is 4 cm or greater, another therapeutic modality may be necessary.

### F067

### 1102 HEPATIC RESECTIONS FOR PRIMARY LIVER CANCER

WU MENG CHAO
Research Institute of Hepatobiliary Surgery, Shanghai Hospital, Shanghai 200433, China

From 1960 to 1989, 5160 patients with liver diseases were admitted in our institute. Among them, 3760 cases were malignant tumors, most of them were primary liver cancer (97%). Among 2210 (60.6%) exploratory cases, 1102 were resectable with a 50% resection rate. There were 992 males and 110 females. Patients ages between 40-60 accounted for 90%. 95% were hepatocellular carcinoma, 85.6% associated with chronic hepatitis or cirrhosis and 25.6% were with tumor smaller than 5cm in diameter. The mortality rate (MR) within 1 month after operation was 1.8%. The total 5-year survival rate (SR) was 28.4%, while in the group of small tumor (<5cm), it was 75%. Before 1977, the 5-year survival rate was 16% and since 1978, it has increased to 30.6%. The data of better results during past decade were analysed and our experience were as follows: (1) Early diagnosis and resection of PLC became possible. The resection rate was 90% in 313 small tumor cases and 5-year SR was 83.3% in patients with tumor smaller than 3cm. (2) Rehepatectomy for early recurrent tumor. 78 cases undergoing 170 times of reoperation with 5-year SR of 36%. (3) Two-stage operation for unresectable large tumor. The 5-year SR was 60% in 26 such cases. (4) Improvement of operative technique made complication rate reduced from 20% to 5% and MR lowered from 8.8% to 0.4%. (5) Postoperative comphensive treatment was important for preventing tumor recurrence and increasing the postoperative SR in our series.

### F068

### COMPLICATIONS OF HEPATO-PANCREATO-DUODENECTOMY

JUNJI TANAKA, SHIGEKI ARII, TADAO MANABE, MASAYUKI IMAMURA, KEN-ICHI FUJITA

Carcinoma of biliary tract commonly invades into neighboring tissues leading to frequent local recurrence after resection. This situation suggests a requirement of extensive local resection for the prophylaxis. One is considered to be hepato-pancreatoduodenectomy(HPD). The present study is to clarify complications in HPD operation. Patients: 23 patients (13 gall bladder carcinoma, 7 bile duct carcinoma and 3 others) underwent HPD operation. Vascular reconstructions of 8 in artery and 4 in portal vein were needed. Extent of hepatectomy was as follows; 7 segmentectomy, 2 cases of 1.5 segmentectomy and 14 over 2 segmentectomy. En-block resection of hepatoduodenal ligament (HLPD) with reconstruction of portal vein and hepatic artery were performed in 5 cases. Among them, 4 operative deaths (3 HLPD) and 1 hospital death cases were accounted. The main causes of death were 2 leakage of pancreaticojejunostomy, 1 leakage of hepaticojejunostomy and 1 liver abscess. Almost all were associated with infection. In the late complications, fatty liver was seen in 8 of 18 cases, but without clinical significance. The longest survival is 67 months with performance status 0. The patients had a good quality of life after discharge. The main cause of 12 late deaths was recurrence of primary diseases. Conclusion: HPD operation itself except HLPD become safe and the quality of life is relatively satisfactory. But, the indication should be applied to the earlier stage for curative operation.

### F069

### THE EFFECTS OF MONOCYTE DERIVED CYTOKINES ON THE PROLIFERATION OF HEPATOMA CELLS

WG JIANG and MCA PUNTIS
Department of Surgery, University of Wales College of Medicine. Cardiff,UK

Monocyte derived cytokines are important in immune regulation and cell proliferation. We studied the proliferation of a hepatoma cell line, PLC/PRF/5 under the effect of 7 monocyte cytokines: IL-1,6,8, GM-GSF, TNFα, TGF-β1, and hepatocyte growth factor (HGF). Cells were co-cultured with recombinant cytokines for 72 hrs, and then quantified by a [Dimethylthiazol-2-yl]-2, 5-diphenyltetrazolium bromide assay.

Result: mean±SEM percentage proliferation compared with control (medium only). *p<0.05 by Student T test.

| | 1:2 dilution | 1:8 dilution | 1:32 dilution |
|---|---|---|---|
| IL-1β(100U/ml) | +12.9±3.8%* | +8.8±7.1% | +4.3±4.3% |
| IL-6(20ng/ml) | +11.9±6.5%* | +2.5±5.1% | +2.5±4.4% |
| IL-8(10ng/ml) | -59.6±7.8%* | -7.9±2.0% | -1.7±6.0% |
| HGF(40ng/ml) | -39.9±3.1%* | -11.4±1.3%* | -8.8±1.9%* |
| TNFα(2.0ng/ml) | -32.9±21.9% | -14.8±11.8% | -0.9±3.7% |
| TGFβ(100RU/ml) | -36.9±2.4%* | -22.4±8.5%* | -18.6±6.9% |
| GM-CSF10(ng/ml) | -32.7±18.1% | -8.7±7.4% | -4.6±2.5% |

These data show that among the monocyte derived cytokines, IL-1 and IL-6 have stimulatory effects on hepatoma cell growth while others especially IL-8, HGF, TNFα and TGFβ are inhibitory. Viability of cells was tested by a trypan blue exclusion test and by morphological changes, there was no difference between control and cytokine cultures, suggesting that some cytokines are inhibitory to cell proliferation and are not cytotoxic. We conclude therefore that monocytes and their derived cytokines probably have a significant role in the host response to hepatoma.

### F070

### THE USE OF TECHNETIUM-99M LABELLED MACROAGGREGATED ALBUMIN IN THE DETERMINATION OF TUMOUR VASCULARITY AND DEGREE OF LUNG SHUNTING IN HEPATOCELLULAR CARCINOMA

*W.Y. LAU, #W.T. LEUNG, #S. HO, ^N. LEUNG, #W. SHIU, *A.K.C. LI
Departments of *Surgery, #Clinical Oncology and ^Medicine, Prince of Wales Hospital, The Chinese University of Hong Kong, Shatin Hong Kong

In the selection of patients for selective internal radiation therapy with yttrium-90 microspheres, it is important to know the vascularity of the tumour and the degree of lung shunting prior to the treatment.

Forty patients with inoperable hepatocellular carcinoma had Technetium-99m labelled macroaggregated albumin (MAA) injected through an angiographic catheter into the hepatic artery. Angiotensin II, a vasoactive agent, was given to enhance the tumour/normal liver tissue ration(T/N ratio). Gamma scintigraphy of the liver and lungs were done. The tumours were localised by matching with computed tomography and technetium-99m tincolloid scans of the liver.

The T/N ratio for 37 evaluable patients ranged from 0.9 to 15 with a median of 3. In 3 patients, the T/N ratio could not be done because there were too little normal liver tissue present for accurate assessment.

Lung shunting varied from 0.7% to 44.5%. 33 of 40 patient had lung shunting of <15%. In 3 patients the lung shunting was over 30%.

Five patients underwent selective internal radiation therapy after MAA. The actual doses were verifed with an intraoperation beta probe during laparotomy and by liquid scintillation counting of biopsy samples taken on representative sites. The correlations between the predicted and the measured doses were good.

Our results showed that MAA can be used to measure the T/N ratio and to determine the lung shunting in patients with inoperable hepatocellular carcinoma.

### F071

### COMPLICATIONS AFTER LAPAROSCOPIC AND CONVENTIONAL CHOLECYSTECTOMY: A COMPARATIVE STUDY

IRIS B. BRUNE, K SCHÖNLEBEN
Chirurgische Klinik, Klinikum Ludwigshafen Ludwigshafen,Germany

Since growing popularity of laparoscopic cholecystectomy (LC) makes a prospective, randomised, comparative study nearly impossible, we compared retrospectively 500 LC with 748 conventional, elective cholecystectomies (CC) for their intra- and postoperative complications. Results:Lethality 0% for LC 0,4% (n=3) for CC. Intraoperative complications requiring an extension of the operation: LC 0,8% (n=4;lesion of the aorta [1], the duodenum [1], the common bile duct [2]).CC ,5% (n=4;lesion of the duodenum [1], the common bile duct [2], the liver [1]).Postoperative complications:LC 2,8%(n=14) of which 5 (1%) demanded a surgical reintervention. CC 3,7% (n=28) of which 5 (0,7) needed a surgical reintervention. The different complications and their therapeutic consequences will be named. Overall complication rates: CC 4,2% (n=32); LC 3,6% (n=18),this number decreasing rapidly with growing experience (OP.Nr.1-30: 13,3%31-70: 7,5%;71-500: 2,7%). Conclusion: Lethality, postoperative and overall complication rates were lower after LC.Particularly the non-specific complications and wound problems were negligeable due to early mobilisation and the small incisions. Intraoperative complications and surgical reinterventions occurred slightly more often, but their number dropped with growing experience. The actual results will be presented.

### F072

### A STUDY OF POSTOPERATIVE PULMONARY FUNCTION AFTER LAPAROSCOPIC CHOLECISTECTOMY

J.A. MAZZEI, J.DIEZ, R, DELBENE, A. FERRERES
Neumonology Division, Department Surgery University Hospital, Otamendi Clinic, Buenos Aires, Argentina

A prospective evaluation study on pulmonary complications in laparoscopic cholecistectomy was performed, comparing the results obtained with those of a similar study previously performed in open cholecistectomy, following the same method.

Patients were evaluated on respiratory symptoms, preoperatory Thorax X rays, forced spirometry, flow volume loop and measurements of arterial blood gases, repeating the same tests 48 and 72 hours later.

The sample consisted of 30 patients (80% of which were women). Average age was 49.57 years ± 11.88 years. Average weight: 66.8 Kg ± 14.5 Kg. Average Height: 1.60 mts ± 0.09 mts.

Ten per cent of the patients had a history of asthmatic conditions. None of them suffered from chronic obstructive pulmonary disease. 30% were smokers and 50% had a history of obesity.

Functional preoperatory tests were normal in the 73% of the cases, 4 cases presented a restrictive pattern and 4 an obstructive pattern.

The average preoperatory vital capacity was of 94.13 ± 18.23. Average preoperatory FEV1: 90.43±19.27. Average FEV1/FVC relation was of 80.60±6.99. Average FEF 25-75% was of 91.90 ±32.86.

PAO2 was normal in 80% of the cases presenting the missing 20% slight hypoxemias. Average peroperative PAO2 was of 83.02 + 10.17 mmHg.

In all cases preoperatory PCO2 was normal being the average 37.02±6.02 mmHg. Thorax X rays showed in 9 of the cases laminar atelectasis (30%) 21 of the cases (70%) presented diaphragmatic bilateral elevation. 13 cases (43%) showed increase in the bronchial marks (tram lines).

Average postoperatory FVC was 72.47 ± 20.07. Average FEV1: 70.40 ± 20.37. FEV1-FVC relation was of 82.40 ± 7.27. The FEF 25-75% was of 69.4+28.82.

Of all the patients evaluated 16 (that is 53%) showed a restrictive pattern.

The average postoperatory PAO2 was of 79.72 ± 10.35 and average PCO2 of 37.06 ± 4.16 mmHg.

Average length of hospitalization was of 1.40 days + 0.71 days and only 3 patients presented clinical complications (10%) such as acute bronchitis in all three cases.

The FVC variation was of –21.67 ± 20.84. FEV1 variation was -20.03 ± 21.08 and that of FEF 25-75% of 22.50 ± 33.64.

PO2 variation was -3.30 ± 12.54 and PCO2 variation of –0.04 ± 6.78 mmHg.

Conclusion: Comparing these findings with those observed in the study performed with conventional cholecistectomy we observe that with laparoscopic cholecistectomy deterioration of pulmonary function, X rays alterations, length of hospitalization period and

### F073

### LAPAROSCOPIC CHOLECYSTECTOMY IN THE OBESE AND MORBIDLY OBESE PATIENT.

J GARZON, GM FRIED, EJ HINCHEY, MJ MAMAZZA, JL MEAKINS, HH SIGMAN, MH WEXLER
McGill University, Montreal, Quebec, Canada

The expanding indications for laparoscopic biliary tract surgery prompted us to assess the appropriateness of employing this treatment modality in obese (O) 20% over ideal weight and morbidly obese patients (MO) (100 1bs over ideal body weight). Our initial concern was that obesity may constitute a contraindication to laparoscopic cholecystectomy (LC). In our McGill series of 907 LC, we have encountered 220 0 and 26 MO patients. The median operative time for the 0 and MO was 75 & 102 min compared with 88 min for all patients. The successfully treated patients in the 0 and MO groups were discharged within 24 hours and returned to their usual activity in one week. Intraoperative complications which required conversion to open cholecystectomy occurred in 9% for 0 and 7.7% for MO patients compared to 5% overall. Reasons for conversion included bile leak (1), hypoxemia (1), CBD injury (2), arterial injury due to trocar insertion (3). Related complications included 3 wound infections, 3 significant hematomas, 1 biloma, and 1 bile leak. Technical recommendations include (1) the 30° scope (2) open insertion of the trocar, (3) initial port selection just above the umbilicus. Our experience supports the fact that 0 and MO are not contraindications to LC.

### F074

### LAPAROSCOPIC CHOLECYSTECTOMY: THE BAaR-EXPERIENCE I

SL JENSEN*, J.DIEZ**, A MON***, P.FUNCH-JENSEN*, R. DELBENE**, R.S.ALMEYRA***, P.WARA*, A.FERRERES**, A.SPIRANZA***
Aarhus University Hospital*, Aarhus,Denmark, Hospital Clinicas**, University of Buenos Aires, Argentina, and Clinico Britanico***, University of Rosario, Argentina.

New year 1991 the Universities of Buenos Aires (B) (Hospital clinicas), Rosario (R) (Clinico Britanico, Argentina and Aarhus(Aa), (Aarhus Kommunehospital), Denmark introduced; laparoscopic cholecystectomy as standard treatment of cholecystolithiasis and a collaboration was established in order to evaluate its mortality and morbidity. During the first 10 months of 1991 18 surgeons (10 consultants [6.9%];and 8 chief residents [3.1%]) performed 425 (B:n=134,Aa:n=141 and R: n=150) laparoscopic choleycystectomies for symptomatic cholecystolithiasis (95 [22.3%] with chronic cholecystitis and: 30 (7.1%) with moderate to severe acute cholecystitis. CBD stones were found preoperatively in 23 (5.5%) of the patients. They were removed endoscopically. Preoperative cholangigraphy was performed routinely in Rosario and selectively in Buenos Aires (n=3) Aarhus (n=1). However, the number of patients with residual stones was the same in Rosario (n=3) Buenos Aires (n=1)and Aarhus (n=2). The residual stones were removed endoscopically (n=4), laparoscopically (n=1) or surgically (n=1). The mean duration of the laparoscopic operations was 72 minutes (range 30-360 min). They were performed with one death (0.2%) and a morbidity rate of 7.6% (major (biliary leakage (n=4), stenosis of the CBD (n=1)): 1.0%; minor (wound infections (n=11), abdominal wall heamatoma (n=7), residual common duct stones (n=6), mild subcutanoues emphysema (n=4); respiratory problems (n=2), cardiovascular problems (n=2), intraabdominal abscess (n=1):6.6%. There was one injury to CBD (0.23%) which required reconstructive biliary surgery. Seventeen patients (4%) required conversion to open cholecystectomy because of technical difficulties. Ninety-one percent of the patients was on a regular diet the morning after surgery. The mean hospital stay was 1.6 days (range 0-14 days)and the mean time of return to full activity was 6.35 days (range 3-21 days). The study shows that laparoscopic cholecystectomy can be performed safely by the average surgeon in university hospitals and it indicates that routine cholangiography has no advantages or disadvantages compared with selective cholangiography.

### F075

### LAPAROSCOPIC CHOLECYSTECTOMY UNDER EPIDURAL ANESTHESIA – AN IDEAL COMBINATION OF MINIMAL INVASIVE SURGERY AND MINIMAL INVASIVE ANESTHESIA

LEXER G.W.M.D.[1], LEXET G.CH. M.D.[2], REICHENAUER A. M.D. [2] LEHOFER F.M.D. [3], PIMPL W.M.D. [1], BOECKL O. M.D.[1]

Ist. Dept of Surgery, LKA Salzburg [1]; Depts of Surgery [2] and anesthesiology [3], Friesach; AUSTRIA

Epidural anesthesia (EPA) has been reported to exert beneficial effects in surgical procedures. In this retrospective study we evaluated the outcome of EPA in pat. undergoing laparoscopic cholecystectomy (1ap. CHE). Unselected 61 patients (39 female, 22 male), mean age 46,9 years (20/86), mean weight 71,7 kg (46/125) with symptomatic gallstone disease were admitted to our lap. CHE-program maintaining analgesia with EPA. Preoperative patient selection and indication for lap. CHE were unchanged.

Ventilatory measurements and arterial blood gas analyses were performed preoperatively (1) in the horizontal supine position with T5-T10-level of analgesia, (2) after intraabdominal insufflation of $CO_2$ for pneumoperitoneum and (3) at the end of the procedure. During the procedure the patients were maintained with oxygen insufflation of 3 1/min through an anesthetic face mask Interaoperative sedation was given if necessary.

No significant changes of minute ventilation or arterial blood gas measurements were observed. Furthermore, no intra- or postoperative either anesthetic or surgical complications occurred.

In review, our experience shows that EPA in lap. CHE is associated with almost no risk and is an acceptable anesthesiological technique even for unselected patients.

### F076

### OUT-PATIENT LAPAROSCOPIC CHOLECYSTECTOMY

R.F. PACE, R. SMITH.
Department of Surgery, Queens University, Kingston General Hospital, Kingston, Ontario Canada.

Laparoscopic cholecystectomy has rapidly become the treatment of choice for patients with symptomatic gallstones. This has, in part, in part, been due to the significant decrease in post-operative pain, hospital stay, and a rapid return to normal activities. Of our first 200 patients scheduled for laparoscopic cholecystectomy, there was a 6% rate of conversion to 'open', with an average post-operative stay for all patients 1.8 days. If only successful laparoscopic cholecystectomy patients are included, the post-op stay is 1.5 days. During our last 100 cases we have been treating selected patients as out-patients. To be considered for out-patient therapy patients must be highly motivated, live in or near the city and have a significant other at home to care for them. Eighteen of 21 patients selected for out-patient therapy were successfully discharged from hospital within 6 hours of surgery. None of these patients required re-admission to hospital, nor did they have any complaints regarding their early discharge. The 3 patients scheduled for out-patient laparoscopic cholecystectomy but who required admission following surgery had nausea and vomiting which precluded their early discharge. All 3 of these patients were discharged the following day We conclude that out-patient laparoscopic cholecystectomy is a viable alternative to admission in as many as 1/5th of patients The option is safe and acceptable in the motivated patient.

### F077

### ESWL AND LAPAROSCOPIC CHOLECYSTECTOMY – AN APPROPORIATE PROCEDURE IN LARGE BARREL SHAPED GALLBLADDER STONES

MEISER G. M.D, LEXER G. M.D. WACLAWICZEK H. M.D, BOECKL O. M.D.
Ist. Dept of Surgery, LKA Salzburg, Austria

Laparoscopic extraction of large barrel shaped gallbladder stones through a small paraumbilical incision can be difficult and sometimes impossible. In our surgical dept we performed ESWL (LITHOSTAR PLUS, Siemens Comp.) in 52 patients 37 female and 15 male - prior to laparoscopic cholecystectomy. The single stone diameter was between 3 cm and 4,5 cm. 41 patients suffered from pure cholesterol, the other 11 from calcified stones. On average 3200 + 1250 shock waves with an energy level of 750 bar were applied The ESWL treatment time ranged from 15 to 45 minutes (21 + 16 min.)

In all cases stones could be disintegrated successfully to a final fragment size of 1–12 mm. Laparoscopic cholecystectomy was carried out 1 day post ESWL routinely without problems. The gallbladder could be easily removed using a 1,5–2 cm small incision of the abdominal wall.

No complications were seen in the postoperative course and all patients were discharched on the 4th postoperative day.

### F078

### HYALURONIC ACID ENHANCED $^3$H-5-FLUOROURACIL UPTAKE IN THE MALPERFUSED RAT LIVER

W.HE, E.S. KLEIN, S.S. ASCULAI, J.A. NELSON, R.E. FALK
Department of Surgery, University of Toronto School of Medicine, Toronto, Ontario, Canada

Biological and chemical properties of hyaluronic acid (HA) qualify this macromolecule as a prospective carrier of drugs particularly for targeting to different tissues. To assess this we studied the effect of HA on tritiated 5-Fluorouracil ($^3$H-5-Fu) uptake by acute and chronic malperfused rat liver tissue using two models:

1. Ischemia/reperfusion: Four groups of rats (A, B, C, D) were subjected to 30 minutes liver ischemia followed by a period of reperfusion. Two sham (E,F) groups served as controls. All groups received intravenous (i.v.) $^3$H-5-Fu at the end of reperfusion period with (A, C, E) or without (B, D, F) i.v. HA (15mg/kg). Groups C and D also underwent a "once-through" liver perfusion before liver tissue harvesting to eliminate blood factor.

| Group(n=) | A(9) | B(9) | C(6) | D(6) | E(8) | F(9) |
|---|---|---|---|---|---|---|
| meanCPM | 42487 | 45392* | 37372 | 40790* | 45591 | 46261 |
| SEM | ±569 | ±839 | ±468 | ±665 | ±1328 | ±536 |

2. Liver-implanted tumor: Two groups of rats with liver-implanted rate mammary carcinoma received i.v. $^3$H-5-Fu alone or combined with i.v. HA. Liver (LVR) and tumor (TMR) tissues were processed for radioactivity counting.

| Tissue(n=) | TMR(9) | TMR(HA)9 | LVR(9) | LVR(HA)(9) |
|---|---|---|---|---|
| meanCPM | 314 | 461* | 1979 | 2237* |
| SEM | ±25 | ±61 | ±133 | ±122 |

In the results, *means P<0.05 by ANOVA-1, vs. untreated groups

Conclusions: The exogenous HA may preferentially target depleted tissue and therefore enhance drug delivery to such tissue.

### F079

### THE EFFECT ON LIVER METASTASES OF CIRCADIAN PATTERNING OF CONTINUOUS HEPATIC ARTERY INFUSION (CHAI) OF FUDR

M.F.KEMENY, G. ALAVA, J. OLIVER, F.B.SMITH
St. Vincent's Hospital and Medical Center, New York City, New York, USA.

Previous studies have shown that using a circadian patterned CHAI of FUDR has lowered the toxicity of the infusion and allowed for higher doses of FUDR to be given. The question of whether this decrease in toxicity is accompanied by an increase in antitumor efficacy has not been demonstrated. A hepatic artery catheter was placed in 18 Fisher Rats 10 days after an adenocarcinoma had been implanted subcapsularly in the liver. Nine animals received a constant CHAI of FUDR at a dose of 10mg/kg/d for 14 days. Another 9 animals received a circadian timed CHAI of 15mg/kg/d of FUDR. In this pattern 67% of drug was delivered between 3pm and 9pm each day. Four of the rats on the constant schedule died of toxicity from the infusion. All of the animals with the constant infusions had a decrease in the tumor volume from 87 to 100%. In the animals given circadian cycled continuous FUDR non died of toxicity. One had an increase in tumor size while the rest had a decrease in size from 94 to 99%. There was no significant difference in the response rates in the two groups. This study shows that with circadian timing more drug can be delivered with lower toxicity and have an equal anti-tumor efficacy to constant CHAI.

### F080

### REPEATED TRANSIENT DEARTERIALIZATION OF A LIVER TUMOUR IN THE RAT

LIQING WANG, BO G. PERSSON, BENGT JEPPSSON AND STIG BENGMARK
Depat. of Surgery, Lund University, Lund, Sweden

This experiment was designed to elucidate the optimum period of repeated dearterialization of liver tumours without giving rise to collaterals. Repeated dearterializations were achieved with a minioccluder implanted around the hepatic artery. Forty rats assigned to receive intermittent dearterialization for 0 (sham operation), 30, 60, 120 and 180 minutes respectively (A-E n=8 in each) and compared to permanent dearterialization (F n=8). The tumour size was measured before and after 5 days of daily dearterializations. Another 18 rats were allocated to repeated transient dearterializations for 2 hours/day prolonged to 18 days (G), control (H) and permanent dearterilization (I) (n=6 in each).

Results: The tumour growth was almost totally retarded in groups D, E and F after 5 days of dearterialization and highly significantly less compared to group A, B and C (p<0.01). The growth rate was modestly retarded in group C compared to groups A and B (p<0.05). After 18 days of dearterialization the tumours resumed to grow though the growth rate in group G was still much less compared to groups H and J (p<0.05). No collaterals could be demonstrated in D and minor collaterals in E in contrast to fully developed collaterals in F and J.

Conclusion: The optimun period of arterial blockade retarding this liver tumour without giving rise to collaterals is between 2 and 3 hours.

### F081

### WATER-JET-COOLED ND: YAG LASER COAGULATION OF RAT LIVER METASTASES: SELECTIVE TUMOR DESTRUCTION

R. VAN HILLEGERSBERG, W.J. KORT, F.J.W. TEN KATE, O.T. TERPSTRA
Depts of Experimental Surgery and Pathology, Erasmus University, Rotterdam, The Netherlands.

We investigated the use of laser in treating hepatic metastases from colon cancer. A syngeneic colon carcinoma CC531 was implanted in the liver of 68 Wag/Rij rats; 20 days after inoculation, when the tumor diameter was about 5 mm, laser therapy was performed with a water-jet-cooled Nd: YAG laser at energies of either 600 J, 850 J, 1,200 J, 1,700 J or 2,400 J and a power setting of either 10 or 20 W. Liver damage was determined on the basis of histological sections taken on day 1 after treatment using a computer integrated image analyzer and serum ASAT/ALAT levels measured on day 1 and 2. To assess liver function an antipyrine clearance test was performed on day 2 after treatment. Sections of day 36 were used to evaluate tumor remission Light microscopy on day 1 showed coagulative necrosis up to 10 mm in diameter. Multiple regression analysis of the parameters used indicated a significant relationship between laser energy and liver damamge ($R^2$=0.05, P=0.01).At 20 W liver damage was 22% larger than at 10 W. Tumor damage also increased with energy applied, resulting in complete tumor destruction at 2400 J, whereas massive tumor outgrowth occured in control animals. No deterioration in liver function was found as measured by antipyrine clearance. The results of this study show the ability of the water-jet-cooled Nd:YAG laser to produce selective tumor necrosis with minimal liver damage. Treatment of several superficial tumors at different liver lobes would be possible, thus reducing surgical trauma and diminishing complications like bleeding and liver failure.

### F082

### COLORECTAL LIVER METASTASIS (LM): EXTRAHEPATIC DISEASE, FOUR OR MORE LM, AND CLEAR MARGIN SMALLER THAN 1 CM ARE NOT AUTOMATIC CONTRAINDICATIONS TO LIVER RESECTION (LR)

D.ELIAS, PH. LASSER, PH. ROUGIER, F. FARHAT.
Gustave-Roussy Institute Cancer Center, 94805 VILLEJUIF, FRANCE.

AIM: TO retrospectively study the prognostic value of the 3 criteria commonly recommended as contraindication to LR during the last ten years: extrahepatic disease, 4 or more LM, and clear margin smaller than 1 cm.

Patients (pts): 85 pts with 2 years minimal follow-up had LR for LM; 20 had intraabdominal or pulmonary extrahepatic disease (also resected), 16 had 4 or more independent LM 17 had clear margin equal zero, 24 between 0 and 5 mm and 15 between 5 and 10 mm. One post-operative death occurred (1,2%).

Results: crude and disease free survivals were similar for these pts and for the pts with classical good prognostic criteria. Actuarial 2, 3, 4, and 5 year crude survival was 77%, 63%, 45% and 28% respectively; disease free survival was 76%, 51%, 31% and 17% respectively for each group of pts.

Conclusion: these 3 classical contraindications to LR have no real value. Prognostic determinants of LR for LM from colorectal cancers always appear very vague in the literature and should be studied in an extensive multicentric prospective study without exclusion criteria.

### F083

### ISOLATED LIVER PERFUSION WITH MITOMYCIN-C IN PATIENTS WITH HEPATIC METASTASES.

LM DE BRAUW, A MARINELLI, HJ KEIJZER+,JH VANBOCKEL, CJH VAN DE VELDE
Dept of Surgery and Oncology+, University Hospital Leiden, The Netherlands

Nine patients with irresectable colorectal hepatic metastases (percent hepatic replacement 25-75%) were subjected to new method of Isolated Liver Perfusion (ILP).

Procedure: Surgical isolation and subsequently perfusion of the liver coupled to an extracorporeal circuit during 1 h.20 min. with MMC (dose 30 mg/m$^2$).

Results: All patients survived the procedure. One patient died after 40 days following the operation due to Veno-occlusive Disease (VOD). Two other patients developed this syndrome, and needed a peritoneal-venous shunt. The liver function tests LDH, SGOT, SGPT and bilirubin increased significantly after ILP but all returned to normal within one week .

Responses: One patient had an objective complete response on CT scan (alive NED > 9mo), and seven of eight evaluable patients had an objective partial reponse (4 alive > 10–16 mo.)

Conclusion: ILP was well tolerated by the patients. ILP with MMC induced significant toxicity and resulted in objective tumor regression in 7 of 8 evaluable patients, including one complete response.

### F084

### CONTINUOUS SIMULTANEOUS INTRAARTERIAL (IA) AND INTRAVENOUS (IV) THERAPY OF LIVER METASTASES OF COLORECTAL CARCINOMA. RESULTS OF A PROSPECTIVE RANDOMIZED TRIAL.

F. SAFI, H. G. BEGER
Department of General Surgery, University of Ulm, Steinhovelstr. 9, D-7900 Ulm, FRG

From 1982 to 1990 276 patients (pts.) with hepatic metastases of colorectal carcinoma were admitted in our clinic. 159 pts. did not get a treatment. Further 91 pts. were treated regionally and the remaining 26 pts. underwent a surgical resection of the metastases. Since pts. died under IA chemotherapy of extrahepatic spread of the metastases, we started a randomized controlled trial to compare the effficacy of IA to continuous simultaneous IA and IV chemotherapy of liver metastases. The first 20 pts. were treated only IA (pilot group PG). The other 71 pts. were stratified by primary tumor stage and the percentage of liver involvement and were then randomly assigned before surgery to receive either IA (IA group n=34pts.) or IA and IV therapy (IA/IV group n=37 pts.). Intervention: 14 days continuous infusion of FUDR each month (m) (0.2 mg/kg/day in all IA treated pts. and 0.3 mg/kg/day in the IA and IV group; infusaid pump mo. 400, dual).

Results: The complete and partial response rate was 59%, 57% and 50% in the IA, IA/IV and PG respectively. 79% of the IA group, 75% of the PG and 51% of the IA/IV group developed extrahepatic disease in a median follow-up time of 24 m (p<0.01). Hepatic and systemic toxicity in the IA and IA/IV group were acceptable. No significant difference in survival was found between the IA and IA/IV groups (p=0.09). The difference in extrahepataic disease free survival was between the two randomized groups significant p<0.01.

### F085

### IDENTIFYING PATIENTS WITH CHOLELITHIASIS UNLIKELY TO BENEFIT FROM SURGERY

R.W. DICKINSON, L.H.OAKES, J.S. GANI
Department of Surgery, John Hunter Hospital, Newcastle, NSW, Australia.

There is a 34% incidence of post cholecystecomy pain[1]. This study was undertaken to identify those patients with cholelithiasis unlikely to benefit from surgery. Patients with gallstones who were referred to a general surgical clinic were studied. A single investigator using a detailed questionnaire and an objective biliary symptom scoring system completed an objective and a subjective assessment of these patients. All patients also underwent choleycystokinin octapeptide cholescintigraphy (OP-CCK-Scan)[2]. Patients were reviewed after six months (non-operative group) or six months post-cholecystectomy.

Results: 34 cases were included in the study. Following objective and subjective symptom assessment these patients were then categorised. The association between subjective and objective scores was almost linear with a Cramer coefficient of 0.93. 17 patients were classified as having definite biliary pain. All of these underwent surgery and all were significantly improved at mean follow-up of nine momnths. 70% of theis group had an abnormal OP-CCK Scan. In those patients classified as having possible biliary pain 37% had an abnormal OP-CCK Scan. Most of these patients were managed non-operatively and 50% had improved at six month review, although none were cured. Six patients were classified as having non-biliary pain, 30% of these had abnormal OP-CCK-Scans. All but one of these patients was managed non-operatively, though this group did not improve with time. Finally, in a group of asymptomatic controls with gallstones all patients had normal OP-CCK Scans.

Conslusions: The use of a biliary symptom questionnaire whether objectively or subjectively scored results in accurate categorisation of patients into a group unlikely to have post cholecystectomy symptoms and two other groups in which post cholecystectomy symptoms are more probable. In those patients with definite biliary pain OP-CCK Scanning tends to support the clinical categorisation of patients but appear to offer no additional benefit in terms of outcome prediction. Those patients with possible biliary pain are more likely to have a normal OP-CCK Scan. It is in this group of patients that cholecystokinin octopeptide cholescintigraphy may help identify those patients more likely to suffer from the post-cholecystectomy syndrome.

References
1. Bates, T et al. Influence of cholecystectomy on symptoms. Br. J. Surg. 1991; 78 : 964-967.
2. Yap, L et al. Acalculous Biliary Pain: Cholecystectomy Alleviates symptoms in Patients With Abnormal Cholescintigraphy. Gastroenterology 1991; 101:786-793.

### F086

### INCIDENCE OF *HELIOBACTER PYLORI* (HP) IN PATIENTS WITH SYMPTOMATIC CHOLECYSTOLITHIASIS

F.E. LÜDTKE, H. NIEMEYER, H. ÖHLER, F.E. BAUER*, R. TEGELER**, G. LEPSIEN
Dept. of General Surgery, Clinical Pharmacolgy* and Med. Untersuchungsamt**, University of Göttingen, Germany

The incidence of HP colonisation in the antrum of the stomach in patients with symptomatic cholecystolithiasis was determined (n=117, m:f = 1:2.25, median age 53 years, range 21-86 years) in preparation for a prospective study. The results were compared to a control group of healthy patients with the exact same age and sex distribution.

Material and Methods: The HP status was determined using positive microbiological proof and/or the13 C-urea-breath test. In addition, 51 surgical gallbladders were examined for the presence of HP. In 51 patients, a follow-up clinical examination and laboratory status was performed 7 months after the cholecystectomy and the post-operative course analysed with respect to any complaints reported.

Results: 1.) HP was found in 54 (46.2%) of 117 patients with cholecystolithiasis, whereas HP was only found in (27.4%) of the control group ($p<0.05$). 2.) No evidence for HP could be found in preparations of the gallbladders removed. 3.) 25.5% of the 51 cholecystectomy patients examined post-operatively complained of mild yet disturbing symptoms whereby no evidence of a biliary origin of these complaints could be found. No association between HP status and the post-operative course could be elicited in the small patient collective.

### F087

### COST ANALYSIS OF PROTOCOLS FOR THE INVESTIGATION AND TREATMENT OF COMMON BILE DUCT STONES

DM SCOTT-COOMBES, JN THOMPSON
Department of Surgery, Hammersmith Hospital, Du Cane Road, London, England

Laparoscopic cholecystectomy is now widely accepted as the operation of choice for patients with symptomatic gallstones but controversy exists over the management of patients with common bile duct stones. The optimal way to diagnose ductal stones is cholangiography, but this exists in several forms and may be performed before, during or after surgery.

For surgeons performing laparoscopic cholecystectomy the choice of treatment for patients with common bile duct stones is either open operation or laparoscopic cholecystectomy preceeded or followed by endoscopic removal of the stones.

Given the many permutations regarding the investigation and treatment of ductal stones we performed a cost analysis study of different management protocols based on the varying workloads and the cost of the individual procedures. By combining our results of this economical survey with the clinical considerations we have attempted to identify the optimal approach to this condition in different patient categories.

A policy of selective operative cholangiography with postoperative intravenous cholangiography for the technical failures is recommedned. Low-operative risk patients are probably best managed by conversion to an open operation, while postoperative stone clearance should be reserved for higher risk patients and those whose stones are identified after cholecystectomy.

### F088

### SENSE OR NONSENSE OF ROUTINE PREOPERATIVE UPPER GASTROINTESTINAL ENDOSCOPY IN PATIENTS WITH SYMPTOMATIC GALLSTONE DISEASE.

P. SUNGLER, M. HEINERMAN, O. BOECKL
1st Surgical Dept. and Ludwig-Boltzmann-Institute for Experimental and Gastroenterologic Surgery, Landeskrankenanstalten Salzburg, Austria

Routine preoperative upper gastrointestinal endoscopy (UGE) in patients with documented and symptomatic gallstone disease is carried out before cholecystectomy. Of 1100 patients, who had consecutive cholecystectomies performed, 250 had gastroduodenal disease, discovered at endoscopy. Only 5 of those had an associated gastroduodenal operation at the time of cholecystectomy. Mainly, upper gastrointestinal disease was treated conservatively. 50% of the cases had delayed cholecystectomy due to the diagnosis of ulcers. Four unexpected cancers of the stomach (TIS and $T_1$) were found.

11 patients were ruled out ot have alternative treatment such as extracorporeal shock-wave lithotripsy (ESWL).

Despite the current trend toward critical analysis of costs of medical care, we believe in the clinical value of routine UGE before cholecystectomy or ESWL, complemented by indicated endoscopic retrograde cholangiopancreaticography.

### F089

### LONG TERM (10 YEARS) FOLLOW-UP OF CONVENTIONAL CHOLECYSTECTOMY

J. KONSTEN MD, DJ. GOUMA MD. MAASTRICHT
University Hospital, Maastricht, The Netherlands.

INTRODUCTION: Long term follow-up of different (new) treatment modalities for gallstone disease (with or without removal of the gallbladder) is limited. However stone recurrence, biliary and postcholecystectomy symptoms should also be evaluated to select the optimal procedure. Therefore, long term follow-up of conventional cholecystectomy (the gold standard) was studied with emphasis for postcholecystectomy symptoms. PATIENTS AND METHODS: 351 patients (263 female and 88 male) underwent a conventional cholecystectomy more than 10 years ago. Follow-up was obtained, (using questionnaires) in 93% and bloodsamples of symptomatic patients alive in 69%.

RESULTS: The operative mortality was 0.3%, the procedure related morbidity 5% and follow-up was obtained in 325 patients of whom 82% were symptom free. Overall, 60 patients (18%) had complaints after 10 years and stone recurrence had been found in 5 patients (1.5%). Ten other patients (3%) had biliary tract related complaints although bloodsamples revealed no abnormalities. Additionally, 45 patients were found to have atypical symptoms at the time of follow-up.

CONCLUSION: Ten years after surgery, conventional cholecystectomy showed to cure 82% of the patients. Five % had biliary tract related symptoms, whereas 13% had atypical symptoms. New techniques should be compared with the follow -up of cholecystectomy to select the optimal procedure for the individual patient.

### F090

### PLASMA CHOLECYSTOKININ AFTER CHOLECYSTECTOMY

IS BAILEY, TN WALSH, ADK HILL, S JAZRAWI, TPJ HENNESSY, CD JOHNSON.
University Surgical Unit, Southampton General Hospital, Southampton & Surgical Unit, Trinity College, Dublin.

Receptors for the peptide hormone cholecystokinin are found on many abdominal organs. Exogenous CCK causes a variety of abdominal symptoms and abnormal endogenous release of CCK may be involved in such symptoms. Cholecystectomy may alter CCK release by altering postprandial bile flow. Abnormal circulating levels of CCK may be responsible for the high incidence of dyspeptic symptoms reported after cholecystectomy.

Using a specific CCK radioimmunoassay integrated plasma CCK was measured for one hour after a meal of 60ml corn oil in instant mashed potato. Integrated plasma CCK (pmol/l/hr) was 119 (31-215) in normal adult controls (n=13), 145 (22-517) in patients with symptomatic uncomplicated gallstones (n=22), and 229.5 (63-522) in patients after cholecystectomy. Integrated plasma CCK was 115 (62-360) in post cholecystectomy patients with unexplained dyspeptic symptoms (n=8) and 290 (94-522) in patients without symptoms (n=12) (p=0.06 [Mann Whitney]). Integrated plasma CCK was significantly higher in the post-cholecystectomy patients compared with the controls (p=0.02 [Mann Whitney]).

Postprandial plasma CCK concentrations are significantly increased after cholecystectomy. Increased CCK is not related to post-cholecystectomy dyspepsia. Dyspeptic patients after cholecystectomy have lower postprandial concentrations of CCK than asymptomatic patients. This would be consistent with these patients having reduced gastric emptying compared to the asymptomatic post-cholecystectomy patients.

### F091

### EFFECT OF CARBOHYDRATE-RICH DIET AND PORTAL BACTEREMIA ON PIGMENT STONE FORMATION AND BILE COMPOSITION IN HAMSTER

K.S. SUH, S.W. KIM, Y.H. PARK
Department of Surgery, Seoul National University, College of Medicine, Seoul, Korea

We performed an experimental study to see whether a high-carbohydrate (CHO) diet induces pigment stone formation in hamster. In addition, we studied the effect of biliary tract infection induced by portal bacteremia on this high-CHO diet induced stone formation. Ninety male hamsters were divided into 3 groups (Group 1: control chowfed group, Group 2: high-CHO fed group, Group 3: high-CHO fed plus portal bacteremia group). The high-CHO diet was 65% CHO(43% in control chow), mainly composed of rice, and portal bacteremia was induced by injection of endogenous E. coli into the portal vein. The hamsters were sacrificed after 12 weeks of feeding. The stones were analyzed by infrared spectrophotometry and the hepatic bile was analyzed by using commercial Kit. No stones were found in Group 1 but black-colored stones were found in 24 out of 30 animals in Group 2 ($p<0.0001$ vs Group 1). In Group 3, 12 animals survived and all of them developed stones. There was no significant difference between Group 2 and 3 in stone formation. The stone composition was similar to that of human calcium bilirubinate stones. In hepatic bile analysis, the phospholipid and total bile acid level in group 2 were higher than those of Group 1($p<0.05$). In conclusion a high-CHO diet is a possible important etiologic factor of gallstone formation, especially calcium bilirubinate stones. In addition an increased level of phospholipid and total bile acid in hepatic bile may contribute to high-CHO diet induced gallstone formation.

### F092

### FREE RADICAL AND PIGMENT GALLSTONE FORMATION

C.LIN, T,SHEN, X.B. FU, X.S.ZHOU
Department of Surgery, Third Teaching Hospital, Beijing Medical University, Beijing 100083, China

Since free radical signal in pigment gallstones (PS) appears in vivo, the role of superoxide free radical (SOFR) in the formation of PS was explored in this study. In S group, a stricture at the common bile duct (CBD) of guinea pigs were created by ligation. In S+V group, 10mmg/kg of Vit.E and C were injected daily since 3 days before CBD ligation. In C group, only laparatomy was performed as control. One week after operation, the incidence (INC) of PS, total bilirubin (TBr), indirect reactive bilirubin (UCB), Ionized calcium (ICa) contents, and SOFR scavenge rate (SR) of gallbladder bile were measured and listed below. When the UCB and ICa contents increased and SR decreased, the INC elevated (S vs C). Once the SR recovered, even the UCB and ICa remained high, the INC was supressed (S+V vs S).

| | C group | | S group | | | S+V group | | |
|---|---|---|---|---|---|---|---|---|
| | n | Mean ± SD | n | Mean ± SD | | n | Mean±SD | |
| INC | 0/15 | | 14/16 | | @ | 5/14 | | @ |
| TBr (uM) | 7 | 13.7 ± 8.1 | 16 | 40.8 ±30.3 | @ | 13 | 69.2 ± 40.3 | |
| UCB (uM) | 7 | 5.2 ± 3.4 | 16 | 12.6 ± 8.9 | @ | 13 | 16.9 ± 11.5 | |
| ICa (uM) | 11 | 281 ± 113 | 16 | 765 ± 441 | @ | 14 | 720 ± 304 | |
| SR | 9 | .944 ± .048 | 14 | .683 ± .311 | @ | 14 | .921 ± .082 | @ |

(@: S vs C, S+V vs S, Chi-square (INC) or t-test, $p<0.05$)

CONCLUSION: the presence of superoxide free radical was essential in pigment gallstone formation. (*supported by National Natual Science Foundation of China)

### F093

### CALCIUM SALTS OF FATTY ACIDS IN GALLSTONES. ARE THEY MARKERS OF BILE INFECTION BY E.COLI ?

F LOMBARDO, V GARGANO, F CETTA
Institute of Surgical Pathology University of Siena Italy

A content of calcium salts of fatty acids, mainly consisting of calcium palmitate (CP) greater than 10% of stone dry weight was found in 79 of 960 consecutive patients with gallstones, who underwent surgery. In these patients stones were homogeneously brown pigment stones in 48 cases, black and brown in 9 and cholesterol or mixed stones in 2 cases. In the other 19 cases CP was found in the brown periphery of composite stones or in same brown stones, associated with mixed or cholesterol stones in the some site. Bile infection by E.coli was associated with both brown pigment and calcium palmitate containing stones. In particular E.coli overgrowth was found in 100% of brown pigment stones in the common duct, all with considerable CP content and in 98% of brown gallbladder stones.

It is suggested that: 1) Stones with a CP content greater than 10-15% are associated and usually determined by bile infection. These stones are mainly brown pigment (97.4%, 75 of 77) and are mainly found in the common duct postcholecystectomy. 2) Not the simple bacterial overgrowth in the bile, but also the type of bacteria, in particular the presence of E.coli, alone or in association with other bacteria, in addition to other factors as age of patients and site of stones, are of importance for calcium palmitate precipitation. 3) Palmitate and carbonate are anions mutually exclusive of each other in gallstones. 4) The presence of CP both in the stone centre and periphery can be used as a marker to determine whether postcholecystectomy stones are true recurrent or residual stones. In particular, E.coli overgrowth was found in 100% of patients with common duct brown pigment stones all containing CP, and in 98% of "pure gallbladder brown stones. On the other hand, a bacterial count in the bile greater than $10^5$ CFU per ml was not found in 6-9% of patients with brown stones (in particular in those with composite stones or with brown stones only in the gallbladder). Calcium palmitate was found in 33.6% of all stones associated with bile contamination and in 51.3% of stones associated with bile infection by E.coli.

### F094

### GALLBLADDER MOTILITY DETERMINED BY INFUSION CHOLESCINTIGRAPHY BEFORE AND AFTER TOTAL GASTRECTOMY

M. MILICEVIC, M. PETROVIC, V. ARTIKO, V. OBRADOVIC, G. JANKOVIC I.K. KOSTIC
Institute for Digestive Diseases and Nuclear Medicine Service, University Clinical Center Belgrade 11000, Yugoslavia, Koste Todorovica br.6

The aim of this study was to determine gallbladder (GB) motility in patients before and after total gastrectomy and to compare the motility pattern to a stimulated and nonstimulated control group. Infusion cholescyntigraphy was performed with the patient supine using a ROTA scintillation camera connected to a MicroDelta computer system. Data acquisition was performed during a 3 hour period of 4mCi99m-Tc-EHIDA continuous infusion, preceded by a bolus injection with increased concentration. Stimulation was done by drinking fresh egg yolk. There were 12 pts. in the control (C) group without stimulation, 10 pts. on the control group with stimulation (CS) and 5 pts. before total gastrectomy (BTG) and the same 5 pts. after total gastrectomy (ATG).

Gallbladder filling (GBF) in group C lasted 75.5 +/- 20.5 min., and gallbladder emptyinga (GBE) lasted 58.8 +/- 28.0 min. The ascending/descending TA curve ratio, determined as slope ratio was X=1.26+/-0.9 (integral X=2.22+/-1.1. Mean ejection fraction (EF) was 42.1% +/- 7.2 (SE=2.1). In the CS group BF filling lasted 66.1 +/- 27.0 min and GBE 35.1 +/- 12.1 min. The ascending/descending TA curve ratio was 0.81 +/- 0.6 (integral 3.1 +/- 1.6. The mean EF was 77.5 +/- 19.2%. In the BTG group GBF lasted 62.5 +/-27.6 min, GBE 41.5+/- 29.0 Min. The ascending/descending TA curve ratio was 0.99 +/- 0.6 (integral 3.93 +/- 2.8. The mean EF was 89.5 +/- 8.4%. In the ATG group GBF lasted 77.2 +/- 15.1 min, GBE 16.7 +/-6.8 min, the ascending/descending TA curve ratio was 1.72 +/-1.1 (integral 1.9 +/-0.9. The mean EF was 9.0 +/- 0.4. All stimulated patients had a shorter GBE time (p) in comparison to the nonstimulated group. The short GBE time in the ATG group (p) can be explained by an very low EF (p).

Our results demonstrate clearly that after TG the GB has impaired emptying which is not significantly influenced by oral stimulation which might contribute to gallstone formation.

### F095

### $^{131}$I-LIPIODOL TARGETED INTRAHEPATIC IRRADIATION OF PRIMARY LIVER TUMOURS

J R NOVELL, A HILSON, A GREEN, K E F HOBBS
Hepatobiliary and Transplantation Unit, The Royal Free Hospital School of Medicine. London

The therapeutic potential of $^{131}$I-Lipiodol was investigated in 15 patients with hepatocellular carcinoma (HCC) and 8 patients with cholangiocarcinoma (CCA). Patients received one or two doses of $^{131}$I-Lipiodol via hepatic arterial injection. The mean total adminstered activity was 953 (SD 477) MBq in HCC and 723 (SD 559) MBq in CCA. One patient with CCA retained $^{131}$I-Lipiodol. The cumulative radiation dose was 9.6 Gy to tumour, 6.4 Gy to liver and 1.5 Gy to lung. The patient remains asymptomatic 30 months from the start of treatment, whereas the remaining 7 patients have exhibited tumour progreesion. The mean survival in CCA was 11.6 (SD 14.5) months. All 15 patients with HCC retained $^{131}$I with tumour:liver ratios of up to 30:1. The mean cumulative radiation dose was 34 (SD 32) Gy to tumour, 3.5 (SD 1.4) Gy to liver and 4.7 (SD 2.1) Gy to lung. The mean dose per adminstered activity was 3.8 (SD 4.3) cGy/MBq. Partial response (reduction in tumour size > 50%) was observed in 6 patients (40%). The mean survival was 8.3 (SD 8.6) months.

$^{131}$I-Lipiodol can deliver highly selective internal irradiation to foci of HCC with evidence of objective response and may be the treatment of choice for patients with cirrhosis and a small tumour.

### F096

### REPEATED INTERMITTENT DEARTERIALIZATION IN THE TREATMENT OF LIVER TUMOURS

BO G PERSSON, BENGT JEPPSSON, K-G TRANBERG AND STIG BENGMARK.
Department of Surgery, Lund University, Sweden

Hepatic artery ligation or dearterialization may result in objective tumour regression but fails to provide prolonged disease control partly due to the promptness with which collaterals develop after permanent interruption, whether it comes from embolization or surgery. By performing intermittent blockade of the hepatic arterial circulation, formation of collaterals is reduced. Repeated intermittent dearterialization of short duration has been accomplished with the use of an implantable occluder accommodated around the hepatic artery.

Different occlusion periods have been investigated for development of collaterals and the effect on liver tumours have also been evaluated. Collaterals seem not to develop if occlusions are performed for less than 2 hours each day (primary and secondary cancers) or 16 hours every second month (liver carcinoid). Occlusions have been continued for 1-17 months (1-2 hours daily) and for more than 5 years in patients with liver carcinoid (16 hours). Nineteen patients have been treated according to this protocol (2 hepatocellular (HCC), 11 colorectal, 6 liver carcinoid). Both patients with HCC responded and 1 have had a sustained complete remission for 4 years. All patients with carcinoid metastases had symptomatic relief and responded with either reduction of measurable disease (3) or with reduction of urinary 5-HIAA (4) or both (3). In patients with liver metastases from colorectal cancer, who also received cyclic intraperitoneal infusion of 5-Fu, the effect was more variable with a crude response rate of 28%. Median survival was 17 months (range 2-23) for all patients, and seemed to be comparable to intrahepatic infusion but with much less toxicity.

An implantable occluder greatly facilitated the performance of repeated and transient occlusions and a complete arrest of the flow through the hepatic artery was generally achieved. Patient acceptance has been excellent and many of them have performed the occlusions by themselves. Continued occlusions have been possible for an extended period of time and complicaitons and toxicity have been few. Liver carcinoid (prolonged relief of symptoms and disease control) and HCC (especially with a concomitant cirrhosis) seems to be well suited for this treatment. Some patients with liver secondaries from colorectal cancer (vascular tumours) may have a beneficial palliation in combination with intrahepatic chemotherapy.

### F097

### SELECTIVE INTERNAL RADIATION THERAPY FOR TREATMENT OF INOPERABLE HEPATOCELLULAR CARCINOMA (HCC)

*W.Y LAU #W.T. LEUNG, #S. HO. ^N. LEUNG,#W. SHIU, *A.K.C. LI, ^C.METREWELI.
#Clinical Oncology, ^Medicine, Radiology and Organ Imaging, Prince of Wales Hospital, The Chinese University of Hong Kong, Shatin, Hong Kong

Eight patients with histologically proven, alpha-fetoprotein (AFP) >20ng/l, inoperable HCC were treated with either intra-arterial Yttrium-90 microsphere (Y-90) or Lipiodol-Iodine$^{131}$ (Lip-I$^{131}$). Five had Y-90 and 3 had Lip-I$^{131}$ treatment. Y-90 resin based microspheres have radioactive Y$^{90}$ attached to the matrix which measure 29 to 35 microns. Y-90 emits high energy electron (2.3 MeV) which has a half life of 64.2 hours. During laparotomy, cholecystectomy and insertion of a Port-A-Catheter into the gastroduodenal artery were done and Y-90 were injected into the common hepatic artery through the catheter. We had given various doses ranging from 2 GBq to 3GBq depending on the relative uptake of the tumour and normal liver which were measured intra-operatively by a beta probe and counter. All five patients had tumour regression and decrease of AFP to less than 20% of pre-treatment level. The average duration of hospitalization was 2 weeks. There was no side effects with respect to the internal radiation. The median survival of the five patients was 6 months from time of diagnosis. Three patients who had inoperable HCC diagnosed intra-operatively was offered LipI$^{131}$ treatment. Lip-I$^{131}$ is made by converting the iodine moiety of Lipiodol to I$^{131}$ through an atom-atom exchange method. Cholecystectomy and insertionof a Port-A-Cahteter were done in the usual way and on post-op days 12, 16, 20, 3 fractionated dose of Lip-I$^{131}$ were given by injection into the arterial port. The total dose ranged from 50 to 100mCi of I$^{131}$. Dosimetry was documented with serial gamma scans. Tumour regression and decrease of AFP level to less than 10% of pre-treatment level were again observed in 2 patients who had completed the course of treatment. The first patient survived 13 months from time of diagnosis and the other two are still surviving at 4 and 5 months respectively. Again, the treatment was relatively free of side effects and the duration of hospitalization was 3 weeks on average. In conclusion, selective internal radiation therapy is a feasible and effective treatment for inoperable hepatocellular carcinoma.

### F098

### EXPERIENCE WITH LIVER RESECTION FOLLOWING HEPATIC ARTERIAL CHEMO-EMBOLIZATION FOR HEPATOCELLULAR CARCINOMA

YE-QIN YU, DONG-BO XU, XIN-DA ZHOU, JI-ZHEN LU, PETER MACK
Liver Cancer Institute, Shanghai Medical University, Shanghai 200032, China

Thirty patients with huge primary liver cancers underwent percutaneous transcatheter hepatic arterial chemotherapy and embolization (THACE). The tumors were significant regression after THACE, converted them into resectable lesions, and successfully resected them afterwards. Patients received 1-5 treatment of THACE before surgery. The tumor diameters reduced by 31.6±15.2%(2.3±1.2cm). Tumor necrotic area ranged from 40-100%. Adhesions of the tumor to the surrounding tissues were the main operative findings but they do not significantly complicate the surgery. In 5 cases there was 100% tumor necrosis. In 7 patients, their AFP levels decreased to normal after THACE but of these, 5 still harbored cancer cells in the resected specimen. Hence, resection of the tumor is needed. The 1-,2-, and 3-year survival rates were 88.89%, 77.03% and 77.3% respectively. This mode of treatment appears promising for patients with advanced or initially considered as a huge unresectable liver cancer.

### F099

### HEPATIC CRYOSURGERY IN THE TREATMENT OF PRIMARY LIVER CANCER

X.D. ZHOU, Y.Q. YU, Z.Y. TANG, J.M. WENG,Z.C. MA, D.B. XU
Liver Cancer Institute, Shanghai Medical University, Shanghai, China

Cryosurgery with liquid nitrogen (-196°C) was performed on 87 patients with pathologically proven primary liver cancer (PLC) from November 1973 - June 1991. Of them, subclinical stage amounted to 31.0% (27/87), moderate stage 59.8% (52/87), and late stage 9.2% (8/87). There were 30 cases with small PLC (≤5cm). Liver cirrhosis was observed in 83.9% (73/87). Cryosurgery for PLC was mainly indicated for (1) patients associated with severe liver cirrhosis in whom hepatic resection would be contraindicated; (2) residual tumor at the cut surface or in the rest of liver after the main tumor resection; and (3) unresectable recurrent PLC after major hepatic resection. The liver was exposed by laparotomy. Flat surface cryoprobes were used to treat surface lesions. Single and multiple trocar cryoprobes were used for freezing tumors deep within the hepatic parenchyma. Intraoperative ultrasound was used to monitor hepatic cryolesions. The 1-year, 3-year, and 5-year survival rates were 60.5%, 32.0%, and 20.2%, respectively, for the whole series. Among the 30 patients with tumor nodules ≤5cm in diameter, the 1-year, 3-year, and 5-year survival rates were 92.6%, 66.6%, and 50.8%, respectively. There were no operative mortality and complications, such as rupture of tumor, delayed bleeding, bile leakage or abdominal infection. There results indicate that hepatic cryosurgery is a safe and effective treatment for unresectable PLC>

### F100

### PERCUTANEOUS CHOLECYSTOSTOMY IN THE TREATMENT OF ACUTE CHOLECYSTITIS IN HIGH RISK PATIENTS

M. MORENO; J.L. RAMOS; MA.A. DELGADO; R. FERNANDEZ; G. MENDOZA; P. ARIZAGA.
Hospital Universitario de Getafe. Getafe. Madrid.

Cholecystectomy is the treatment for acute cholecystitis, but in patients with sepsis and risk factors, such as age over 65 and other pathological conditions, operative mortality can reach up to 20%.

PATIENTS AND METHODS. From June 1986 to December 1990, 32 percutaneous transhepatic cholecystostomy (6-8F) have been carried out in these high risk elderly patients. Emergency drainage (within 24h.) because of septic shock was performed in 20 (62.5%) and delayed in 12 (37.5%) due to failure of conservative treatment. Average age was 76.03 y. Risk factors other than age appeared in all but one patient (87 y.).

RESULTS. Average lengths of drainage were 16.85 d. Complications related to the technique appeared in two cases (6.2%) of pericholecystic hematoma; three tube dislodgement (9.3%) and one catheter obstruction. Four tubes were relocated. Correlation between bile and blood cultures was highly significant (95.6%), *E. Coli* being the organism most frequently isolated (56.2%). Mortality was 6.2% (two cases) and non related to the procedure. Nineteen patients (59.4%) underwent delayed cholecystectomy. In these cases, morbidity was 5.2% and operative mortality was null. The eleven remaining patients refused or were not considered suitable for further surgery.

CONCLUSION. Percutaneous cholecystectomy can be an alternative procedure in the management in high risk patients with acute cholecystitis and sepsis because its morbidity and mortality can be favorable compared to emergency operations in such circumstances.

### F101

### SINGLE-DOSE CEFUROXIME VERSUS MULTIPLE-DOSE CEFAZOLIN FOR PROPHYLAXIS IN HIGH RISK CHOLECYSTECTOMY

K.R. SIRINEK, Y.E. YELLIN, B.A. LEVINE, T.V. BERNE, P.N.R. HESELTINE, M.B. APPLEMAN, M. GILL, K. PAPPA,
University of Taxas Health Science Center, San Antonio, Tx

The efficacy and safety of single-dose cefuroxime and multiple-dose cefazolin for surgical prophylaxis were compared in 295 patients undergoing biliary surgery in a prospective, double-blind, randomized, parallel-group study. Patients had at least one of the following risk factors: obesity, diabetes mellitus, acute cholecystitis, jaundice, hyperamylasemia; or they were ≥ 60 years old; or imaging evidence suggested the need for common duct exploration. Thirty to 60 minutes before the incision, patients received either cefuroxime 1.5 gm and then placebo every 6 hours for 3 doses (n=149) or cefazolin 1 gm and then cefazolin 1 gm every 6 hours for 3 doses (n=146). A bile culture was obtained. Patients were assessed daily while hospitalized and again within 30 days of surgery. Of the clinically-evaluable patients, no signs or symptoms of systemic or wound site infections were noted in 107 of 116 (95%) cefuroxime– and 111 of 117 (95%) cefazolin-treated patients (P=0.416, Mantel-Haenszel test). Of the bacteriologically-evaluable patients, 105 of 110 (95%) cefuroxime– and 110 of 112 (98%) cefazolin-treated patients were classified as bacteriological successes (P=0.248, Mantel-Haenszel test). Both drugs were well tolerated. We conclude that single-dose cefuroxime and multiple-dose cefazolin are equally effective for surgical prophylaxis in patients with risk factors for infection undergoing elective cholecystectomy.

### F102

### MRI DIAGNOSIS OF CHOLECYSTITIS

F.YAMAMOTO[1], YONGLIN PU[2], H. IGIMI[3]
Yamamoto Hospital, Imari, Japan[1]. Beijing Medical University, China[2]. Shionogi Institute, Osaka, Japan[3]

The utility of MRI in the diagnosis of cholecystitis was evaluated in 31 individuals (5 healthy, 13 with acute cholecystitis, and 13 with chronic cholecystitis). In the healthy volunteers the MRI scans were performed after an overnight fast for 12-14 hours, 2 hours after breakfast, 4 hours after lunch and 3 hours after supper. in all the patients MRI scans were performed randomly. Imaging was performed in all cases with the 0.5T RESONA unit. Spin-echo sequences were used. On the T1-weighted image (TR/TE=500/20, 620/25), T2-weighted image (TR/TE=1800/80, 2000/100), and proton-weighted image (TR/TE+1800/30, 2000/30) the liver/bile signal intensity contrast was significantly higher in acute cholecystitis than it was in chronic cholecystitis or in normal volunteers. There was no overlap in the 95% confidence intervals between acute cholecystitis and chronic cholecystitis or normal gallbladder on T1- and T2-weighted images. So in the clinical setting of acute cholecystitis the levels of 2.1 or more of the liver/bile contrast on the T1-weighted image and 0.384 or more on T2-weighted image can be used as diagnostic criterion for acute cholecystitis. The concentration of total protein, total bilirubin, cholesterol and fatty acid in bile in acute cholecystitis was significantly lower than that in chronic cholecystitis, which may be the cause of the high liver/bile signal intensity contrast in acute cholecystitis on all the MRI sequences. In acute cholecystitis there are some morphologic features known to be specific for the diagnosis of acute cholecystitis by CT and sonography that can also be detected by MRI: dilatation of the gallbladder, thickening of the gallbladder wall, wall and pericholecystic effusion and loss of wall sharpness. The MRI appearance of hemorrhagic cholecystitis was specific. On T1-weighted and proton weighted images in the gallbladder there is an irregular hyperintense area, and on the T2-weighted image the irregular area was hypointense in its center and hperintense in its periphery relative to the bile.

### F103

### ROLE OF KUPFFER CELLS IN HOST DEFENSE - TNF & IL-6 PRODUCTION IN SEPSIS

XU YING XIN, MENG XIAN JUN, SONG XUE HUANG
Institute of Basic Medical Science, The Great Wall Hospital, Beijing China

Liver, down stream from the gastrointestinal tracts, makes it the first line of defense. Hepatic Kupffer cells (KC) comprise 70% of the tissue-fixed macrophages. In sepsis KC become activated and produce a myriad of cytokines which can impact both locally on adjacent hepatocytes and systematically on distant target tissues. This study is designed to investigate production of tumor necrosis factor (TNF) and interleukin-6(IL-6) in relevance to the role liver plays in sepsis. Sepsis were produced by cecal perforation and perforation (CLP). At 5hr and 15hr after CLP, KC< peritoneal macrophage (PM), and alveolar macrophage(AM) were harvested and assayed for TNF, IL-6 activities and kinetics following stimulation with LPS> Results showed that for the same number of macrophages($2x10^6$) KC produced the highest IL-6 activity(107.10±6.44) 15 hr after CLP as compared to PM (74.96±6.68) and AM (44.26±5.44). AM produced the highest TNF activity 15 hr after CLP (54.69±8.16) compared with PM (14.06±2.51) and KC (17.32±1.62). At 15 hr after CLP, AM responded to stimulation with LPS by increased production of TNF but not IL-6. It is thus postulated that IL-6 is mainly confined in the modulation of hepatocyte function in acute phase response. When hepatic KC fails to clear endotoxins in the circulation, it may spillover to the lungs and induce pulmonary injury by TNF production.

### F104

### CHOLECYSTECTOMY CAUSES A RISE IN MEMEBRANE SATURATION

MW SCRIVEN, JCM STEWART*, DF HORROBIN*, MCA PUNTIS
Dept Surgery, University Wales College Medicine, University Wales Hospital, Cardiff, UK and *Efamol Research Institute, Kentville, Nova Scotia, Canada

The effect of surgical trauma on the saturation of membrane fatty acids (FA) was investigated.

Red cell phospholipid FA were measured perioperatively by gas chromatography, in 16 cholecystectomy patients (3M:13F, mean age 57). Results (molar%, median(IQR)) show saturated FA (SFA), monounsaturated FA (MUFA), n-6 polyunsaturated FA (PUFA) & n-3 PUFA. Statistics by Wilcoxon test comparing with preoperative:

ns=p>0.05, *p<0.05, **p<0.01.

| | PRE OP | DAY 1 | DAY 2 | DAY 4 |
|---|---|---|---|---|
| SFA | 41.4 (34.6-49.1) | 48.7 ns (36.9-54.6) | 52.1* (39.3-56.4) | 48.8 ns (36.5-55.9) |
| MUFA | 20.5 (17.5-25.0) | 24.5* (19.6-27.2) | 27.1* (18.3-28.1) | 22.6 ns (17.5-28.6) |
| n-6 | 28.2 (19.4-35.1) | 23.7 ns (17.1-32.1) | 16.2* (13.2-33.9) | 18.3 ns (13.0-33.2) |
| n-3 | 6.6 (2.7-12.3) | 2.6 * (1.5-9.5) | 1.0** (0.0-8.1 | 3.1* (0.9-10.9) |

This study shows significant changes in the saturation of membrane FA after surgical trauma. Such changes affect cellular function and if reflected in other cells, may explain some of the changes in cellular function that are known to occur after trauma. They may open up new modes of perioperative therapy.

**F105**

### SANDOSTATIN (SMS) IN THE LONG TERM MANAGEMENT OF PORTAL HYPERTENSION - A PRELIMINARY PROSPECTIVE RANDOMISED CONTROLLED CLINICAL TRIAL

SA JENKINS, S ELLENBOGEN, J BAXTER & R SHIELDS
University Department of Surgery, Liverpool, UK

Since SMS results in a sustained decrease in portal pressure, we have examined its efficacy as an adjuvant to injection sclerotherapy (IS) in the long term management of portal hypertension. Three weeks after their first variceal bleed, 32 cirrhotic patients underwent a thorough investigation of the severity of their liver disease, including assessment of reticuloendothelial system (RES) activity (single photon emission computed tomography), hepatocyte function (aminopyrine breath test) and wedged hepatic venous pressure (WHVP). Sixteen patients were randomised to IS and SMS, and 16 to IS alone. The efficacy of the two treatments were evaluated 6 months later. In patients receiving IS and SMS compared to those receiving IS alone there were significant reductions in mortality (0/16 vs 5/16; $p = 0.0434$ Fishers Exact Test), number of variceal rebleeds (16; $p<0.001$ U Test) and 0 vs number of IS sessions required to obliterate the varices (40 vs 89; $p<0.05$). Combined IS and SMS also resulted in a sustained decrease in WHVP (25.1± 1.3 to 19.2 ± 1.2 mm Hg $p < 0.002$) but significantly stimulated hepatic RES activity (19.8 ± 1.3 to 29.8 ± 1.5; % injected dose $^{99}$Tc sulphur colloid) and hepatocyte function (1.5 ±0.2 to 3.7±0.6% cumulative excretion 14 $CO_2$ breath test). No significant changes in WHVP, RES activity or hepatocyte function were observed in the IS group. These results suggest that SMS may be a valuable adjuvant to IS in the long term management of portal hypertension.

**F106**

### MESOCAVAL SHUNT VERSUS ENDOSCOPIC SCLEROTHERAPY FOR LONG TERM MANAGEMENT OF VARICEL BLEEDING

B ISAKSSON, B JEPPSSON, P HERLIN, S BENGMARK
Department of Surgery, Lund University, S-221 85 Lund Sweden

Sclerotherapy (ST) is usually different in controlling acutely bleeding oesophageal varices. For prevention of rebleeding it may not be as efficient as shuntsurgery and therefore we undertook a prospective study comparing mesocaval shunt (MCS) and repeated ST. Forty-five patients who had survived a haemorrhage from oesophageal varices were randomized to repeated ST (21 patients) or MCS (24 patients).

MATERIAL AND METHODS:after workup, randomization was performed. ST was performed every fourth month with flexible endoscop. MCS was performed with 12 mm graft.

RESULTS: There was an equal distribution according to Child's in the different group. There was no difference in survival in patients with Child's A and Child's B in the treatment groups. In patients with Child's C there was a statistically significant prolongation of survival compared to ST.

In the ST group 12 patients had recurrent haemorrhages causing 5 deaths compared with the shunt group in which 4 patients presented postoperative bleeding without associated mortality. There was no difference in the incidence of encephalopathy.

CONCLUSION: The rate of rebleeding is significantly higher in the ST group compared with the shunted group. In patients with Child's C cirrhosis MCS may be an alternative to ST for the prevention of bleeding for oesophageal varices in patients not suitable for transplantation.

**F107**

### AMMONIA CONCENTRATION IN PANCREATIC AND DUODENAL VENOUS BLOOD FOLLOWING STIMULATION:

CH. N. SBAROUNIS, I. FARDELAS, :D. KARVOUNARIS: N. MAVROUDIS
2nd Surg. Prop. Dept. Univ. of Thessaloniki, Greece

There are indications that ammonia is produced in the pancreas and duodenum, hypothesis that has not been proved. The aim of this experimental study was to investigate whether ammonia is also produced in the pancreas and duodenum and the possible factors that could influence this production.

Material and methods. Fifteen dogs, divided into 3 groups (A,B,C) were used. In all dogs the cranial pancreatic and the duodenal veins were catheterized selectively and the parameters measured were: ammonia, glucose and insulin in fasting state (A), following iv. infusion of hypertonic glucose (B) and iv. infusion of glucose with simultaneous stimulation of the pancreas with secretin (C). The levels of ammonia were compared to those of the systemic circulation and in pairs between the 3 groups.

Results. The concentrations of ammonia in pancreatic and duodenal venous blood are almost equal and are three times that of systemic circulation ($p<0.001$). The infusion of hypertonic glucose (group B) results in reduction of ammonia by one third in the pancreatic vein and by one fourth in the duodenal vein ($p<0.01$), with a slight reduction of circulating ammonia. The infusion of glucose and secretin (group C) had the same results with the infusion of glucose alone ($p<0.01$).

We conclude that ammonia is produced by the pancreas by a mechanism still not known. The relation of ammonia production to the bicarbonate excretion is not proved, since the stimulation of pancreas with secretin has no influence on the ammonia concentrations in pancreatic venous blood ($p<0.1$).

**F108**

### ENDOSCOPIC SCLEROTHERAPY COMPARED WITH OESOPHAGOGASTRIC DEVASCULARIZATION AND TRANSECTION IN THE LONG-TERM MANAGEMENT OF BLEEDING OESOPHAGEAL VARICES: A PROSPECTIVE RANDOMIZED CONTROLLED TRIAL

JEJ KRIGE, PA GOLDBERG, PC BORNMAN, J TERBLANCHE.
Department of Surgery and MRC Liver Research Centre, University of Cape Town and Groote Schuur Hospital, Observatory, Cape Town, South Africa

Fifty patients (34 male, mean age 46.1 years, range 18 - 65 years) with variceal bleeding were randomised after emergency endoscopic sclerotherapy to continued endoscopic sclerotherapy (ES) using 5% ethanolamine until variceal obliteration followed by regular check endoscopy or to oesophagogastric devascularization with transection (OGDT). Childs C score > 11, those over 65 yrs and high risk operative patients were excluded. Thrity-six pts had alcoholic cirrhosis; 7 were childs A, 24 Childs B and 19 Childs C. Mean follow-up was 16 months (range 3 - 46 months).

All data was analyzed on an intention to treat basis. Mortality during the first month after randomization was higher in the surgical group (2/25 v 0/25) but late deaths in the OGDT pts were fewer than among the ES group (4 vs 8). Varices were eradicated in 22 of 25 pts in the ES group after a mean of 5 injections (range 2-10). Three pts in the ES group died before eradication at a mean of 96 days. One patient in each group required dilatation for an oesophageal stricture. No pt died from rebleeding.

During follow-up there were no significant differences between the ES and OGDT groups with regard to number of pts rebleeding from varices (5 vs 6), number of bleeding episodes (8 vs 6) number of units of blood transfused per pt (2.9 vs 3.1), total number of days hospitalised (1203 vs 1143), total number of hospital admissions (79 vs 70), mean number of days in hospital per pt (52 vs 45) or mean days per admission (15 vs 16). We conclude that there is no significant difference between ES and OGDT and that ES is as effective as ODGT in eradicating varices and preventing rebleeding.

### F109

### MANAGEMENT OF BUDD-CHIARI SYNDROME (EXPERIENCE OF 260 CASES)

ZHONGGAO WANG, MD, FICA; SHIHUA WANG, MD; SHONGZHONG MA, MD
Beijing Heart Lung Blood Vessel Medical Centre, Vascular Institue, Post and Telecommunications Hospital, Beijing, China.

Budd-Chiari syndrome is a rare disorder with manifestation of portal hypertension caused by occlusion of hepatic veins (HVs) or suprahepatic inferior vena cava (IVC). However 260 such cases were diagnosed and treated by us in recent 10 years. There were 172 males and 88 females with a male to female ratio of 2:1. The age ranged from 2.5 to 65 years with an average of 33 and symptoms were present from between 3 days to 26 years. All cases were confirmed by ultrasonography, cavography or hepatovenography. Most of patients (249 cases or 95.8%) had occlusive lesions involved the suprahepatic IVC.

Various procedures were performed in 217 cases including mesoatrial shunt (53), membranotomy (44), cavoatrial shunt (53), balloon catheter dilatation (22), radical correction (20), mesocaval shunt (11), mesojugular shunt (5), combined innominate-atrial shunt (2) , splenoatrial shunt (1), etc.

The overall effective rate was 77.4% and operative mortality of 7.3%. The 5-year patency rate of the mesoatrial shunt, membranotomy and cavoatrial shunt were 71.4%, 66.7%, and 50.0% respectively.

According to our experience, the therapeutic approaches are basically described as follows: 1. Balloon dilatation is the first option for those with webs or localized occlusive lesions without distal fresh thrombi. 2. Transcardiac membranotomy is also suitable to the above-mentioned patients. 3. Cavoatrial shunt is used for those with diffuse occlusive leisions in the IVC with involvement of the HVs. 5. Portocaval or mesocaval shunts are employed for those with pure occlusion in the HVs. 6. Mesojugular shunt is a good alternative for those with intractable ascites, pleural effusion, and high operative risk. 7. Innominate-atrial shunt can be supplemented in those accompanying with superior vena caval syndrome. 8. Radical correction is best indicated for those whose lesions require extirpation. 9. Liver transplantation reserves as the last option.

### F110

### MANAGEMENT OF HIGH BILE STENOSIS AND OCCLUSION WITH THE RODNEY-SMITH METHOD. REPORT ON 34 CASES.

ANDRIOPOULOS ATHANASE, R. BOTTICHER, HACK OBERARZT
Surgical Department I, Klinikum Furth Jakob-Henle-Str. 1, Germany

Surgical treatment of extrahepatic stenosis located at liver hilus due to biliary carcinoma or strictures following previous surgery or trauma represent some special problems. Difficult preparation of extrahepatic bile tree especially in case of reintervention, inflammatory tissues, liver insufficiency with cholostasis, clotting disorders and a high rate of anastomotic leakage or shrinkage are undesirable life threatening conditions.

In such cases a sutureless hepato-jejunostomy with transhepatic intubation using the Rodney-Smith method is a very effective technique to repair high bile occlusions.

In the last ten years we used this method in 34 cases. Carcinoma of the head of pancreas, posttraumatic lesions of the hepatic duct, strictures after duodeno- or cholodocho- jejunostomy, stenosis due to cicatrix after stomach-resection with insufficient duodenal stump, primary carcinoma of hepatic duct or stenosis after bilio-digestive anastomosis were indications for using the Rodney-Smith technique.

The transhepatic tube is normally moved after X-ray control within 3 – 5 month. In case of severe jaundice with all its sequelae a preoperative percutaneous cholangiography is recommended with remaining catheter till liver function tests turn to levels permitting surgery.

Aprotinin was always administered to minimize blood loss during the last two years. Preoperative antibiotic prophylaxis with mezlocillin or therapy according to antibiogram was routinely used to prevent bacterial complications.

No postoperative deaths occurred, no complications required reintervention. In five cases repeated replacement of the tube was necessary to dilatate restenosis due to malignancy. This procedure was easily and always successfully achieved by pushing the tube externally beyond the stenosis unter X-ray control.

### F111

### ENDOSCOPIC PALLIATIVE STENTING OF MALIGNANT HILAR STRICTURES

PPLO COENE, K. HUIBREGTSE, HFW HOITSMA, MN vd HEYDE, GNJ TYTGAT.
Dept. of Gastroenterology and Surgery, Academic Medical Center, Amsterdam, The Netherlands

Endoscopic stent insertion has been shown to be an effective palliative therapy for establishing biliary drainage in malignant obstructive jaundice. Metastasis, multifocal disease, or extensive invasion may prohibit curative resection in most patients, and those who are unfit for sugery because of advanced age, extensive metastasis, or other unrelated illnesses should be considered for nonsurgical biliary drainage. Success of drainage procedures are related to the level of biliary tract obstruction, hilar malignancies being less successful overall, compared to distal strictures. An in depth analysis was established to evaluate factors which influence stent function and clinical results, in 257 consecutive stented patients (male/female ratio 0.70; mean age 69 years, range 28-92 years), followed up over a 10-year period (cumulative success rate 85%, mean 1.2 ERCP).

Hilar strictures comprised a heterogeous group of malignant tumors (23% of all malignant biliary strictures) including sclerosing cholangiocarcinomas originating at the confluence of the left and right hepatic ducts (n= 163, or 64%; Klatskin tumor), locally invasive gallbladder cancer (n=34, or 13%) as well as metastasis to the porta hepatis (n=60, or 23%). Sixty three patients had type I, 72 type II, 62 type III, and 60 type IV proximal biliary strictures (Bismuth classification). Mean bilirubin 213 u mol/1, mean alkaline phosphatase 566 units/1. Classification of bifurcation tumors revealed a significant correlation ($p<0.01$) between length and type (according to Bismuth) of lesion, and respective bilirubin decline (overall 86%), bilirubin normalization (overall 66%), early cholangitis (type I 17%, type II 17%, type III 29%, and type IV 40% ; overall 25%), procedure related mortality (overall 9.5%), 30-day mortality (type I 14% , type II 17%, type III 32%, and type IV 34%; overall 23%), patient survival (type I lesions median 137 days, type II 120 days, type III 77 days, and type IV 43 days; overall mean 192 days, median 95 days), and terminal clinical features (fever 31%, jaundice 74%). There was no difference in survival between primary cancer (median 94 days for cholangiocarcinoma, and 95 days for gallbladder carcinoma) and metastatic cancer (median 98 days). Acute cholangitis was the most important early complication, directly related to the number of endoscopic procedures and caliber of endoprostheses, and occurred in 23% of patients having one stent (85%), compared to 39% of those with drainage of both liver lobes by at least two stents (15%; $p<0.01$). Median stent patency was 175 days, overall incidence of stent change 30%. The total number of stents required was directly proportional to patient survival time (mean number 1.5, range 1-14). Cholangiographic appearance (type I-IV, $p=0.14$), number of simultaneous placed stent (one or multiple, $p=0.91$), or extent of drainage (one or both obstructed lobes, $p=0.65$) did not influence stent patency.

Bifurcation strictures of both primary and secondary malignant origin can be successfully treated with single stent insertion with reasonably low risk of cholangitis and low mortality, and with a minimum number and duration of endocopic procedures. However, patients with multiple intrahepatic strictures and with extension of tumor into the segmental ducts, in the absence of unbearable symptoms, should not be selected for stenting procedures, because the benefits of drainage do not balance the risks of complications.

**F112**

### LOCAL EXTENTION OF PANCREATIC AND PERIAMPULLARY TUMORS IN 55 SPECIMENS AFTER PYLORUS PRESERVING PANCREATECTOMY

A.PIETRABISSA, U.BOGGI, P.C.GIULIANOTTI, G.SARTONI, T.BALESTRACCI, A. COSTA, A.GUADAGNI, *G.FORNACIARI, *J.BRUNO, F.MOSCA
Instituto di Chirurgia Generale e Sperimentale,
*Instituto di Anatomia Patologica Universita de Pisa, Italy

Between Jan. 1982 and Jun. 1991, 146 patients underwent a pancreatic head resection at our Institution. Among the 116 who had a diagnosis of a tumor of the pancreatic area, 108 received a pylorus preserving procedure. We report pathologic features of the last 55 cases of adenocarcinoma whose surgical specimens were carefully analyzed focusing on local extention of the tumor and lymph node involvement. The study comprised 33 tumors of the head of the pancreas, 14 of the papilla, 4 of the CBD and 4 of the duodenum. Each gross specimen consisted of the head of the pancreas and a variable lengh of duodenum, always transected 2 cm. away from the pyloric ring. The tumor ranged in size from 2 to 7 cm. (average 4.1) and on microscopic examination the pancreatic margin, judged negative on frozen section, resulted to be involved by small foci of tumor in 3 cases. The duodenum was infiltrated in the area directly adjacent to the tumor in 42% of cases, while in no instance the proximal duodenal resection margin was involved by tumor spread. An average of 9+/-8 regional lymph nodes for each patient were available for pathologic examination (range 1-28) including peripyloric and celiac nodes. Evidence of tumor was shown in 36% of cases with an average of 3+/-2 lymph nodes involved in each case (range 1-8). The adequacy of the pylorus preserving procedure as a cancer operation is stressed by the same survival figures obtained with the Whipple and we showed evidence that a microscopic extention to the duodenal surgical margin seems very unlikely to occur and therefore should not be considered as a theoretical limitation of the procedure. In addition preservation of the pylorus does not compromize the possibility to perform an extended regional limphoadenectomy. Our experience suggests that an intraoperative conversion to a standard Whipple operation for a cancer procedure should be undertaken only facing with obvious spread of the disease ot proximal duodenum or gastric wall.

**F113**

### PANCREATIC AND PERIAMPULLARY CANCER IN THE ELDERLY: SHOULD IT BE RESECTED?

P.C.GIULIANOTTI, T.BALESTRACCI, U.BOGGI, A.COSTA, A.PIETRABISSA, A.GUADAGNI, M.FERRARI, F.MOSCA
Istituto di Chirurgia Generale e Sperimentale, Università di Pisa, Italy

The progressive increase of pancreatic and periampullary cancer incidence in the western population, with a peak in VI VII decades, together with the elderly increase, arises new problems in the surgical approach to these patients.

We performed a retrospective review of the data records of 21 pts. over 75 years (mean age 79.5+/-3.4, 11m-10f) who underwent pancreato-duodenectomy (PD) for pancreatic or periampullary malignancy from Jan. 1982 to Jun. 1991 (group A) to evaluate both post operative morbidity and mortality rates and post operative quality of life. We compared them either with 98 pts under 75 yrs. (64m-34f, mean age 63.7+/-11.01) who underwent PD with the same diagnosis (group B), and with 35 pts. over 75 yrs. who received only a surgical palliative procedure (20m-15f, mean age 79.37+/-3.7)(group C).

In group A we had 13 pancreatic carcinoma (PC), 5 ampullary carcinoma (AC) and 3 common bile duct carcinoma (CBDC); 11 pts. had obtructive jaundice (mean bilirubin 10.8+/-12.5 mg/dl). 5 pts. with a more severe jaundice (mean bilirubin 19.12+/- 12.8 mg/dl) and signs of metabolic failure, underwent preoperatively percutaneous transhepatic drainage. Additional risk factors were: ischemic cardiopathy (3 pts), essential hypertension (2 pts), diabetes (3 pts). One bleeding pancreatic cancer, infiltrating the duodenum, demanded emergency operation. In group B we had 62 PC, 21 AC, 9 CBDC, 5 duodenal carcinoma and 1 retroperitoneal limphoma and in group C 33 PC, 1 AC and 1 carcinoid.

We have never considered the age (over 75 yrs) as an exclusion criterion for radical surgery. Operative morbidity was 47.60% (40.8% group B; 50% group C) and operative mortality for elective surgery, excluding the patient operated on emergency who died for MOF, 4% (1 stroke) (11.5% group B; 8.5% group C). the survival time ranged from 4 to 74 months (median 11.1) in group A, while the median was 15.75 and 5 months in group B and group C respectively.

Patients in group A as like group B pts. had a better quality of life than group C pts. (time free from disease, possibility to resume a normal physical activity, and lower need of analgesia).

We conclude that age is not an absolute exclusion criterion from radical surgery which remains the only chance of cure and offers the best way of palliation in the pancreatic and periampullary cancer.

**F114**

### PYLORUS PRESERVING PANCREATODUODENECTOMY Vs WHIPPLE PROCEDURE FOR PANCREATIC CANCER: A STATISTICAL ANALYSIS OF SURVIVAL

P.C.GIULIANOTTI, *G.ROSSI, U.BOGGI, T.BALESTRACCI, A.PIETRABISSA, A.COSTA,
Istituto di Chirugia Generale e Sperimentale, *Dipartimento di Epidemiologia e Biostatistica C.N.R. Università di Pisa, Italy

Doubts have been raised as to the advisability to perform the pylorus preserving pancreato duodenectomy (PPPD) for malignant disease of the pancreas, since it could compromise the only chance of cure in these patients.

We therefore undertook the present retrospective analysis to compare the outcome following the PPPD with that of the classical Whipple resection in 57 pts. with histologically proven adenocarcinoma of the head of the pancreas.

PPPD n = 41 (25m-16f mean age 64.5+/-10.1, TNM stage : I [n=21] II [n=5] III [n=15] Whipple n=16 (10m-6f mean age 62.7+/-7, TNM stage: I [n=9] II [n=0] III [n=7]). The variables examined were : age, sex, stage, T and N status, TNM grading, death for recurrence and survival time. Maximal follow-up was chosen of three years. All pts. underwent the same post-operative treatment. No pt. was lost at follow-up: 2 pts. (PPPD) died of cardiovascular disease, without neoplastic recurrence. Patients alive with a follow-up less than three years were considered censored. The statistical software used to handle censored survival data was : life table and survival functions (Breslow and Mantel-Cox Test), Cox Proportional Hazard Regression Model (three different tests were utilized to assess the treatment effects while adjusting for baseline pt. characteristics : L ratio, Wald Test and Score Function Test). T Test, Exact Fisher Test (2-tail) or X2 were used to assess differences between the 2 groups for baseline characteristics. The two groups were comparable with respect to all the variables examined. The analysis showed a statistically significant relationship between TNM stage (P<0.05) N status (P<0.05) and survival time. Survival time was not statistically different between PPPD and Whipple: median survival time was 14+/-1.9 and 13+/-3 months respectively. No statistical difference appeared even after correction for TNM stage and N status using the Cox model.

On the basis of these results PPPD may be propsed as a cancer procedure even when dealing with pancreatic carcinoma, except for those cancers that are in close proximity to the first portion of the duodenum.

**F115**

### PRE-OPERATIVE EVALUATION OF LIVER LESIONS; MAGNETIC RESONANCE IMAGING (MRI) VERSUS CT-ARTERIOPORTOGRAPHY (CTAP)

M.S. van LEEUWEN, J.C.M.HAVERBUS, E.D.DILLON, H. OBERTOP
Departments of Diagnostic Radiology and Surgery, University Hospital Utrecht, The Netherlands.

In order to determine the best imaging modality for preoperative evaluation of liver lesions, the results of MRI and CTAP studies were compared to each other and to surgical and pathology findings. A total of 24 patients, referred for liver resection were evaluated; 18 with metastatic colorectal cancer, 5 with primary livertumour, and 1 with FNH. All 24 patients underwent MRI; 20 out of 24 underwent CTAP, the remaining 4 had conventional CT>All lesions were localized according to Couinaud's classification and characterized as solid, cyst, or hemangioma. The results were compared to the "gold standard" , defined as the combination of imaging, surgery and pathology findings.

A total of 142 solid lesions were present, distributed over 95 liversegments. Furthermore, 9 hemangioma's were present. MRI detected 136 of 142 solid lesions leading to a sensitivity of 95%. MRI detected solid lesions in 90 of 95 diseased segments, i.e. 94%. In the 20 patients who had CTAP 84 solid lesions were present in 71 segments. CTAP could only identify 57 of 84 lesions, i.e. 68%. Lesions were found in 66 of 71 diseased segments, but in only 50 of 71 the lesions could be called solid with certainty, leading to a sensitivity of 70%.

Ni of 24 patients were inoperable due to extensive tumorspread. The remaining 15 patients were operated on. Ten had a successfull resection while 5 turned out to be irresectable; 3 due to extrahepatic disesase, 1 due to an unrecognized lesion in the left lobe and 1 due to encroachment on the hepatic vein confluens.

In conclusion; MRI is more useful than CTAP in detecting, localizing and characterizing lesions prior to liver resection.

### F116

### SIMULTANEOUS COMBINED PANCREAS AND KIDNEY ALLOTRANSPLANTATION IN THE PIG

P.G.SFIKAKIS, P.J.TZARDIS, R.W.G.GRUESSNER, D.E.R. SUTHERLAND
1. Dept. of Surgery, University of Minnesota, Minneapolis Minnesota 55455, U.S.A.
2. Dept. of Surgery, Red Cross Hospital, Athens, Greece

We established a model of en bloc simultaneous pancreas and kidney tranplantation that decreases preservation time, operation time and clamp time. Our method was developed on 32 Yorkshire pigs with the following technique : The donor aorta - with celiac axis, superior mesenteric artery and left renal artery - is anastomosed en bloc to the recipient's aorta in a side-to-oblique fashion. The portal vein is anastomosed end-to-side to the distal vena cava, and the left renal vein end-to-side to the left common iliac vein. The donor duodenum is anastomosed to the bladder to allow monitoring of the urinary amylase for rejection.

The proposed technique facilitates the vascular reconstruction saving one arterial anastomosis and additionally saves about 50min of operating and clamp time. None of our recipients has died from a technical complication.

In conclusion, we believe that this technique could be used in humans, especially in adult uremic diabetic patients who receive a combined pancreas/kidney allotransplant from a pediatric cadaver donor.

### F117

### THE PATTERN OF PLASMA CHOLESTEROL ESTER FATTY ACIDS IS ABNORMAL IN OBSTRUCTIVE JAUNDICE

MW SCRIVEN, JCM STEWART*, DF HORROBIN*, MCA PUNTIS
Dept Surgery, University Wales College Medicine, University Wales Hospital, Cardiff, UK and *Efamol Research Institute, Kentville, Nova Scotia, Canada

We have investigated the effect of obstructive jaundice (OJ) on plasma cholesterol ester (PCE) fatty acids (FA).

PCE FA were measured in 42 patients with OJ (mean age 65, 19F: 23M, mean bilirubin 247µmol/1, 12 benign : 30 malignant) and 42 matched controls, by gas chromatography. The results were compared by Mann-Whitney U tests.

Many FA were abnormal in OJ. Overall the mean number of double bonds per FA was lower in the OJ group ($p<0.001$). This was a reflection of a fall in total polyunsaturated FA (PUFA) ($p<0.001$) and a rise in total monounsaturated FA ($p<0.001$). Total saturated FA were not different between the two groups. The change in PUFA occurred in both the major series of PUFA - n-6 PUFA ($p<0.001$) and n-3 PUFA ($p<0.002$).

This study has demonstrated marked changes in FA esterified to cholesterol in plasma in OJ. Such changes, in particular the shift towards more saturated FA, are known to impair cholesterol transport. The biological significance of these changes is unclear but they provide further evidence of the profound metabolic effects of obstructive jaundice.

### F118

### SURGICAL TREATMENT IN HYDATIC LIVER DISEASE

BERCEDO J, LOINAZ, C.JIMENEZ C, G-URENA M, PALMA F, ALVARADO A, LOPEZ A, MORENOE.

The experience of surgical treatment in hydatidosisin our service is analysed, studying the changes in our technique and results.

Since 1974-1989 410 patients with 561 cyysts were operated. We divided the period of time in two groups: GROUP A : since 1974 to 1984 (322 patients), and; GROUP B: since 1985-1989 (88 patients). We compared both groups and the total resections techniques vs partial ones. Both groups were homogeneous in age and sex.

The incisions made were: right subcostal laparotomy (A=30.7% B=43.7%); thoracophrenolaparotomy (A=25.8% B=5.7% $p<0.001$); bilateral subcostal (A=12.7%) B=32.2% $p<0.001$) supraumbilical median laparotomy (A=16.5% B=4.6% $p<0.05$) supra and infraumbilical (A=3.1% B=9.2% $p<0.05$). The surgical procedure was : hepatic resection (HR) (A=6.5% B=15% $p<0.05$); total cystopericystectomy (TQPQ) (A=64.9% B=57.5%) partial cystectomy (PQ) (A=26.3% B=27.5%); cysto-jejunostomy (A=2.1% B=0%). The total mortality was : A=2.4% B=2.1% Mortality, morbility and reoperations are shown in the Table.

The low morbi-mortality of resections techniques vs PQ is shown, so that we think total resections must be used in the hydatid liver disease (if it is possible).

| | MORTAL | FISTULA | ABCESS | REOP. | NUMBER |
|---|---|---|---|---|---|
| TQPQ | 1.15% | 3.08% | 6.15% | 3.08% | 63.4%(260) |
| PQ | 4.28% | 17.0% | 12.1% | 8.5% | 26.5%(109) |
| HR | – | 2.9% | 8.8% | – | 8.2%(34) |

$p<0.05$ between TQPQ vs QP; and between TQPQ and HR vs PQ

### V001

### REOPERATION FOR RECURRENT HEPATIC HILUM NEOPLASM

G.M. GAZZANIGA, G.BONDANZA, M.FILAURO, C.BAGAROLO, F.ERMILI
1st Department of Surgery - S.Martino Hospital, Genoa - Italy

The video shows the case of a 52 years old man, operated for klatskin tumor in an other hospital one year before. He complaints episodes of severe cholangitis, jaundice, pruritus, weight loss; pro-operative back up showed dilatation of intrahepatic biliary tree, due to obstruction of the previously confectioned hepatico-jejunostomy at the hilum. During reoperation, a tumor recurrence on the anastomosis was found, with a infiltration of externale layer of portal bifurcation. Hepatectomy was not advisable for the presence of multiple, small abscesses in the right lobe, and for small size of left lobe. Resection of intrahepatic right and left hepatic ducts, distal part of Roux-en Y-jejunal loop (with the anastomosis) was performed, in order to arrive in a free-of tumor biliary tract-resection and reconstruction of portal bifurcation was performed with an extended regional lymphadenectomy too. Biliary flow restoration was obteined with a multiple cholangiojejunostomy between second oder biliary ducts on the right and left lobe, and Roux-en Y-jejunal loop; fibercolangioscopy was performed in the post operative period (to check patency of anastomosis) using transhepatic tracks created by tranparenchimal biliary stents positioned during operations.

